# Genetically encoded biosensor enabled mining, characterisation and engineering of aromatic acid MFS transporters

**DOI:** 10.1186/s13036-025-00568-y

**Published:** 2025-10-31

**Authors:** Philip Le Roy, Micaela Chacόn, Neil Dixon

**Affiliations:** Manchester Institute of Biotechnology (MIB), Department of Chemistry, University of Manchester, Manchester, M1 7DN UK

**Keywords:** Biosensors, Major facilitator superfamily, Protocatechuic acid, Terephthalic acid, Syntenic analysis, Aromatic acid transporters

## Abstract

Active transport of chemical species across the cell membrane represents a critical biological and biotechnological function, allowing the cell to selectively import compounds of nutritional value whilst exporting potentially toxic compounds. Major facilitator superfamily (MFS) transporters represent a ubiquitous class able to uptake and export an array of different chemical species. When designing biosynthetic pathways within microbial hosts, for production or remediation, transport is often critical to the efficiency of the resulting engineered strain. However, transport is a commonly neglected node for characterisation and engineering given difficulties in producing, purifying and assaying membrane transport proteins outside of their native environment. Here, using syntenic analysis and genetically encoded biosensors a library of 11 TphK and 10 PcaK homologs were screened for their ability to uptake the aromatic acids, protocatechuic acid and terephthalic acid. The structure activity relationships of the corresponding PcaK and TphK transporter-biosensor constructs, were then assessed with a library of aromatic acid effectors. Finally, the feasibility of protein engineering was assessed, by the creation of chimeric MFS transporter-biosensor constructs, revealing a degree of effector recognition plasticity and the modularity of core transmembrane domains. This study provides a library of validated TphK and PcaK homologs and demonstrates the value of employing genetically encoded biosensors in the characterisation and engineering of this important transport function.

**Supplementary Information:**

The online version contains supplementary material available at 10.1186/s13036-025-00568-y.

## Background

Microbial cell factories are foundational to the future bioeconomy, permitting the sustainable production of chemicals and materials from renewable and waste feedstocks. Efficient utilisation of these feedstocks is essential for process economics; however, this can be challenged by the compositional and chemical heterogeneity of an input stream. Here, the poor import of feedstock-derived substrates can impose large bottlenecks on strain productivity due to insufficient intracellular concentration of the substrate or through an inability to efficiently remove toxic compounds from the intracellular space. To overcome this, transporter engineering aimed at optimising the movement of substrates and intermediates across the microbial cell membrane and the coupling of transport to other cellular processes constitutes an important facet of strain development [1–4]. Further, engineering transporters for broader substrate scope can be used to expand the range of metabolically accessible compounds, permitting more complete utilisation of feedstocks and greater conversion efficiency [5]. Transport proteins are immensely diverse both in terms of structure and function, and are classified into many families based on these traits [6]. For the purpose of biomolecular engineering approaches concerning waste feedstocks such as lignocellulose however, this pool can be narrowed to the major facilitator superfamily (MFS) [7–13], ATP binding cassette (ABC) [14–17] family, tripartite ATP independent periplasmic (TRAP) transporters [18], and ion transporter superfamily (IT) [19] for lignin aromatics based on experimentally confirmed uptake of lignocellulosic substrates by these transporter classes.

The MFS is the largest and most diverse family of secondary active transporters. This superfamily can be further categorised into 16 families and 89 subfamilies based on phylogeny and substrate scope [20]. The substrate scope of MFS’ encompasses a diverse array of substrates, such as: sugars (sugar porters), inorganic or organic anions or cations (anion: cation symporters), aromatic acids (aromatic acid symporters/exporters) and drug/hydrophobic substances (drug: proton(H^+^) antiporters) [20]. Their ability to import and export valuable substrates such as sugars and organic acid make these transporters attractive targets for microbial strain engineering [1, 21–23]. In addition to their valuable function, MFS proteins are generally quite small, typically consisting of 400–600 amino acids comprising 12–14 transmembrane helices, in addition to being driven by ion gradients instead of ATP hydrolysis, resulting in low cellular burden. More than three-quarters of transmembrane containing proteins, such as those belonging to the MFS class, are functionally unclassified, despite these proteins accounting for more than 20–30% of the total number of any one proteome [24]. Even in characterised model organisms, such as *Escherichia coli*, 53% of membrane transporters lack characterisation [6]. This is due to two major factors, firstly, the functional flexibility/redundancy of transporters makes assignment of sequence-function relationships challenging to determine [25]. As such, protein sequence homology-based approaches to for the discovery of transporters alone is ineffective [26]. Indeed, MFS transporters are known to display particularly poor sequence conservation, with identity typically ranging 12–18%, between members despite the conserved MFS fold; sequence [27]. Secondly, transport proteins within the MFS are inherently unstable outside of their native phospholipid environment and thus require specialist approaches for expression, purification and characterisation [20]. Direct vectorial measurement of transport requires labelled substrates, which can become prohibitively expensive for mid to high-throughput screens. For MFS’ that rely on H^+^ symport, cheaper assays that utilise pH sensitive dyes are available, however these dyes can lack sensitivity for transporters with low turnover [20].

MFS transporters of the aromatic H^+^ symporter class have been demonstrated to be critical for the uptake lignocellulose-derived substrates, in particular for lignin an aromatic rich component with great potential as a feedstock for microbial valorisation [25]. Lignin is highly enriched in hydroxycinnamoyl aromatics such as *p-*coumaroyl (H), coniferyl (G) and sinapyl (S) alcohols with acid derivates such as coumaric, ferulic, and synapic acid respectively liberated following treatment of the lignin [28]. Saprophytic strains of bacteria such as *Pseudomonas putida* KT2440 (*P. putida*), *Rhodococcus jostii* (*R. jostii*) RHA1, and *Sphingobium* sp. SYK-6 have evolved pathways for the uptake and subsequent utilisation of these aromatics for growth [28]. Of these pathways, coumaric and ferulic acid ultimately converge on protocatechuic acid (PCA) as a central node in aromatic catabolism before being directed to the tricarboxylic acid cycle (TCA) cycle [28]. Disruption of these aromatic catabolic pathways in hosts, can lead to the accumulation of value-added intermediates, such as vanillin and 4-hydroxybenzoic acid, protocatechuic acid (PCA) and β-ketoadipate [29–32]. Another aromatic-rich polymer, of anthropogenic origin, is polyethylene terephthalate (PET) plastic, which can microbially degraded and has been proposed as potential feedstock for microbial growth and bioproduction [33]. PET represents 12% (by weight) of total global solid waste [34], and is composed of a repeating unit of terephthalic acid (TPA) esterified to ethylene glycol (EG), and finds its primary use in the manufacture of single use disposable packaging such as in bottles, clothing and food containers [35]. Recently, several bacteria including *R. jostii* RHA1, *Ideonella sakiensis*, and *Pseudomonas umsongensis* (*P. umsongensis*) amongst others, have been reported to possess the ability to import and utilise TPA, for growth, following import via a H^+^ symporter MFS transporter opening up the possibility of applying this waste stream as a substrate for biotechnological processes [36, 37].

The MFS-dependent uptake of PCA and TPA has been reported to occur via the action of two MFS transporters, PcaK and TphK (also known as TpaK in other literature, but referred to as TphK here from now onwards), respectively [11, 38, 39]. Strains deficient in PcaK universally exhibit reduced growth on PCA, while those deficient in TphK exhibit no growth on TPA [38, 40, 41]. Characterisation of PcaK using proteoliposomes has indicated this transporter has a substrate preference for PCA and 4-hydroxybenzoic acid (4HBA) [42], while there are no known reports for purification or in vivo characterisation of TphKs in the literature that we are aware of. Both transporters have demonstrated value in microbial strain engineering applications, with overexpression of the native *pcaK* from *Sphingobium* sp. SYK-6 enabling 30% higher conversion rate of PCA to the plastic precursor, 2-pyrone-4,6-dicarboxylate [10]. As well, heterologous expression of a *tphK* from *Pseudomonas mandelli* (*P. mandelli*) in *P. putida* permitting *de novo* production of the plastic polymer, polyhydroxyalkanoate (PHA), from a TPA and EG co-feed has similarly been used in the production of β-ketoadipic acid [38, 43]. Approaches such as these highlight the value of considering cellular transportation during strain engineering workflows. Furthermore, due to MFS proteins being a single polypeptide unit they do not require the stoichiometric balancing of other transporter subunits to correctly function. The structural uniformity of MFS proteins therefore lends itself well to engineering efforts, as adversely effecting other domains does not need to be considered. Collectively, this indicates that MFS transporters are ideal targets for use in heterologous whole cell factory hosts, and are amenable to protein engineering approaches [5, 44, 45].

For the technical challenge set out above, screening strategies that utilise facile, widely implementable synthetic biology techniques are an attractive alternative to mediate the high throughput identification and substrate specificity determination of transport systems. Small molecule responsive genetically encoded biosensors, comprised of allosteric transcription factors and riboswitches, can be coupled to optically detectable proxies (e.g. fluorescence) or cell survival in order to elucidate phenotypic responses. The amenability of such biosensor-based assays to high throughput screening has proven effective and highly selective for identifying optimal phenotypes in enzyme mutant libraries, and for the enrichment of strains with improved metabolic efficiency toward a desired product [46, 47]. The application of biosensors towards the characterisation of transporters has already been demonstrated with these systems complimenting methods that rely on large genomic disruption libraries or heterologous expression. Using a CRISPR based method of genomic disruption a knockout library of transporters was developed in *Saccharomyces cerevisiae* (*S. cerevisiae*) totalling 361 transporter deletions [48]. Through integration of a catabolic cascade to convert glucose to *cis*,* cis-*muconic acid (cc-MA) and a previously developed cc-MA biosensor active in *S. cerevisiae*, screening of the deletion library was assessed via fluorescence activated cell sorting, identifying *tpo2* as responsible for the uptake of cc-MA and PCA [48]. Further, a thiamine pyrophosphate responsive riboswitch was used in conjunction with metagenomic screening to identify several members of a new class of thiamine transporter, PnuT. The riboswitch was then replaced with one responsive to xanthine alkaloids to mine for xanthine importers, demonstrating the flexibility of the biosensor based approach [26].

In this study we demonstrate the application of genetically encoded biosensors for the identification and characterisation of aromatic acid H^+^ symporter MFS transporters with specificity towards two molecules of biotechnological significance, PCA and TPA. Syntenic genome context analysis was initially performed, using catabolic genes/operons as the search query, to identify genomically associated PCA and TPA transporter homologs (encoding PcaKs and TphKs, respectively). Transporter activity was verified using either a PCA or TPA responsive transcriptional biosensor coupled to GFP reporter output. Transporter substrate scope was explored via effector panel screening, and the generation of chimeric transporters which were constructed and assessed to better understand structure activity relationships of the two types of MFS and ability to enable non-cognate substrate transport functions.

## Methods

### Phylogenetic analysis and homolog identification

Preliminary searches for TphK homologs were performed via Position-Specific Iterated BLAST (PSI-Blast) using the Blastp suite hosted on the National Center for Biotechnology Information (NCBI) server with the Ref Seq database using the *R. Jostii* RHA1 TphK (Uniprot ID: Q0RWE8_RHOJR, (annotated as TpaK)) protein as a query sequence. Sequences resulting from this search were extracted and visualised via Jalview [49] and multiple sequence alignment was performed utilising MUSCLE. IQtree was used to create an initial phylogenetic tree which was later visualised using iTOL. To query the entire NCBI database, the cblaster python package [50] was used in remote mode with the *R. jostii* (NC_008270, Region: 190337–196425) TPA catabolic operon. Clusters were filtered by the presence of an MFS gene being present in the operon followed by the extraction of the MFS protein sequences. The Cdhit package [51] was used to remove redundant and duplicate sequences with a threshold of 0.95 (95%), followed by alignment and visualisation as stated above. PcaK searches were split according to the type of ring cleavage mechanism utilised in PCA catabolism. For the 2,3-ring cleavage pathway, the *pra* operon from *Paenibacillus sp. JJ1B* (AB505864) was utilised for cblaster searching. For the 4,5 pathway the *pmdEFDABC* (AB462808) operon was utilised for cblaster searching. For the 3,4 pathway no complete operon could be sourced, as such three exemplar MFS PcaK sequences were sourced from Uniprot for which significant experimental evidence existed: *Pseudomonas aeruginosa* (Q9I6Q3), *Acinetobacter baylyi* (*A. baylyi*) (Q43975) and *P. putida* (Q51955). After visualisation via iTOL, representative sequences based on distance from root were selected from around the tree to cover a taxonomic range of putative transporters to screen.

### General microbiology

*Escherichia coli* (*E. coli*) and *P. putida* were grown on standard unbuffered LB miller broth and agar (Formedium #LMM20L, #LMM0204) at 37 °C and 30 °C with 180RPM of shaking. When appropriate, antibiotics were added to an appropriate inhibitory concentration (100 µg/mL for *E. coli* and 500 µg/mL for *P. putida*).

### Strain construction

*E. coli* DH5⍺ was used for DNA cloning and assembly of all constructs. *P. putida* KT2440 was used in the characterisation of all PcaKs and TphKs through biosensor assays. *P. putida* KT2440 genomic knock-outs of *pcaK* and *fcs* were constructed using a sucrose counter selection method as described previously [52]. Briefly, electrocompetent cells were transformed with 1 µg of the relevant pk18mobsacB plasmid possessing upstream and downstream homologs regions flanking the *pcaK* gene, plated onto LB agar supplemented with 20 µg/mL of kanamycin and incubated at 30 °C. Individual colonies from the resulting plate were streaked onto YT agar supplemented with 25% sucrose (10 g/L yeast extract, 20 g/L tryptone, 250 g/L sucrose, 18 g/L agar) for counter selection. Colonies were screened with genomic primers to identify successful deletion of the *pcaK* or *fcs* gene. All chemicals used in substrate specificity screening can be found in Table S4-5, all chemicals were prepared in sterile water or DMSO as appropriate.

### Plasmid construction

All plasmids and primers used in this study can be found in Table S1-2, relevant genes encoding transporters including chimeric designs can be found in Table S3**.** The synthesis of all DNA oligonucleotide primers and gene fragments was performed by Integrated DNA Technologies (IDT). Amplification of plasmids or DNA fragments was performed using Q5 High fidelity polymerase (NEB, #M0491S). Assembly and ligation of DNA fragments for plasmid construction was performed using NEBuilder HiFi DNA Assembly Master Mix (NEB, #E2621S). Following assembly bacterial transformation was performed using chemically competent *E. coli* DH5⍺ cells, the resulting colonies were screened by colony PCR using Phire II Green polymerase master mix (Thermo Fisher, #F126S). Plasmid isolation was performed for positive colonies, and were used for assembly validation, by Sanger sequencing (Source Bioscience, United Kingdom).

### Biosensor based screening of MFS transporters

Overnight cultures of *P. putida* KT2440 or *P. putida* Δ*pcaK* harbouring a relevant plasmid were sub-cultured to an OD_600_ of 0.6 in 10 mL of LB media supplemented with 500 µg/ml carbenicillin before being aliquoted into 96 deep-well plates (DWP) prefilled with 50 µL of inducer (TPA or PCA respectively) to a final volume of 500 µL. The DWP were then transferred to a plate shaker incubator at 30–37 °C at 1000 rpm, 75% humidity. For PCA uptake assays, DWP cultures were incubated for 3 h. For TPA uptake assays, DWP cultures were incubated for 16 h. To measure OD_600_ and fluorescence, culture pellets were washed and resuspended in 500 µL in PBS before being transferred to 96 well clear bottom microplates (Greiner). OD was measured at 600 nm, GFP was measured with λEx/λEm of 488/520nm using a ClarioStar microplate reader (BMG). Fluorescence was later normalised against the measured OD_600_ for each well. Initial validation of transporter activity was performed via titration of the PcaK and TphK constructs using 0-1mM of TPA and 0-5mM of PCA as inducer plotting the dose response curve of normalised fluorescence with a variable slope hill function. PcaK and TphK constructs identified as functional in the primary screening assays were taken forwards for substrate specificity screening using concentrations of inducer that had been shown to elicit maximum response from the biosensor with either TPA (1mM) or PCA (5mM). All biosensor experimental data were processed as means of three biological replicates.

### Structure and helix modelling

CC-TOP [53] was used to generate an overlapping map of helical topology using protein sequences of functionally verified TphKs and PcaKs from which a transmembrane helical consensus was determined. This consensus alignment was imported into Jalview and the Alphafold [54] model for PcaK (Alphafold: Q51955) aligned to confirm assignment of the secondary structural element regions.

### Chimeric transporter-biosensor construction and screening

Using the topological alignments of the helical regions of the *P. putida* PcaK and *Rhodococcus pyridinivorans* (*R. pyridinivorans*) as a guide, gblocks encoding PcaK and TphK with the helical regions 1, 4, 7, and 10 were designed in silico and ordered via IDT. These were subsequently assembled into PcaV and TB4 biosensor vector backbones respectively via isothermal assembly. Individual helical substitutions were performed via 2 fragment isothermal assembly using primers designed to amplify the wildtype TphK or PcaK MFS in its cognate biosensor backbone extruding the target helical region. A second set of primers with featuring homologs overhangs to the opposing transporter were then used to amplify the specific helical region to be substituted, in order to generate a chimeric MFS with this process repeated for helices 1,4,7 and 10. Following isothermal assembly and sequencing, the chimeric MFS genes were then reamplified and cloned into the opposing biosensor backbone generating the full set of constructs for testing. Predicted structures of the chimeric transporters were generated using Alphafold and annotated in ChimeraX [55]. Relative fold changes were calculated in two steps after collection of relative fluorescence units per unit optical density (RFU/OD_600_) data beginning with normalisation via the division of the RFU/OD_600_ values for each ligand by the uninduced RFU/OD_600_ to generate a normalised fold change per ligand for each construct. Relative fold change was then calculated on a per ligand basis by the division of each mutant fold change (FC_mut_) by the fold change of the negative control (FC_NoMFS_). These values were used for the plotting of heatmap data. For detailed statistics of significant effects please refer to Additional File 1.

## Results

### Bioinformatic mining of putative TphK’s

We initially sought to obtain a diverse pool of putative candidate transporters for screening to capture the full breadth of structure activity relationships that may have evolved within specific taxonomic groupings. Inferring functionality of MFS transporters is complicated by the generally low sequence identity between family members, creating the possibility of dataset contamination with transporters of divergent function. By contrast, neighbouring genes within MFS-encoding operons can display strong sequence homology [56]. *tphK* and *pcaK* are both known to cluster into operons containing genes related to the catabolism of TPA and PCA respectively. We therefore employed a “guilty by association” methodology to enhance the fidelity of our homolog search using the genetic context (presence, organisation, and proximity) of TPA/PCA catabolic genes as an indicator of potential transport activity.

**Fig. 1:**
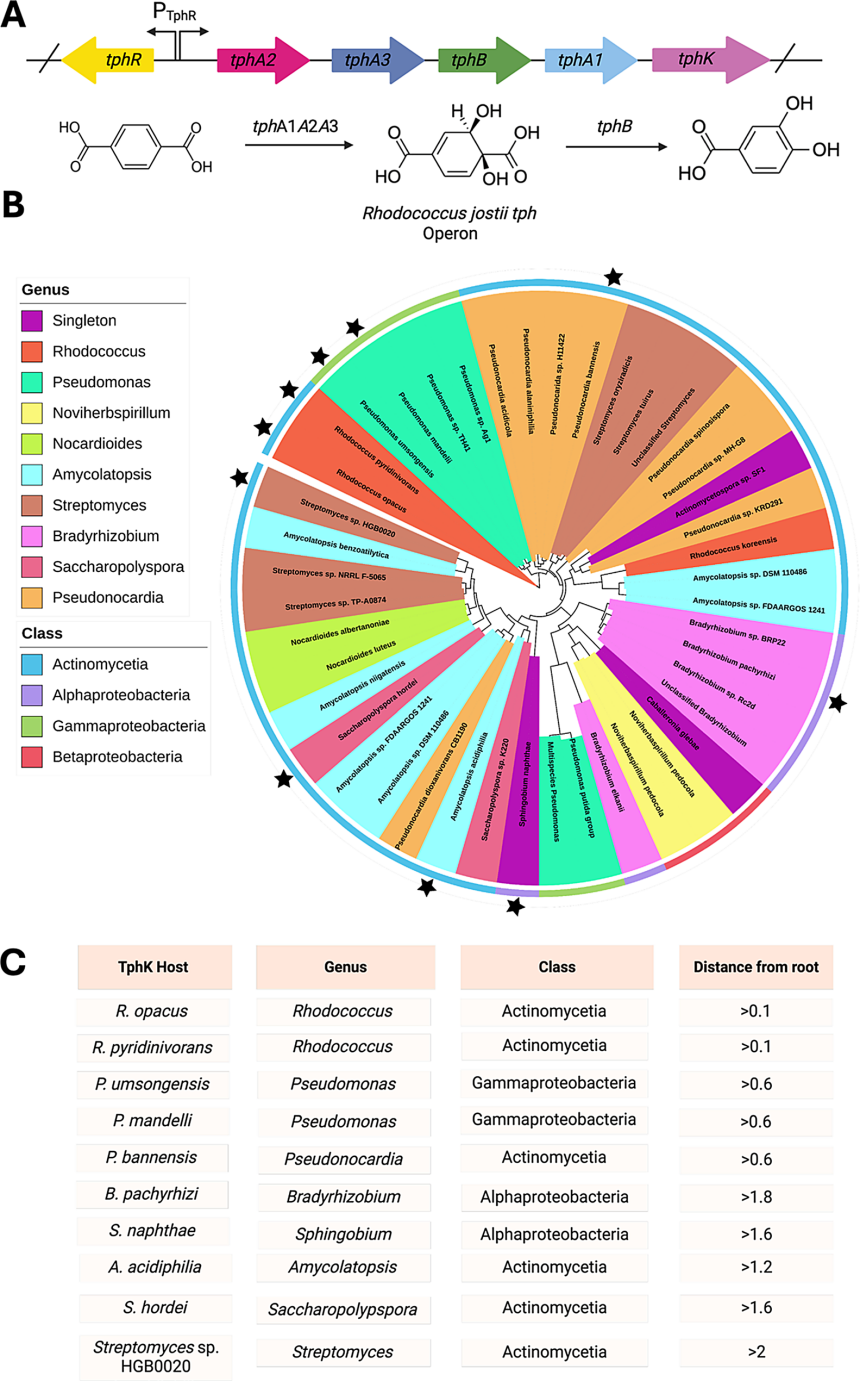
Phylogenetic and taxonomic analysis of the *tph* operon. (**A**) TPA degradation operon (*tphR* IclR-type transcription factor, *tphA2/3* TPA 1,2 dioxygenase large/small subunit, *tphB* 1,2-dihydroxy-3,5-cyclohexadiene-1,4-dicarboxylate dehydrogenase *tphA1* reductase component and *tphK* MFS) from *R. jostii* used in multigene BLAST search as a query, showing configuration of genes, and the catabolic transformation is also displayed at the bottom of the figure. (**B**) Phylogenetic tree of TphK homologs generated from the cblaster multigene BLAST tool analysis, rooted with respect to *R. jostii* TphK. Interior colour scheme designates genera, whilst the outer ring denotes the class of bacteria. Stars represent putative TphK’s carried forward for screening within the TPA responsive biosensor. (**C**) Selected TphK homologs including genus, class and distance from root, *R. jostii* whilst also screened forms the root of the tree and thus was excluded from the table

To achieve this, we queried the NCBI genomic database employing the cblaster multigene BLAST tool using the sequence of the entire TPA degradative operon from *R. jostii* (*tphRKA*_*2*_*A*_*3*_*BA*_*1*_) as the query [50]. The resulting phylogenetic tree displayed only 44 non-redundant leaves for MFS homolog co-located with the TPA catabolic operon (Fig. 1A). In contrast, a direct blastP homology search using the *R. jostii* TphK as the query resulted in a tree consisting of 530 leaves with some species that were not revealed in syntenic analysis (Supp Fig. 1A). Manual inspection of the results obtained from the direct BLAST search approach indicated that many of the MFS hits were not colocalised to TPA catabolic genes. For example, the MFS belonging to the genus *Janthinobacterium*, occurred 42 times in the direct blastP search yet demonstrated a genetic context indicating no relation to TPA catabolism (Supp Fig. 1B). Furthermore, no *Janthinobacterium* hits were present in the tree generated using cblaster, indicating that these MFS sequences did not carry any resemblance of TPA catabolic operon and were therefore unlikely to possess the desired functional activity.

The tree generated from syntenic analysis of the *R. jostii* TPA degradation operon was rooted with respect to the TphK transporter protein, with the TphKs generated from cblaster plotted using their relative sequence identity to the query (Fig. 1A). TPA catabolic operons typically differ based on the transporter type, with *Comamonas* sp. Strain E6 encoding a tripartite tricarboxylate transporter (TphC) and *R. jostii* encoding an MFS (TphK) [56]. To our knowledge, this is the first report of *tphK* containing TPA catabolic operons in the alpha and beta proteobacteria classes, indicating the spread of TPA catabolism through a much broader range of bacteria than previously thought. Actinomycetia is the predominant class of bacteria (66%) encoding TphK homologs, with tight clustering to the root of the tree with lengths of 0.1–0.6 indicating high similarity to the query sequence (Fig. 1B). Some Actinomycetia sequences however clustered further from the root at branch lengths of ≥ 1.6, indicating greater sequence diversity. The remaining classes consisted of alpha and Gammaproteobacteria (both 14%) as the next most abundant classes followed by betaproteobacteria (7%). Interestingly, the proteobacteria were noticeably less diverse at the genus level in comparison with Actinomycetia, with Gammaproteobacteria being represented solely by *Pseudomonads*, Alphaproteobacteria by *Bradyrhizobium* and Betaproteobacteria by *Noviherbaspirillum* and *Cabellronia*. We observed that the TphK sequences fell within 9 distinct bacterial genera with the bulk of representation within the tree originating from the *Pseudomonas* (*N* = 6), *Pseudonocardia* (*N* = 8), *Bradyrhizobium* (*N* = 5), *Streptomyces* (*N* = 6), *Amycolatopsis* (*N* = 7) and *Rhodococcus* (*N* = 3) genera. Notably the majority of species represented in the phylogenetic tree are saprophytic soil dwellers, with some originating from genera that have been previously validated as possessing the genes required for TPA catabolism, such as *Rhodococcus. Opacus* (*R. opacus*) and *P. umsongensis* but also encompass other previously unidentified soil dwellers like *Bradyrhizobium* and *Sphingobium*. This indicates the spread of the *tph* operon is more wildly spread than previously reported [56] however, the relatively limited distribution coupled with the high degree of sequence conservation likely reflects the fairly recent introduction of this xenobiotic carbon source and role of the *tph* operon as part of the accessory genome. Selected TphK candidates are indicated by stars in Fig. 1B and are listed in Fig. 1C, they included those from: *R. jostii*, *R. opacus*, *R. pyridinovorans*, *P. umsongensis*, *P. mandelli*, *Pseudonocardia bannensis* (*P. bannensis*), *Bradyrhizobium pachyrhizi* (*B. pachyrhizi*), *Sphingobium naphthae* (*S. naphthae*), *Amycolatopsis acidiphilia* (*A. acidiphilia*), *Saccharopolyspora hordei* (*S. hordei*), and *Streptomyces* sp. HGB0020 (*S.* sp. HGB0020).

### Bioinformatic mining of PcaK homologs

**Fig. 2:**
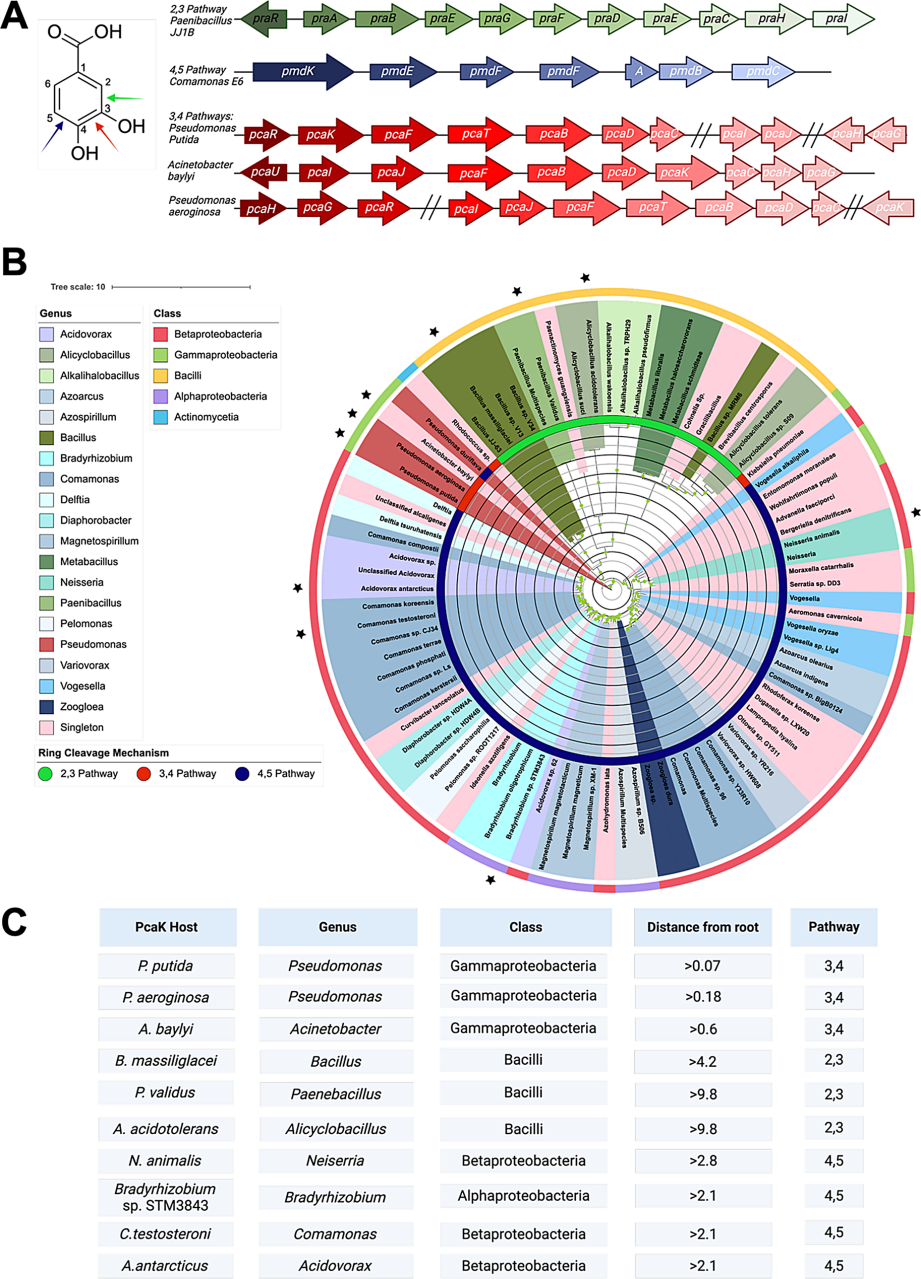
Phylogenetic analysis of PcaK transporters. (**A**) *pca* catabolic operons from *Paenibacillus* sp. JJ1B and *Comamonas* sp. E6, utilised for initial cblaster screening, plus three operons selected to represent the *3*,*4* pathway. (**B**) Phylogenetic tree of PcaK transporters rooted with respect to *P. putida* KT2440 PcaK (*3*,*4*). Inner ring colours denote the PCA degradation pathway of origin, outer ring stratifies by bacterial class of the transporter host organism. Leaves are coloured by host genus. Black and grey rings represent branch length increments of 1.4. Bootstrapping values are indicated by the size of green circles at root branches. Transporters selected for cloning are indicated with stars. (**C**) Summary of host, genus, class, distance from root and pathway of origin of PcaK homologs selected for further analysis

Following generation of a TphK phylogenetic tree we repeated the approach with the PCA catabolic operons. PCA catabolism is more widespread among bacteria than TPA catabolism, as it is a constituent of lignin degradation and a central node in diverse aromatic degradation pathways [28]. This is in contrast to TPA, which is of anthropogenic origin with only recent environmental exposure (1960-present) [28, 57]. Indeed, studies have highlighted at least three distinct bacterial pathways through which PCA is subsequently assimilated and incorporated into central metabolism [58–60]. These pathways can be categorised by their PCA cleavage mechanism, which occurs either in the *4*,*5* (*meta*), *3*,*4* (*ortho*) or *2*,*3* (*para*) position. While these pathways all serve to funnel PCA to central metabolism, they achieve this through distinct enzymatic steps and intermediate metabolites (Fig. 2A). Given this evolutionary divergence, we sought to explore the potential functional differences of PcaKs encoded in these three different pathways in our workflow. To this end, catabolic operons from strains encoding *2*,*3* and *4*,*5* extradiol pathways were selected from *Comamonas* sp. E6 (blue), and *Paenibacillus* sp. JJ1B (green) respectively as query sequences for cblaster (Fig. 2A). The *3*,*4* intradiol pathway, unlike the extradiol pathways, however, appears to be discontinuous, with the degree of fragmentation varying between the organisms that were investigated. For instance, in *P. putida*, the operon is split into three distinct loci encoding upper, middle and lower parts of the pathway separately. Conversely *Acinetobacter baylyi* shows no fragmentation, instead encoding its *3*,*4* degradative operons in a single continuous locus, however the syntenic arrangement differs to that of *P. putida* (Fig. 2A). As our operon synteny approach was incompatible with the degree of heterogeneity present in the *3*,*4* pathway, we instead incorporated PcaKs from experimentally verified strains known to possess the intradiol pathway into the phylogenetic analysis [11, 42, 61]. We elected to root the tree with respect the *P. putida* PcaK, as it had been previously experimentally verified and originated in the genome of the host strain *P. putida* KT2440 in which we intended to perform subsequent functional validation [25].

In total 88 PcaK sequences were retrieved from the three pathways 59, 3 and 26 from the *45*,* 3*,*4* and *2*,*3* pathways respectively (Fig. 2B). Bioinformatic mining of PcaK’s belonging to the *4*,*5*-pathway revealed that this pathway consists primarily of Betaproteobacteria (*N* = 45/85), with the largest subsection attributable to the *Comamonas* genus. This was followed by *Acidovorax* (*N* = 4) in addition to a number of single genera, representing the wide spread of the Betaproteobacteria class. A smaller number of Alphaproteobacteria were also identified, consisting of *Magnetospirillum* (*N* = 3) and *Bradyrhizobium* (*N* = 3), as well as some single instances of Gammaproteobacteria (*N* = 5). Despite the variation in class and genus, there was strong clustering of the *4*,*5* pathway as a single clade on the tree with a 2.8 average distance from the root, indicating high sequence homology between members of the *4*,*5* pathway. The exception to this is the PcaK from *P. duriflava*, which appeared to cluster with the *3*,*4* pathways.

The *2*,*3* pathway on the other hand was substantially smaller (*N* = 21) than the *4*,*5* dataset and was restricted to Bacilli. Furthermore, sequence variation was greater within this dataset with branch lengths from the root in the range of 4.2–11.9, indicating greater evolutionary diversity. In general, those belonging to the *Bacillus* genus demonstrated the closest sequence homology to the root, whilst other genera such as *Paenibacillus*,* Alicyclobacillus* and *Metabacillus* displayed branch lengths ~ 9 indicating significant sequence diversification from the root despite being closely related to the bacillus genera. Of the genera, *Bacillus* were the most abundant (*N* = 5) followed by *Alkalihalobacillus* and *Metabacillus* (*N* = 3 each), and finally *Paenibacillus* and *Alicyclobacillus* (*N* = 2). Similar to the *4*,*5* pathway, all members of the *2*,*3* pathway appear to share a common ancestor and cluster together, indicating greater sequence similarity to one another than to other pathways, despite the high sequence variation.

Finally of the PcaK homologs selected to represent the *3*,*4* pathway, those from *P. putida* PRS2000 and *P. aeruginosa* clustered very closely to the root with branch lengths of 0.07 and 0.18, respectively, followed by *A. baylyi* with 0.6. Interestingly, despite belonging to the *Pseudomonas* genus, *P. duriflava* possessed a branch length relative to the root of 2.1, indicating significantly more sequence dissimilarity and placing it closer the *4*,*5* pathway. In total ten transporters were selected from the *3*,*4* (*N* = 3) and *2*,*3* (*N* = 3) and *4*,*5* (*N* = 4) pathways based on distinct genera to ensure maximum diversity for subsequent transport and functional analysis in order to assess the potential functional differences of PcaK homologs. PcaK homologs selected for screening are listed in Fig. 2C, they include: *P. putida*, *P. aeruginosa*, *A. baylyi*, *Bacillus massiliglacei* (*B. massiliglacei*), *Panaebacillus validus* (*P. validus*), *Alicyclobacillus acidotolerans* (*A. acidotolerans*), *Neiserria animalis* (*N. animalis*), *Bradyrhizobium* sp. STM3843 (*B.* sp. STM3843), *Comamonas testeroni* (*C. testeroni*), and *Acidovorax antarcticus* (*A. antarcticus*) (Fig. 2C).

### Screening of putative MFS’ via responsive biosensors

**Fig. 3:**
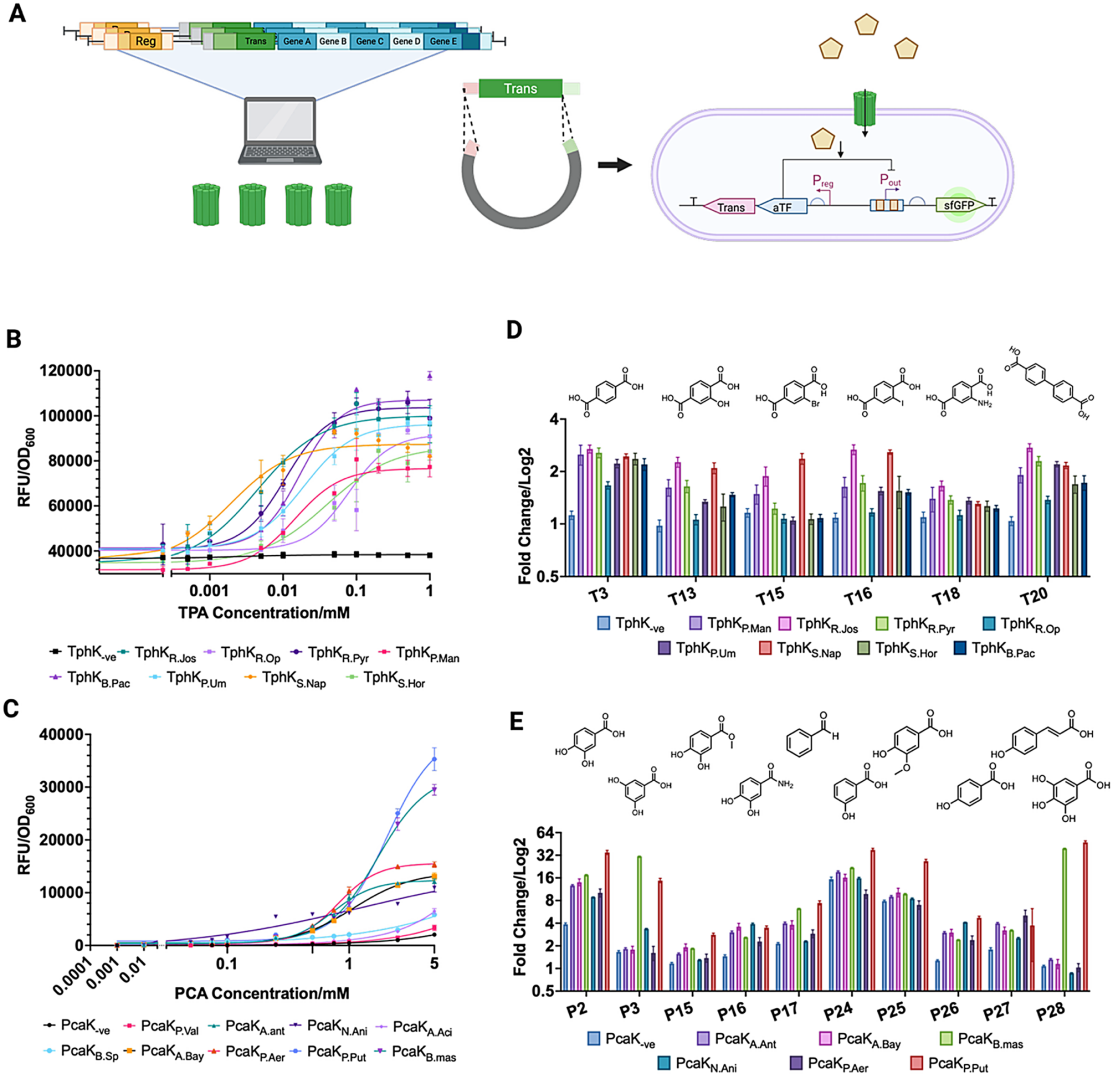
Biosensor based characterisation of TphK and PcaK homologs and structure activity relationship screening against effector ligand analogues. (**A**) Schematic representation of workflow beginning with the identification of homolog transporters followed by cloning into plasmids containing appropriate responsive allosteric transcription factors (aTFs) coupled to *sfgfp* expression to elucidate transporter functionality. (**B**) Dose response curve of TPA leading to expression of GFP normalised to cell density (RFU/OD600) for TphK homologs from 8 bacterial species. Each data point is representative of the mean and standard deviation of *n* = 3. (**C**) Dose response curve of PcaK homologs from 9 bacterial species normalised as for figure panel C. Each data point is representative of the mean and standard deviation of *n* = 3. (**D**) Significant TphK-TphR transporter-biosensor activation in response to TPA analogues in the *Δpcak P. putida* host. (**E**) Significant PcaK-PcaV transporter-biosensor activation in response to PCA analogues in the *Δpcak P. putida* host. Bars are representative of means of *n* = 3 biological replicates. 2-way Anova was used to validate statistically significant responses induced by effector analogues. Analogues which did not induce significant fold change for any transporter-biosensor construct are not plotted

Following the selection of homologs of TphK and PcaK transporters from the synteny-enriched phylogenetic analysis we next sought to validate their transport activity toward TPA and PCA, respectively. To accomplish this, the genes encoding the selected putative TphK and PcaK transporters were cloned into a plasmid containing either a TPA (TphR) or PCA (PcaV) responsive allosteric transcription factors along with the corresponding responsive promoters controlling the expression of *sfgfp* (Fig. 3A). The TphR biosensor was based on the IclR-type transcriptional activator from *Zhizhongheella caldifontis* [62], while the PcaV biosensor was based on the MarR family transcriptional repressor from *Streptomyces coelicolor* [63].

Under neutral pH conditions, both PCA and TPA will be deprotonated (singly and doubly), which will impede their cellular uptake via membrane permeation in the absence of a transporter [64]. However, natively, *P. putida* is capable of assimilating PCA via an endogenous *pcaK*. To mitigate endogenous transport, PcaK homologs were screened in a Δ*pcaK* genetic background, while TphK homologs were screened in the wild type *P. putida* genetic background. As such, any increase in GFP fluorescence observed by *P. putida* harbouring transporter-biosensor constructs, either TphK-TphR or PcaK-PcaV following exposure to TPA or PCA, can be attributed to the activity of the transporter homologs. To further validate this, transporter-less versions of both biosensing constructs (TphK_− ve_ and PcaK_− ve_) were developed to account for any passive transport through the membrane.

### Dose response characterisation of TphK-TphR biosensor constructs

The library of 11 TphK homolog biosensor (TphK-TphR) constructs was evaluated for the ability to induce GFP in the presence of an increasing TPA ligand concentration gradient (ranging from 0.005 to 1 mM), and the resulting dose response curve were compared (Fig. 3B). Parameters for functional biosensor constructs are given in Table 1, the biosensor constructs using MFS’s from *S.* sp. HGB0020, *A. acidiphilia* and *P. bannensis* were shown to be non-functional against TPA (data not shown). The TphK-biosensor constructs demonstrated similar operational sensing ranges (0.005–0.25mM) (Fig. 3B), but with considerable variation in activation (max RFU/OD_600_), sensitivity (EC_50_) and fold induction to TPA (Table 1).

**Table 1:** Extracted parameters of functional TphK_TphR biosensor constructs when challenged with increasing TPA concentration, including no transporter control (TphK_− ve_)

MFS origin	Basal RFU/OD_600_ (AU)	Max RFU/OD_600_ (AU)	EC_50_ (µM)	Fold Induction (AU)
TphK-ve	36,715	38,451	*	1.04
*R. jostii* (TphK_R.Jos_)	33,878	100,079	4.43	2.95
*R. opacus* (TphK_R.Op_)	40,318	91,854	79.17	2.27
*R. pyridinivorans* (TphK_R.Pyr_)	40,672	103,676	10.74	2.54
*P. mandelli* (TphK_P.Man_)	31,708	76,654	14.44	2.41
*B. pachyrhizi* (TphK_B.Pac_)	41,319	107,040	17.14	2.59
*P. umsongensis* (TphK_P.Um_)	40,788	96,370	19.08	2.36
*S. naphthae* (TphK_S.Nap_)	35,247	87,369	1.96	2.48
*S. hordei* (TphK_S.Hor_)	34,706	86,491	39.61	2.49

Homolog names used in figures are provided next to the corresponding species name in brackets

* Parameter not applicable

### Dose response characterisation of PcaK-PcaV biosensor constructs

Evaluation of the library of putative PCA transporter-biosensor (PcaK-PcaV) constructs was performed in a *P. putida* Δ*pcaK* strain. It was found that the Δ*pcaK* strain was still able to utilise PCA for growth, however it displayed an extended lag phase of 4 h (compared to 2 h for the wild-type strain), during which time no PCA was consumed in line with previous studies [25] (Supp Fig. 2A-C). As such, the PCA dose response assays were incubated for a maximum of 3 h to avoid endogenous utilisation of PCA. PcaK-PcaV transporter-biosensors constructs were tested against an increasing concentration of PCA (ranging from 0.01 to 5 mM), and the resulting dose response curves were compared (Fig. 3C) with the extracted response parameters exhibiting even greater variation in EC_50_, fold induction and RFU/OD_600_ than was seen in the TphK-TphR constructs (Table 2).

**Table 2:** Extracted parameters of functional PcaK-PcaV biosensor constructs when challenged with increasing PCA concentration, including no transporter (PcaK_− ve_) control

MFS origin	Basal RFU/OD_600_ (AU)	Max RFU/OD_600_ (AU)	EC_50_ (µM)	Fold Induction (AU)
PcaK-ve	963	3026	*	3.1
*A. antarcticans* (Pcak_A.Ant_)	1436	13,749	735.4	7.7
*A. baylyi* (PcaK_A.Bay_)	1587	15,227	1087	9.6
*B. massiliglaciei* (PcaK_B.Mas_)	2227	34,282	1706	15.4
*N. animalis* (PcaK_N.Ani_)	1624	14,802	847.4	9.1
*P. aeroginosa* (PcaK_A.Aer_)	1585	16,856	1706	10.6
*P. putida* (PcaK_P.Put_)	981	41,357	2051	42.2

Homolog names used in figures are provided next to the corresponding species name in brackets

* Parameter not applicable

Unlike with the TphK_− ve_- construct, the PcaK_− ve_- construct did demonstrate a small increase in biosensor activation when PCA was supplemented at concentrations above 1 mM (Fig. 3C). This could be due to the action of promiscuous transporters encoded in the host genome or passive diffusion across the membrane. PCA has a single pKa (4.2), whereas TPA possesses two pKa’s (3.5 and 4.3), so the latter is more acidic and will be doubly negatively charged, thus less likely to diffuse cross the bacterial membrane [65]. We compared the OD_600_ data at 5mM of PCA induction to ascertain whether there were any associated cytotoxic effects that may inhibit growth and effect the resulting curve parameters; however we found this to not be the case for either the negative control or PcaK_P.Put_ which exhibited the highest response to PCA (Supp Fig. 2D).

Biosensor constructs utilising MFS’s from PcaK_B.Mas_ and PcaK_P.Put_ demonstrated the greatest overall activity in terms of max signal and fold change, albeit with low sensitivity to PCA (Table 2). Interestingly this is despite the relatively large sequence difference between the two homologs (Fig. 2B), whilst this could be due to better expression of the PcaK_P.Put_ MFS in the host, the closely related PcaK_P.Aer_ construct showed much lower induction toward PCA (10.6) indicating that the effect may not be entirely due to better expression in the host and may indeed be as a result of the PcaK homolog. Other constructs displayed higher sensitivity to PCA (EC_50_ between 0.73 and 1.25 mM), with more modest induction toward PCA (7-10-fold). Finally, constructs PcaK_P.Val_, PcaK_B.STM_, and PcaK_A.Aci_ demonstrated the weakest response to PCA, with only marginal activation above the negative control (PcaK_− ve_), as such were assumed to be non-functional (Fig. 3C). The PcaK_C.Tes_ construct, surprisingly, was also inactive toward PCA (data not shown) despite the strain being reported to grow on 4HBA as a sole carbon source and possessing an intact *4*,*5* PCA catabolic operon [66]. At the end of screening 8 TphK homolog constructs: TphK_R.Jos_, TphK_R.Op_, TphK_R.Pyr_, TphK_P.Um_, TphK_P.Man_, TphK_B.Pac_, TphK_S.Nap_, TphK_S.Hor_, and 6 PcaK homolog constructs: PcaK_P.Put_, PcaK_P.Aer_, PcaK_A.Bay_, PcaK_B.Mas_, PcaK_N.Ani_, and PcaK_A.Ant_ were validated as functional via the biosensor assays and were taken forward for additional characterisation.

### Substrate dependent activation of TphK-TphR biosensor constructs

The variable activation of the different TphK-TphR and PcaK-PcaV biosensor constructs toward their cognate effectors alludes to variability in the substrate uptake capabilities of the different transporter homologs. Given the taxonomic diversity of their bacterial backgrounds, we reasoned that whilst the active transporters may transport the cognate ligands TPA and PCA, they may also possess extended substrate specificity. As such, we next sought to explore the structure activity relationships for each of the TphK and PcaK homolog biosensor constructs utilising a library of TPA and PCA effector analogues (Supp Tables 1 & 2).

To examine this, the functional TphK biosensor constructs (Table 1): TphK_R.Jos_, TphK_R.Pyr_, TphK_R.Op_, TphK_P.Man_, TphK_P.Um_, TphK_S.Hor_, TphK_S.Nap_ and TphK_B.Pac_ were screened against a panel of 27 TPA analogues at a concentration of 1 mM comparing fold-change activation of the biosensor relative to the negative control (TphK-ve) (Fig. 3D; Supp. Figs. 3A and 4A). Of those effectors screened, activity was observed towards compounds with substituents in the 2’ positions of the benzene ring, including a hydroxyl- (**T13**), bromo- (**T15**), iodo- (**T16**) and amino- (**T18**) at this position in addition to a TPA control (**T3**) (FC > 1.5). TphK_R.Jos_ displayed the greatest induction towards **T13** followed by TphK_S.Nap_ TphK_P.Man_, and TphK_R.Pyr_ indicating these homologs contribute positively to induction. For **T15**, TphK_S.Nap_ demonstrated the greatest induction, perhaps indicating a greater uptake of the brominated TPA analogue relative to the other screened TphK homologs. **T16** featuring a larger iodo group, generated strong induction from TphK_R.Jos_ and TphK_S.Nap_ with lower levels of induction for other all other TphK homologs apart from TphK_R.Op_ which did not significantly respond. Response to **T18** however was poor for all homologs screened with the best response achieved by TphK_R.Jos_.

Interestingly, we also observed activity towards biphenyl-4,4-dicarboxylic acid (**T20**) by all TphK biosensor constructs assessed (average FC ~ 2), a compound which is significantly longer than TPA. TphK_R.Jos_ and TphK_R. Pyr_ demonstrated the strongest response to **T20** suggesting a greater ability to accommodate the longer effector than TphK_S.Hor_, TphK_B.Pac_ and TphK_R.Op_ which showed lower levels of biosensor induction. Analysis of supernatants of wildtype *P. putida* and *P. putida* encoding the MFS on the TB4 biosensor plasmid demonstrated that no depletion of 4,4-biphenyldicarboxlic acid had taken place over the course of 24 h (Supp Fig. 5), indicating the response was generated directly from the effector and not due to intracellular conversion of 4,4-biphenyldicarboxylic acid to TPA.

TphK_R.Jos_ and TphK_S.Nap_ displayed the greatest effector range, inducing in the presence of six and five TPA analogues, respectively. Consistent with our previous observations of TPA biosensor induction (Fig. 3B), TphK_R.Op_ demonstrated the lowest response towards those effectors screened, only responding to TPA and biphenyl-4,4-dicarboxylic acid (FC = 1.4 and 1.2), whilst TphK_R.Jos_ showed the highest induction across the active effectors. It is however impossible to generalise overall trends in activity across the screened homolog constructs, as this is likely to be a combined effect of the transporter specificity and the biosensor [67] making it difficult to draw conclusions as to the sensitivity of one effector over another across the screened homologs.

### Substrate dependent activation of PcaK biosensor constructs

Functional PcaK-PcaV transporter-biosensors (Table 2), PcaK_A.Ant_, PcaK_A.Bay_, PcaK_B.Mas_, PcaK_N.Ani_, PcaK_P.Aer_ and PcaK_P.Put_ were screened against a panel of 28 PCA analogues at a concentration of 5 mM (Fig. 3E; Supp Figs. 3B and 4B). Ten analogues including PCA (**P2**) were found to generate biosensor induction for in the PcaK homolog biosensor constructs, with these effectors sharing structural similarities to PCA, such as hydroxylation at the 3 or 4 positions and the presence of a single carboxyl group. Examples of this include 3-hydroxybenzoic acid (3-HBA) (**P24**) and 4-HBA (**P25**). PcaK_P.Put_ generated the strongest induction towards **P24** of the screened PcaK-biosensor constructs with all constructs apart from PcaK_P.Aer_ achieving equivalent or greater induction than the negative control (PcaK_− ve_). Biosensor activation in response to **P25** was not statistically significant for all constructs when compared to negative control, with the exception of PcaK_P.Put_ (7.9 vs. 26.8-fold), potentially indicating the specific activity of the PcaK transporter from *P. putida* for **P25** uptake. (Fig. 3D; Supp Figs. 3B and 4B). This is consistent with other studies that have previously identified this transporter as responsible for the uptake of 4HBA [68], and may imply that the other PcaK homologs have reduced or no activity for this effector. Interestingly, the negative control also demonstrated significant induction toward both **P24** and **P25** (~ 15-fold vs. ~ 8-fold), indicating the potential involvement of another endogenous transporter providing redundant uptake of both effectors leading to significant induction of the negative control.

Interestingly, PcaK_B.Mas_ and PcaK_P.Put_ appeared to demonstrate unique activity for 5 hydroxyl substitutions, with high activation observed by 3,5 dihydroxybenzoic acid (**P3**) (30.9 and 14.8-fold, respectively) and gallic acid (**P28**) (39.2 and 47.6-fold, respectively). As other PcaK homlog-biosensor constructs demonstrated little to no response to either substrate this response appears to be uniquely linked to the expression of the MFS proteins from *P. putida* and *B. massiliglacei* and may indicate a preference of these transporters toward 5’ substituted effectors. Some of the PCA analogues also induced smaller, but still significant, responses relative to the negative control, including: methyl-3,4-dihydroxybenzoic acid (**P15**), to which PcaK_P.Put_ responded; 3,4 dihydroxybenzamide (**P16**), to which PcaK_A.Ant_, PcaK_A.Bay_, and PcaK_P.Put_ responded; and benzaldehyde (**P17**), to which PcaK_A.Ant_, PcaK_A.Bay_, PcaK_B.Mas_ and PcaK_P.Put_ responded (Fig. 3D). These analogues have differing carbonyl groups, suggesting some flexibility at this position at the expense of drastically reduced activation. To rule out potential oxidation of the benzaldehyde to benzoic acid additional screening of the active PcaK constructs was performed against benzoic acid (Supp Fig. 6), however no response was detected indicating activity was strictly toward the aldehyde functional group. Finally, partial activity towards, vanillic acid (**P26**) and coumaric acid (**P27**) was observed, by the screened PcaK biosensor constructs. **P26** and **P27** are natively metabolised by *P. putida*, with PCA being a pathway intermediate for both [28]. We reasoned that this activity may be a result of some degradation of coumaric and vanillic acid to PCA (**P2**) and/or 4-HBA (**P25**), resulting in indirect activation of the biosensor.

To investigate this, a Δ*fcs* (feruloyl-CoA synthetase) knock out strain was generated to abolish coumaric acid catabolism and subsequently transformed with the PcaK_p.put_-PcaV construct (Supp Fig. 7). No activation of this biosensor was observed in the Δ*fcs* knockout strain relative to the wildtype in the presence of coumaric acid except at high loading (5mM), indicating that biosensor activity observed in the WT strain was likely due to metabolism to PCA and/or 4-HBA. Despite this we observed increased activation relative to the negative control (~ 1.5-fold) indicating that the PcaK biosensor constructs appear to increase the response towards these two effectors.

Finally, evaluation of the raw RFU data in addition to the final OD_600_ value at measuring was also performed for the negative control (TphK_− ve_ and PcaK_− ve_), PcaK_P.Put_ and TphK_R.Jos_ to determine whether the assay concentration of aromatic acids was exerting cytotoxic effects that might create standardisation artifacts in our analysis. This analysis demonstrated that while some effectors did elicit a reduction in OD_600_, there was also a concomitant increase in RFU for PcaK_P.Put_ well above that of the negative control indicating that such responses were genuinely as a result of the activity of the transporter-biosensor constructs (Supp Fig. 8).

### Cross reactivity of TphK and PcaK biosensor constructs

Following effector screening of the TPA and PCA transporters paired with their cognate biosensors, we next sought to investigate the existence of overlapping activity between the TphK and PcaK transporters. As both TphK and PcaK are thought to belong to the aromatic H^+^ symport subfamily we reasoned that comparing their abilities to uptake TPA- and PCA-like effectors could highlight some structure activity relationships that govern ligand recognition or substrate specificity. To accomplish this, the genes encoding the TphK_R.Pyr_ and PcaK_P.Put_, MFSs were sub-cloned into the other biosensor backbone, generating the constructs TphK_R.Pyr_-PcaV and PcaK_P.Put_-TphR. These were then screened against the TPA and PCA analogue libraries.

The TphK construct appeared to demonstrate marginally better induction, than the negative control, towards ligands **P2**, **P24**, **P25** and **P27**, however no response to effectors other than what had been shown to generate activation of the biosensor in PcaK_P.put_ was observed (Supp. Figure 9 A). The PcaK crossover construct however demonstrated a significant induction toward 2,5-pyridine dicarboxylic acid (**T4**) indicating some unforeseen functionality, although the degree of fold change was minimal (~ 0.3-fold increase). Increased activity to 2-hydroxy TPA (**T13**) was also observed however all other effectors were shown to be unresponsive (Supp. Figure 9B).

### Biosensor mediated screening of PcaK and TphK point mutants

Next, we sought to evaluate the ability of both biosensor-based detection systems to discern changes in the activity following protein engineering of the MFS transporters. This was performed in order to examine the specific role of primary sequence elements and/or structural motifs in ligand recognition/transport. As an initial step, to verify that our biosensor assays would be able to accurately report a mutagenic phenotype for a transporter of interest, three amino acid point mutations, previously shown to affect the efficiency of **P25** uptake by PcaK from *P. putida* [68], were individually created. These residues at positions E144, R124, and R398 were replaced with alanine (A) in the PcaK_P.Put_-PcaV construct (Supp Fig. 10C), generating: PcaK-E144A_P.Put_-PcaV, PcaK_P.Put_-R124A-PcaV and PcaK_P.Put_-R398A-PcaV. These PcaK point mutant constructs were then evaluated against varying concentrations of both PCA and 4HBA to assess changes to dose response activity. Consistent with previous reports, the biosensor assay indicated that all three mutations led to near total abolition of induction in the presence of PCA import and a strong decrease in 4HBA induction (Supp Fig. 10A&B). The observed induction by 4HBA in both the negative control and point mutants is consistent with earlier effector screening, indicating endogenous redundant **P25** uptake mechanisms in the *P. putida* Δ*pcaK* strain.

### Sequence alignment and analysis of TphK and PcaK homologs

Based on the previously observed overlapping substrate specificity of TphK_R.Pyr_-PcaV and PcaK_P.Put_-TphK (Supp Fig. 9) we wished to further explore the sequence features that impart ligand specificity to TphK and PcaK. Given that the characteristic structural arrangement of MFS proteins consists of a classical 12 ⍺-helical bundle, we first generated a sequence alignment of the functional PcaK and TphK sequences using CC-TOP prediction to annotate the sequence alignment for the location of the 12 ⍺-helices (Supp Fig. 11). In addition, an Alphafold model of *P. putida* PcaK (Q51955) was also used to annotate the multiple sequence alignment, with its 3D structural prediction matching closely to the CCTOP predictions made for each sequence (Supp Fig. 11: Annotated as PcaK_PSEPU Secondary structure). The sequences of each individual transmembrane (TM) helix were then compared using the functionally verified TphK and PcaK sequences (Supp Fig. 12). Distinct conservation trends were noticeable in every TM, however appeared to occur with greater abundance in TM’s corresponding to those that make up the central cavity of the protein (TM’s: 1, 4, 7, and 10) with these TM’s thought to comprise the majority of residues that govern substrate coordination and co-substrate coupling [69]. Generally, amino acid differences in these regions consisted of non-polar to polar substitutions (TM1: PcaK_Ala22 to TphK_Gln, TM4: PcaK_Thr24 to TphK_Ala), polar to charged (TM10: Ser/Asn20 to Lys) and aromatic to non-aromatic (TM1: Phe11 to Gly). Within the PcaKs however, no sequence features were immediately apparent so as to explain the apparent unique uptake of 3, 5 substituted benzoic acids.

### Screening of core helical domain TphK_R.Pyr_ and PcaK_P.Put_ chimeric biosensor constructs

**Fig. 4:**
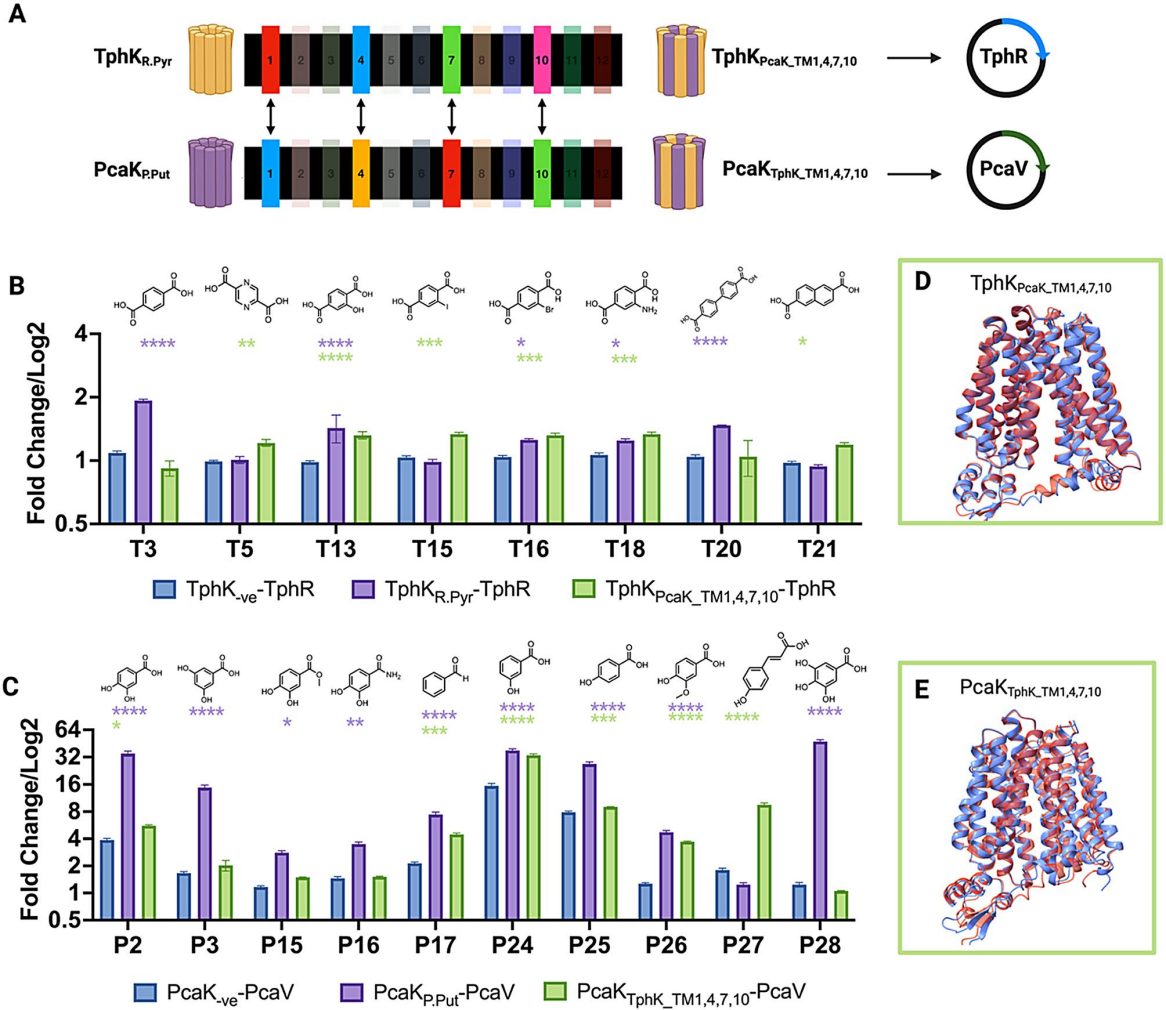
Structure activity relationship screening of PcaK and TphK core helical domain mutants in cognate biosensor backgrounds. (**A**) Workflow summary of the exchanging of α-helical domains 1, 4, 7 and 10 between TphK_R.Pyr_ and PcaK_P.Put_ generating chimeras TphK_PcaK_TM1,5,7,10_ and PcaK_TphK_TM1,4,7,10_ and cloning into the TphR and PcaV biosensor constructs for screening. (**B**) Plot of significant fold change induction by TPA analogues in TphK_− ve_ (blue) wild type TphK (lilac), or TphK_PcaK_TM1,4,7,10_ chimera (green) in the TphR biosensor background relative to the TphK_− ve_ control. (**C**) Plot of significant fold change induction by PCA analogues in PcaK_− ve_ (blue) wild type PcaK (lilac), or PcaK_TphK_TM1,4,7,10_ chimera (green) mutant in the PcaV biosensor background relative to the PcaK_− ve_ control. (**D**) A structural overlay of the predicted structures of the WT TphK (Red) and TphK_PcaKTM1,4,7,10_ chimera (blue) generated with Alphafold. (**E**) A structural overlay of the predicted structures of the WT PcaK (Red) and PcaK_PcaK_TM1,4,7,10_ chimera (blue) generated with Alphafold. All bars are fold change averages representative of 3 biological replicates calculated from raw RFU/OD_600_ values

Given the number of individual residue substitutions and combinations, both at the intra and interhelical level, that would need to be reconstituted in order to elucidate those involved in substrate recognition, we decided to leverage the high throughput screening potential of biosensors to assess the functionality of full TM substitutions between the *R. pyridinivorans* TphK and the *P. putida* PcaK for those helices which form the core of both, namely: TM’s 1, 4, 7 and 10. With this in mind, we generated and screened chimeric MFS transporters with the entire core TM helical regions exchanged, resulting in PcaK_TphK_TM1,4,7,10_-PcaV and TphK_PcaK_TM1,4,7,10_-TphR (Fig. 4A). These chimeric transporters were tested in their cognate biosensor backgrounds against the PCA and TPA analogue libraries (Fig. 4B & C; Supp Fig. 13).

Interestingly, whilst TphK_PcaK_TM1,4,710_-TphR appeared to lose activity toward its cognate effector, TPA and biphenyl-4,4-dicarboxylic acid (**T20**), it was still able to induce toward 2-hydroxy- (1.4 vs. 1.3), bromo- (1.0 vs. 1.3), iodo- (1.3 vs. 1.3) and amino-TPA (1.2 vs. 1.3) (TphK wildtype vs. TphK chimera), indicating some potential interactions from the PcaK helical regions in facilitating these inductions. Furthermore, a gain-of-function activity toward pyrazine-2,5-dicarboxylic acid (**T5**) and naphthalene-2,6-dicarboxylic acid (**T21**) was observed (Fig. 4B) indicating the potential cooperation of other TM helices contributing to increased uptake of these non-cognate effectors. The loss of function encountered appeared to specifically impact structures containing dicarboxylate functionality with no other functionalisation such as TPA (**T3**) and biphenyl-4,4-dicarboxylic acid (**T20**) suggesting that the core TM helices are responsible for this recognition in wildtype TphK.

The PcaK_TphK_TM1,4,7,10_-PcaV construct, appeared to be functional maintaining some activity toward earlier established effectors such as **P2** and **P25** albeit with reduced induction, indicating that the chimeric MFS was functional in vivo (Fig. 4C). In addition, this chimera displays a significant gain of function over the WT in the form of increased uptake of coumaric acid (**P27**) (9-fold). Activity toward PCA (**P2**), benzaldehyde (**P17**), 3-HBA (**P24)**, 4-HBA (**P25**) and vanillic acid (**P26**) were shown to be higher than the negative control indicating some retained ability to induce in the presence of these compounds yet not as efficiently as the wildtype PcaK (Fig. 4C). We also noted the apparent loss of induction towards substrates possessing 5th position hydroxylation such as 3,5 dihydroxybenzoic acid (**P3**) and gallic acid (**P28**) indicating that the core PcaK domains are essential for induction of the biosensor towards these substrates. We therefore then moved to study the chimeras as single exchange mutants to better elucidate regions of the highest importance for substrate recognition.

### Screening of individual helical domain chimeric TphK_R.Pyr_ and PcaK_P.Put_ biosensor constructs

**Fig. 5:**
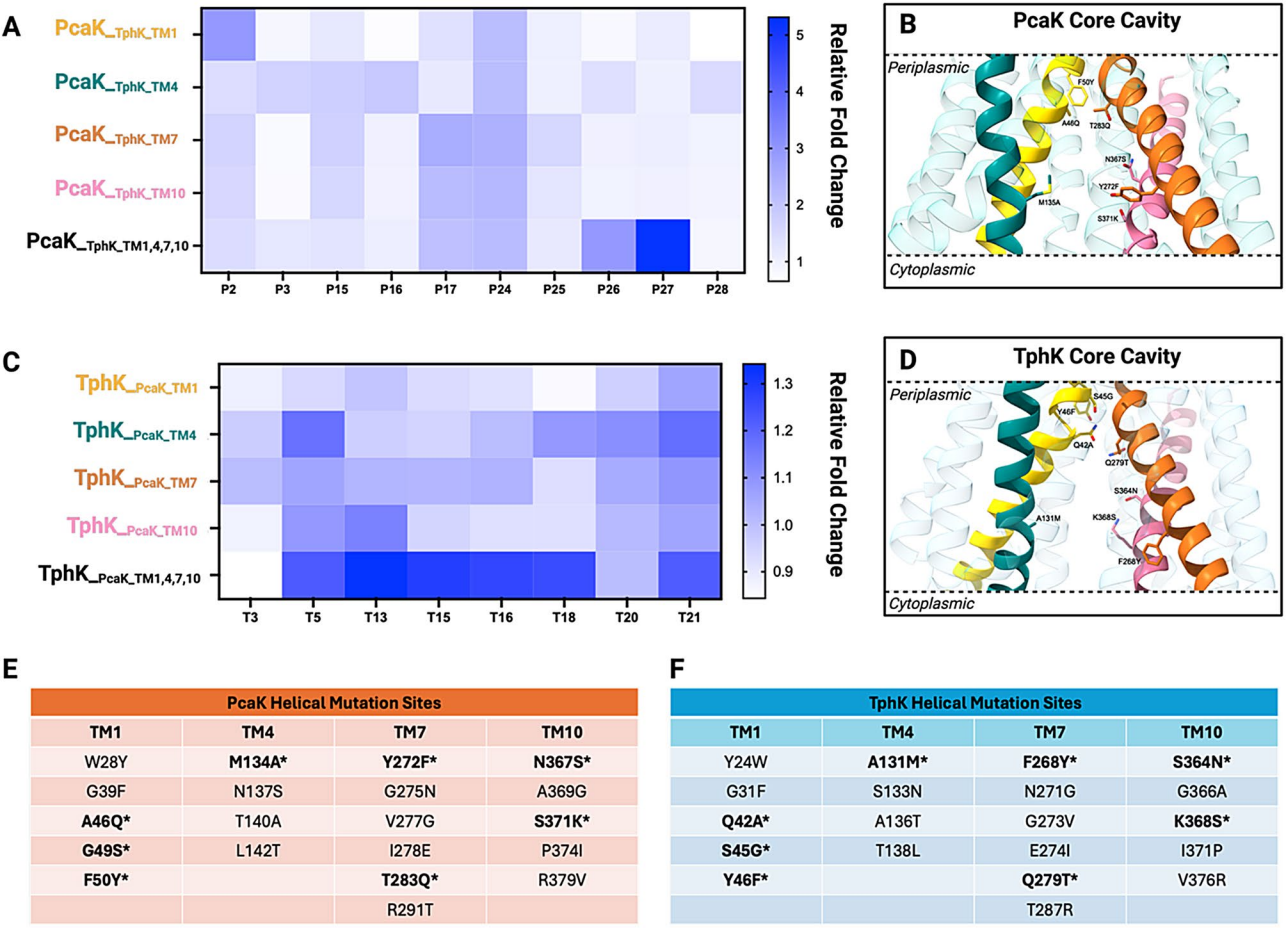
Assessment of single helical domain exchanges on portability of active substrates via relative fold change activation. (**A**) Heatmap displaying statistically significant increase or decrease in transport (GFP activation) of PcaK chimeras relative to the PcaK_− ve_ construct against selected effectors. (**B**) A predicted structure of the PcaK wildtype protein with the core helical domains exchanged during mutagenesis colour coded, conserved amino acids that were exchanged are shown as ball and stick representations with those pointing into the core cavity labelled. (**C**) Heatmap displaying statistically significant increase or decrease in transport (GFP activation) of TphK chimeric mutants relative to TphK_− ve_ constructs against selected effectors. (**D**) A predicted structure of the TphK wildtype protein with the core helical domains exchanged during mutagenesis colour coded, conserved amino acids that were exchanged are shown as ball and stick representations with those pointing into the core cavity labelled. (**E**) Mutations of conserved residues in PcaK introduced by exchange of core helical structures, with bold and starred mutations denoting those that occur in the solvent exposed core cavity of the protein. (**F**) Mutations of conserved residues in TphK introduced by exchange of core helical structures, with bold and starred mutations denoting those that occur in the core cavity of the protein. All predicted structures are shown in the inward open conformation with a side view of the core cavity

TM helices were swapped individually between TphK_R.Pyr_ and PcaK_P.Put_ (for a total of 15 chimeras) before being cloned into either the PcaV- or TphR-based biosensor background for the evaluation of gain or loss of function (Fig. 5).

### PcaK_P.Put_ chimeric biosensor effector screening

We first reconstituted the helical region swaps of the PcaK_TphK_TM1,4,7,10_-PcaV chimera individually to elucidate the effects of individual swaps (Fig. 5A). Surprisingly, activity towards the cognate ligand PCA (**P2**) appeared to be greater in the TM1 mutant than in the complete swap, indicating that substitution of this region with TphK TM 1 was able to partially complement and enable PCA induction. Consistent with this, the degree of amino acid changes within the TM 1 predicted structures for TphK and PcaK is subtle (Fig. 5E), with most of the solvent exposed core cavity facing mutations A46Q, G49S and F50Y clustering toward the periplasmic side of the core (Fig. 5B). These mutations all introduced slightly larger and more polar residues into the core cavity. This had the effect of reducing activity towards PCA, relative to the parental transporter, whereas induction activity to other effectors appeared to be more significantly reduced, indicating that decreasing the size of the cavity may have negatively impacted the ability to recognise PCA analogues.

Replacement of TM4 appeared to be generally well tolerated, permitting limited induction towards 5’ substituted effectors, both 3,5 dihydroxybenzoic acid (**P3**) and gallic acid (**P28**), a phenotype that was abolished in all other single helical exchanges (Fig. 5A), suggesting that the residues for 5’ substituted hydroxyl recognition may be located in the other TM domains. Based on the predicted positions of residues side chains of TM 4, the most solvent accessible mutation in the core cavity consisted of a M134A substitution, which lead to a loss of activity towards PCA, as such this methionine may play a role in stabilising interactions with aromatic residues. Overall, the generally lower impact of the TM 4 swap may be attributable to this helix having the fewest number of core cavity amino acid changes (Fig. 5E & F).

TM’s 7 and 10 demonstrate largely similar phenotypes to one another, appearing more strongly induce towards benzaldehyde (**P17**) and 4-hydroxybenzoic acid (**P25**) relative to TM 1 and 4 swaps. Initial inspection of the mutations of TM 7 and the corresponding predicted structure suggests that Y272F would reduce H-bonding capacity from the core cavity. However, the mutation of T283Q whilst bulkier, maintains this H-bonding capacity on the same face of the core cavity. Collectively, Y272 and T283, along with the conserved N367 (TM10) and S371 (TM10) residues may have direct effects upon effector binding, with these residues clustering around helical turns 3–4, consistent with studies on the XylE MFS transporter which demonstrates similar positioning of its ligand coordinating residues [70] (Fig. 5B). These residues are mutated in the TM 10 to N367S and S371K, with the latter lysine residue carrying a positive charge in addition to protruding further into the core cavity (Fig. 5B). The increased steric hinderance coupled with altered hydrogen bonding potential in TM 7 and 10 may therefore contribute towards the inability to accommodate 5’ hydroxylated effectors such as **P3** and **P28**.

Replacement of every individual TM, along with the full cross, led to activation with 3-hydroxybenzoic acid (**P24**) perhaps suggesting that none of the tested TM’s are directly responsible for the recognition of this effector. Notably, vanillic acid (**P26**) and coumaric acid (**P27**) induction was improved in the complete chimeric mutant PcaK__TphK_TM1,4,7,10_ however, individually reconstituting the single TMs showed very little improvement. Given the general trend of exchanging the bulkier residues of the PcaK core helical domains for the smaller ones of TphK domains may suggest that the increased cavity size of the TphK core is responsible for permitting the import of bulkier substrates such as coumaric and vanillic acid (Fig. 5A).

### TphK_R.Pyr_ chimeric biosensor effector screening

The exchange of TM 1 in the TphK mutant appeared to have the most severe impact on effector recognition including TPA (**T3**) (Fig. 5C). Despite this, most of the mutations that occur are predicted to be structurally located at the periplasmic and cytoplasmic interfaces of the protein and not in the centre of the core cavity; specifically, the mutations Q42A, S45G and Y46F, all result in the loss of hydrogen bond donor/acceptors potentially disrupting interhelical contact points preventing the protein from undergoing conformational changes required for function (Fig. 5D). The number of conserved amino acids targeted at this structural location may therefore provide explanation for the sharp reduction in effector recognition.

In contrast, replacement of TM 4 appeared to indicate this TM was the primary driver of the previously observed activity towards pyrazine-2,5-dicarboxylic acid (**T5**), and naphthalene dicarboxylic acid (**T21**) (Fig. 4A **&** Fig. 5C). Mutations in this TM were quite minor as mentioned earlier, with the major change being the A131M mutation leading to the incorporation of a methionine side chain pointing into the core, as shown in the predicted PcaK structure (Fig. 5B). This mutation may stabilise effector binding through an S-aromatic interaction with nearby helical residues enabling the non-cognate large aromatic effectors to be up-taken.

The TM 7 mutant displayed good response towards most effectors apart from 2-amino terephthalic acid (**T18**). Of the mutations occurring in the TM 7 helix, only the Q279T mutation was localised to the central core, with this mutation resulting in the loss of a potential hydrogen bond acceptor site, it is possible that this correlates the loss of activity toward 2-amino terephthalic acid (Fig. 5D). The reduced activity for **T18** could therefore be a result of this change, with bulkier substitutions like hydroxyl or halide groups still able to hydrogen bond with the shorter threonine residue at this position. Mutation of TM 10 appeared to have a greater impact on effector uptake, losing activity for iodo, bromo and amino terephthalate (**T15**,** T16**,** T18**) yet possessing the strongest induction towards hydroxy terephthalate (**T13**) of all the single TM mutants (Fig. 5C). Mutation of serine to asparagine (S354N) may explain the observed impact upon effector activity as asparagine is bulkier than serine this mutation may not accommodate bulkier substrates such as iodo and bromo terephthalates explaining the reduced response. More interestingly however, a conserved lysine residue is mutated to a serine (K268S) with the predicted structure of TphK showing this residue projecting up towards S364 (Fig. 5D). The positive charge provided by the lysine may be essential for the stabilisation of the additional carboxylate group of TPA like moules, with mutation to serine abolishing this. Given the proximity of the residues however, they may act synergistically to coordinate potential effector binding.lec

Interestingly, activity toward 2’ position substituted TPA homologs (**T13-18**) appears to be significantly enhanced when all the PcaK core helices are present together, with individual helical substitution not reaching the same levels of biosensor activation. Replacement of any of the core TM helices of TphK apparently led to a loss of function toward TPA and biphenyl-4,4-dicarboxylic acid indicating the importance of these core helices in substrate recognition. This result likely suggests the PcaK TM regions can substitute the loss of the second carboxylate recognition site through the ability to recognise hydroxyl or halide substituents on the ring providing some explanation for the observed induction of the biosensors in these mutants. Consolidating results from both the full cross chimeras and single helical domain mutations in combination with their predicted structures implies that PcaK TM helices can serve as functional proxy to TphK components but only when all are substituted together, suggesting some inter-helical dependency for functionality.

We then moved to assess the functionality of the same TphK chimeras when transferred into the PcaV biosensor background to screen for any gain of function toward PCA or its analogues (Supp Fig. 14A). Substitution of single PcaK helical regions into TphK appeared to have very little effect on the recognition of PCA like effectors, with the primary positive effect restricted to coumaric acid (**P27**). This further corroborates the observed improvement in activity when the PcaK full cross was assayed against coumaric acid and suggests involvement of other TphK TM regions in its recognition also. Finally, we assessed the activity of PcaK_TphK_ single helical exchange chimeras in the TphR biosensor background, except for PcaK_TphK_TM1_-TphR which failed to clone (Supp Fig. 14B). No activity to any the TPA analogues screened was detected with any of the TM exchange chimeras however, this result is consistent with the initial crossover experiments performed analysing the wildtype PcaK in the TphR biosensor background which also showed no response to any TPA like effectors. As such we can conclude that single replacement of PcaK TM structures with those from a TphK do not confer any activity to this effector set. Collectively, the results indicate that we have successfully generated a set of chimeric MFS biosensor constructs that are functional, with some demonstrating novel gains of function, highlighting the utility of such biosensor-based approaches to membrane transporter characterisation and engineering.

## Discussion

Here we demonstrate that the development of a high throughput biosensor enabled method for the screening and characterisation of MFS transporters is an important step towards the implementation of these and other transporters into strain engineering workflows. As such, screening strategies that take advantage of the rapidly expanding library of allosteric transcription factors as genetically encoded biosensors could pave the way for greater understanding of transport mechanisms, enabling deployment in microbial cell factories for bio-based production, and facilitate membrane protein engineering efforts. In this study, the aromatic acid, PCA and TPA, responsive transcriptional biosensors were employed to screen and characterise putative PCA and TPA transporter homologs belonging to the MFS family, in the industrially relevant host, *P. putida*. Future work, incorporating the TphK and PcaK homologs validated in this study into hosts that assimilate TPA and PCA would be useful to demonstrate whether the observed differences in biosensor enabled assays translate to real improvements strain fitness/productivity. Specifically, the range of sensitivities to TPA that were demonstrated in the TphK homologs could be leveraged in the selection of highly sensitive MFS transporters that are able to scavenge trace amounts of TPA from environments more efficiently.

### Bioinformatic mining of TphK and PcaK homologs

Using the genetic context of transporter genomic loci has been implemented previously to aid in the elucidation of a novel citric acid export protein in *Aspergillus niger* based on its homology to an itaconic acid biosynthetic gene cluster [71]. Furthermore, similar methodologies have been applied specifically to the TPA catabolic operon to systematically search for such metabolism [56]. Applying such syntenic catabolic operon analysis paired with biosensor-based functional screening led to the identification of three novel PcaK’s from *A. antarcticans*,* B. massiliglacei* and *N. animalis*, and four novel TphK’s from *B. pachyrhizi*,* R. pyridinivorans*,* S. hordei* and *S. naphthae*. To the best of our knowledge these MFS transporters have never been characterised or reported previously. From syntenic analysis, the taxonomic distribution of TphK containing operons appears to be broader than previously thought with the activity of the transporters providing evidence for the existence of TPA catabolic operons in these genera. Analysis from Jimenez et al. [56] used homology of the *tph*A2 gene to extract TPA catabolic operons from publicly available sequence databases, they attributed operon occurrences to a limited number of organisms including betaproteobacteria (*Comamonas*,* Ideonella* and *Ramlibacter*), gammaproteobacteria (*Pseudomonas*) and actinomycetes (*Rhodococcus*) with all betaproteobaceria utilising the TphC transporter. Our analysis demonstrates that genes encoding TphK may have a greater taxonomic spread than previously reported, as they appear in a greater number of species than first assumed as well as in a greater number of families, this may be due to the use of the entire operon including the *tphK* gene as a search query returning a more diverse selection of organisms. Interestingly we identified some betaproteobacteria that appeared to possess TphK encoding genes implying that *tph* operons in this class are not limited to only to those utilising TphC transport.

The aforementioned TPA catabolic operon from *Comamonas* sp. Strain E6 encodes a tripartite tricarboxylate transporter instead of a TphK MFS [57, 72] suggesting the possibility that two distinct transporter systems were acquired by the ancestral operon to enable TPA uptake. A possible explanation for the emergence of two transport systems with overlapping function could relate to the relative transport turnover of each system. Whilst both TphK and TphC are classified as secondary transporters, the latter relies on a solute binding protein which typically bind with high affinity for the substrate leading to its translocation across the membrane [73]. MFS proteins are known for their higher uptake rates of substrates however with generally lower affinity and specificity, whilst no direct comparison of uptake rates between these classes have been made the general trends of mechanism lend themselves to this concept [74, 75]. As such the selection of transport system may relate to the preference of the host for TPA as a primary carbon source for growth or as an ancillary carbon source with the transporter systems representing different ecological niches in TPA rich environments. Whilst syntenic operon analysis is effective, this bioinformatics mining method is highly reliant on the co-localization of transporters to other genes in a functional context, making it inappropriate in the cases of orphan MFS proteins or exporters which are often located at random in genomes; making functional appraisal challenging [76].

### Screening of TphK and PcaK biosensor homologs

Biosensor-enabled screening of the seven functional PcaK homologs revealed wide ranging sensitivity towards PCA, along with variable effector specificity for each PcaK construct, with some responding to analogues possessing hydroxyl groups at positions 3 and 4 of the aromatic ring, and carboxylate, methyl ester-, amide- and aldehyde groups at position 1. Furthermore, PcaK_P.Put_ and PcaK_B.Mas_ demonstrated unique ability for the uptake 3,5-dihydroxybenzoic acid and gallic acid, indicating an activity towards position 5 hydroxyl groups which is not shared by the other screened PcaK biosensor constructs. This indicates that the effector scope for the PcaV transcription factor is broader than previously reported [63].

The effector range of the of TphK containing biosensor constructs was also tested, with the levels of induction varying considerably for many of the effectors trialled on a per biosensor construct basis. Responses to para-substituted aromatic dicarboxylic acids, with a tolerance for polar substitutions in the 2nd ring position, such as: hydroxyl, nitro, amino and halogen groups were detected with some of the homologs demonstrating increased affinity for specific effectors such as TphK_S.Nap_ which displayed the strongest induction towards brominated TPA over the other screened homologs. Unexpectedly, they also displayed the ability to respond to biphenyl-4,4-dicarboxylic acid, an effector twice the length of the cognate effector TPA [57]. We have previously characterised the tripartite tricarboxylate transporter, TphC, from the TPA catabolic operon of *Comamonas* sp. strain E6. In that study we explored ligand recognition by the solute binding protein of TphC, which was able to bind TPA (**T3**), 2-hydroxy TPA (**T13**), 2-amino TPA (**T18**) and also biphenyl-4,4,-dicarboxylic acid (**T20**) [57]. The substrate specificity of TphC appears to overlap with the effector compounds that lead to induction in the TphK biosensor constructs characterised in this work, intuitively suggesting that as both TphK- and TphC-type *tph* catabolic operons possess the same core activity, the transporters therefore, may have overlapping substrate recognition. However, the overlapping uptake of biphenyl-4,4-dicarboyxlic acid does raise questions of the functional origin of both TphK and TphC, whether these originated as bona fide transporters of TPA or perhaps related to transporters from biphenyl catabolic pathways, another anthropogenic carbon source. In addition, the identification of this broadened substate scope also indicates that the TphR transcription factor from *Z. Caldifontis* is activated by aromatic diacids with increased length; thus the main determining factor for TphR activity appears to be the requirement to maintain diacids in a co-planar arrangement [63].

### Chimeric MFS biosensor construct screening

Currently, there are no reported crystal structures for any member of the aromatic acid H^+^ symporter family. As such, information on the residues and/or structural features pertaining to ligand recognition and transport is minimal. Relying on primary sequence alignment of the transporters alone does not always guarantee functional relevance, with regions of functional similarity (substrate binding or ion binding sites) often display poor sequence alignment. In more distantly related transporters, residues with a similar functional role are often located far from each other in a primary sequence alignment [77]. Analysis by Wada et al. hypothesised a possible ligand binding site in PcaK based on structural modelling using other MFS structures with some of these residues located on the core TM helices used in this study [25]. Thus, we opted to perform replacement of the core TM helices between TphK_R.Pyr_ and PcaK_P.Put_ to gauge which of these structural features may contain sequence elements pertinent to substrate recognition. A similar TM swap approach has been used before with the glucose transporters Hxt1 and 2, which enabled the identification of TM’s: 1, 5, 7 and 8 as being essential for glucose recognition and uptake [78]. Combination of rapid biosensor-enabled screening in tandem with predicted structure and sequence alignments suggests the involvement of a number of residues present in the core cavity which could be essential for effector recognition.

A fully combinatorial approach to swapping TM helical domains would have been ideal to fully probe the interactions between transporters, however as this study was intended to be a proof of principle, we instead decided to focus only on the core helices as combined or single swaps. In future studies generating intermediate constructs with double or triple helical exchanges will be valuable in deepening understanding of the helical interdependency and additive effects on effector response. All chimeras screened in their cognate biosensor background (Figs. 4 and 5) appeared to demonstrate functional transport albeit with some loss of activity relative to the wildtype. Gains of function were observed for the TphK_PcaK_TM1,4,7,10_ chimera toward 2,5 pyrazine dicarboxylic acid (**T5**) and naphthalene 2,6 dicarboxylic acid (**T21**) (Fig. 4B), indicating a deviation away from the established preference for the general TPA structure exhibited by the transporters characterised in this study. Given the retained recognition of 2’ substituted TPA analogues it is plausible that introduction of PcaK helices enabled recognition of more highly decorated structures via introduction of more hydrogen bonding residues into the cavity. Similarly, incorporation of TphK core TM helices into a PcaK scaffold led to significant increases in activity for coumaric acid (**P27**) a much bulkier effector than other PCA analogues validated in our earlier characterisation experiments, suggesting the both the TphK and PcaK cores have structurally adapted to the dimensions of the effectors they transport with TphKs featuring sterically less bulky side chains relative to PcaKs. Studies that have crystallised MFS proteins in complex with their substrate have shown a network of residues across multiple helices act in a coordinated manner to bind substrates. For example, the bacterial MFS XylE in complex with D-xylose is coordinated by a total of eight hydrogen bonds, via polar residues on TM’s 5, 7, 8 and 10 as well as by aromatic residues in the vicinity from TM1, 7 10 and 11 [70]. Interestingly, whilst Q288, Q289, and N294, which are all located on the solvent exposed helical turns 3–4 of TM 7 of XylE, contribute to hydrogen bonding with D-xylose; Y298 located at the helical turn 6 is also involved in ligand binding through water mediated hydrogen bonding [70]. Therefore, it is possible that some of the more distal conserved residues identified in our analysis such as T283 (PcaK) or Q279 (TphK) that underwent mutation in the TM swap may act in a similar manner.

Similarly, it is more difficult to ascertain the effect of other mutations introduced during helix exchanges, which do not occupy the core cavity and instead likely function as contact points for interhelical interactions. It is plausible that many of the effects we observed in the screened mutants were as a result of indirect disruption of protein structural dynamics, as such future studies that focus on targeted mutations of these implicated residues may shed more light on their role in proper protein function. We also noted the apparent synergism that occurs when multiple TM structures are exchanged simultaneously rather than individually. Key amino acid residues involved in selective protonation in response to effector binding have been inextricably linked to substrate translocation with the ionic motive force driving reconfiguration of MFS transporters from outward to inward conformations [79]. Given our observations, it is plausible that exchanging of TM helices may disrupt titratable amino acid residues responsible for the relaying of protons following substrate binding; the effect of mutating such charge carrying residues has resulted in the conversion of MFS proteins from active to passive transporter, drastically reducing uptake rate through the decoupling of the proton motive force.

Indeed, in the case of XylE, mutation of D27 abolished transporter function entirely whilst mutating R133, which stabilises D27, lead to considerably reduced transport function highlighting the importance of such titratable residues in MFS function [80]. We noted the presence of a conserved glutamate residue (E274) located in TM7 mutated to an isoleucine in PcaK which may represent such a titratable residue that was lost during mutation. Such results indicate the feasibility of performing not only protein engineering efforts with MFS transporters, but also to probe more fundamental questions as to the role of specific residues or structural motifs in transporter function, enabled with biosensors, to provide a sensitive means of detecting gains and losses of function. Future studies using more targeted approaches such as site directed mutagenesis or alanine scanning of core cavity amino acids may provide a facile means of building structure activity relationships for putative or unclassified MFS proteins and can enable rapid identification of critical residues for further investigation. Incorporating multiple biosensors in serial genetic circuits coupled to different fluorescent outputs could potentially allow for further multiplexing of the approach covering a much broader range of compounds permitting deeper characterisation of transporters and overcoming the substrate limitations of a single aTF [81].

### Limitations of proposed methodology

Whilst the results of the study are positive the disentanglement of biosensor and transporter activity is challenging. The PcaK from *A. baylyi* has been previously purified and reconstituted in a proteoliposome, where it was reported to be able to take up salicylate, 2,4-dihydroxybenzoic, 4HBA and PCA in addition to vanillic acid and 3HBA [42]. Further, PcaKs from *Acinetobacter* sp. and *Sphingobium* sp. were also reported to be able to take up PCA, 4HBA, 3HBA, and to some extent vanillic acid, through genomic deletion and growth assays [10, 42]. In this study, activation of the PcaK_A.Bay_ biosensor construct by salicylate (2,4-dihydroxybenzoic acid) was not detected by any of the biosensor-mediated transporter assays. Indeed, salicylate did not induce activation of the biosensor in any of the PcaK constructs screened. Presumably this is due to the inability of the PcaV biosensor to recognise these substrates, as this PcaK homolog has been shown directly to uptake salicylate. This highlights a limitation of the proposed method, whereby the assay depends on both transporter and biosensor specificity, a recognised challenge in biosensor development [67]. This could be remedied through the engineering of sensors to increase their substrate scope or through the incorporation of other allosteric transcription factors to further improve substrate breadth [63].

Further, we encountered background uptake of PCA, 4-HBA and 3-HBA in *P. putida* despite the deletion of endogenous *pcaK*. Previous work by Wada et al. has demonstrated that PCA uptake is facilitated by 3 other MFS transporters in addition to PcaK, with these also demonstrating some overlapping ability to import each-others cognate substrates [25]. This redundancy can make the interpretation of fold change highly challenging to completely isolate but can be accounted for via genomic disruption of the host strain to remove said transporters and through the use of biosensor only controls which we have shown can be used to interpret net effects on fold induction created by the presence of a functional MFS. Despite this, in future studies, using transporter minimised variants of strains would be enable much cleaner interpretation of assay data, although this would be significantly labour intensive. Variable expression of the different MFS transporters also represents a potential confounding factor in the analysis of induction as some transporters may express more poorly than others, negatively impacting their induction performance in a biosensor construct. Such a case may be plausible for TphK_R.Op_ which despite high sequence similarity to other TphKs from the Rhodococcus genus showed reduced sensitivity (Table 1; Fig. 3B); with reporter fusion or western blot experiments providing a means of accounting for differential expression of the MFS homologs.

Finally, the dynamics of the biosensors employed may be affected by the dynamics of the transporter itself. The entanglement of these two features is well documented, with Shin et al. demonstrating the optimisation of expression of an MFS was essential for the development of a reliable adipic acid biosensor [67]. Thus, whilst comparison between different homologs can be made for the same effector it is difficult to generalise the preference for one effector over another across all homologs as it is impossible to isolate the induction effect as a feature of the biosensor or the transporter.

## Conclusion

We report a novel aTF-biosensor based screening method for the identification and characterisation of MFS transporters. Bioinformatically mined PcaK and TphK homolog protein sequences for transporters of aromatic acids, PCA and TPA, were combined with TPA and PCA responsive biosensors. This led to the identification of novel homologs of both classes of aromatic acid H^+^ symporters. Syntenic analysis was employed to obtain an enriched set of PcaK and TphK homologs for screening. We report wider taxonomic spread of the *tph* catabolic operon than previously reported as well as the first studies functionally characterising TphK substrate scope. Homologs encompassing a broad phylogenetic scope were selected and cloned into TPA and PCA responsive biosensor constructs to determine functionality and effector scope. The effector response profiles of the biosensor-transporter constructs were examined, revealing novel functionality for PcaK-biosensors constructed from MFS proteins from *P. putida* and *B. massiliglacei*, which induced in the presence of 5’ hydroxylated PCA analogues relative to other tested constructs. In contrast the TphK constructs demonstrated varying degrees of activation to specific TPA analogues indicating subtle preference for specific effectors over others depending on the TphK homolog employed in the biosensor construct.

TphK and PcaK chimeras based on the exchange of core helical domains between two active homologs were then generated and screened to assess the ability of biosensor constructs to discern mutagenic functional differences and structure activity relationships. This resulted in apparent gain of function, with TphK chimeras demonstrating uptake of pyrazine-2,5-dicarboxylic acid and naphthalene-1,6-dicarboxylic acid, whereas PcaK chimeras demonstrated increased activity to coumaric acid. These changes in activity appear to be related to the exchange of conserved residues in the core cavity interface of the protein structure, which effect the number of hydrogen bonding contacts as well as modifying the size of the core pocket, modulating effector recognition and uptake. Some of these effects were shown to be dependent upon the coordinated activity of multiple helical domains acting in tandem implicating the involvement of pairs of complementary helical pairs/bundles for correct transport function and effector recognition. This study provides a method for the usage of biosensors as screening tools for evaluating putative transporters, and library of validated TphK and PcaK homologs for biotechnologically relevant aromatic substrates.

## Supplementary Information

Below is the link to the electronic supplementary material.

Supplementary Material 1

Supplementary Material 2

## Data Availability

No datasets were generated or analysed during the current study.
