# Supplementary material for: Genetically encoded biosensor enabled mining, characterisation and engineering of aromatic acid MFS transporters
Source: J Biol Eng. 2025 Oct 31;19:94. doi: 10.1186/s13036-025-00568-y (PMC12577307; doi:10.1186/s13036-025-00568-y)
Supplement: Supplementary file 2 — Supplementary Material 2 [file 13036_2025_568_MOESM2_ESM.docx]

**Supplementary Information**

### Genetically encoded biosensor enabled mining, characterisation and engineering of aromatic acid MFS transporters

Philip Le Roy†^, Micaela Chacόn†^, Neil Dixon†*

†Manchester Institute of Biotechnology (MIB), Department of Chemistry, University of Manchester, Manchester, M17DN, UK

**Contents**

| Supplementary Table S1 – Plasmids and strains used in the study | P3 |
| --- | --- |
| Supplementary Table S2 - DNA Oligonucleotide sequences | P8 |
| Supplementary Table S3 - Sequences of expressed transporters | P9 |
| Supplementary Table S4 - TPA analogue library used in transporter screening | P41 |
| Supplementary Table S5 - PCA analogue library used in transporter screening | P43 |
| Supplementary Table S6 - PcaK Chimera raw data and processing for **Fig. 5A** | data 1 |
| Supplementary Table S7 - 2-way anova statistical analysis of data for **Fig. 4B & 5A** | data 1 |
| Supplementary Table S8 - TphK Chimera raw data and processing for **Fig. 4A & 5C** | data 1 |
| Supplementary Table S9 - 2-way anova statistical analysis of data for **Fig. 4A & 5C** | data 1 |
| Supplementary Figure 1 - Phylogenetic Tree of Rhodococcus jostii RHA1 BlastP results and example MFS hit context. | P46 |
| Supplementary Figure 2 - Comparative growth and PCA consumption of WT and ΔpcaK P. putida strains on PCA as a sole carbon source and PCA cytotoxicity. | P47 |
| Supplementary Figure 3 - Raw RFU/OD data of functional TphK and PcaK transporter screening against analogue libraries.  Supplementary Figure 4 – No transporter normalised effector screening data of TphK and PcaK biosensor constructs. | P48  P49 |
| Supplementary Figure 5 - Comparative analytics of 4,4-biphenyldicarboxylic acid consumption over 24 hours in *P. putida.* | P50 |
| Supplementary Figure 6 - Induction profile of active PcaKs against 5mM of Benzoic acid. | P51 |
| Supplementary Figure 7 - Comparison of activation of pcaV biosensor construct in Wildtype vs Δfcs P. putida.  Supplementary Figure 8 - Comparison of raw RFU vs OD600 data for PcaK-ve , PcaK_P.Put_ , TphK-ve and TphK_R.Jos_. | P52  P53 |
| Supplementary Figure 9 - Screening of TphK_R.Pyr_-PcaV and PcaK_P.Put_-TphR against structural analogues of PCA and TPA. | P54 |
| Supplementary Figure 10 - PcaK point mutant screening. | P55 |
| Supplementary Figure 11 - Multiple Sequence alignment and alphafold reference to assign helical domains for generation of chimeras. | P56 |
| Supplementary Figure 12 - Validated PcaK and TphK TM region alignment and consensus logo analysis.  Supplemental Figure 13 - MFS chimeric mutant assay normalised against the no transporter biosensor construct. | P57  P58 |
| Supplemental Figure 14 - MFS chimeric mutant assay in opposing biosensor backgrounds. | P59 |

### Table S1. Plasmids and Strains used in this study

| **Plasmid Name** | **Details** |
| --- | --- |
| pPcaK_-ve_-pcaV | PCA inducible allosteric transcription factor coupled to GFP lacking a functioning PcaK transporter |
| pTphK_-ve_-TphR | TPA inducible allosteric transcription factor coupled to GFP lacking a function TphK transporter |
| pPcaK_P.Put_-pcaV | PCA inducible allosteric transcription factor coupled to GFP with a PcaK from *Pseudomonas putida* under expression of pReg promotor and a strong G10RBS |
| pPcaK_P.Aer_-pcaV | PCA inducible allosteric transcription factor coupled to GFP with a PcaK from *Pseudomonas aeruginosa* under expression of pReg promotor and a strong G10RBS |
| pPcaK_A.Ant_-pcaV | PCA inducible allosteric transcription factor coupled to GFP with a PcaK from *Acidovorax antarcticus* under expression of pReg promotor and a strong G10RBS |
| pPcaK_A.Bay_-pcaV | PCA inducible allosteric transcription factor coupled to GFP with a PcaK from *Acinetobacter baylyi* under expression of pReg promotor and a strong G10RBS |
| pPcaK_A.Aci_-pcaV | PCA inducible allosteric transcription factor coupled to GFP with a PcaK from *Alicyclobacillus acidoterrestris* under expression of pReg promotor and a strong G10RBS |
| pPcaK_B.mas_-pcaV | PCA inducible allosteric transcription factor coupled to GFP with a PcaK from *Bacillus masiliglacei* under expression of pReg promotor and a strong G10RBS |
| pPcaK_P..Val_-pcaV | PCA inducible allosteric transcription factor coupled to GFP with a PcaK from *Paenibacillus validus* under expression of pReg promotor and a strong G10RBS |
| pPcaK_B..Sp_-pcaV | PCA inducible allosteric transcription factor coupled to GFP with a PcaK from *Bradyrhizobium* Sp. STM 3843 under expression of pReg promotor and a strong G10RBS |
| pPcaK_N.Ani_-pcaV | PCA inducible allosteric transcription factor coupled to GFP with a PcaK from *Neiserria animalis* under expression of pReg promotor and a strong G10RBS |
| pTphK_B.Pac_-TphR | TPA inducible allosteric transcription factor coupled to GFP with a TphK from *Bradyrhizobium pacchrhyzi* under expression of pReg promotor and a strong G10RBS |
| pTphK_R.Jos_-TphR | TPA inducible allosteric transcription factor coupled to GFP with a TphK from *Rhodococcus jostii* RHA1 under expression of pReg promotor and a strong G10RBS |
| pTphK_P.Um_-TphR | TPA inducible allosteric transcription factor coupled to GFP with a TphK from *Pseudomonas umsongensis* under expression of pReg promotor and a strong G10RBS |
| pTphK_R.Op_-TphR | TPA inducible allosteric transcription factor coupled to GFP with a TphK from *Rhodococcus opacus* under expression of pReg promotor and a strong G10RBS |
| pTphK_S.nap_-TphR | TPA inducible allosteric transcription factor coupled to GFP with a TphK from *Sphingobium naphthae* under expression of pReg promotor and a strong G10RBS |
| pTphK_R.Pyr_-TphR | TPA inducible allosteric transcription factor coupled to GFP with a TphK from *Rhodococcus pyridinivorans* under expression of pReg promotor and a strong G10RBS |
| pTphK_S.Hor_-TphR | TPA inducible allosteric transcription factor coupled to GFP with a TphK from *Saccharopolyspora hordei* under expression of pReg promotor and a strong G10RBS |
| pTphK_P.man_-TphR | TPA inducible allosteric transcription factor coupled to GFP with a TphK from *Pseudomonas mandelli* under expression of pReg promotor and a strong G10RBS |
| pTphK_R.Pyr_-PcaV | PCA inducible allosteric transcription factor coupled to GFP with a TphK from *Rhodococcus pyridinivorans* under expression of pReg promotor and a strong G10RBS |
| pPcaK_P.Put_-TphR | TPA inducible allosteric transcription factor coupled to GFP with a PcaK from *Pseudomonas putida* under expression of pReg promotor and a strong G10RBS |
| pPcaK_P.Put_-R124A-PcaV | Point mutant of *Pseudomonas putida* (R124A) PcaK in the PcaV biosensor background |
| pPcaK_P.Put_-R398A-PcaV | Point mutant of *Pseudomonas putida* (R289A) PcaK in the PcaV biosensor background |
| pPcaK_P.Put_-E144A-PcaV | Point mutant of *Pseudomonas putida* (R124A) PcaK in the PcaV biosensor background |
| pPcaK_TphK_TM1,4,7,10_-PcaV | PcaK from *Pseudomonas putida* containing helical domains 1, 4, 7 and 10 taken from a *Rhodococcus pyridinivorans* TphK in a PcaV biosensor background |
| pTphK_PcaK_TM1,4,7,10_-TphR | TphK from *Rhodococcus pyridinivorans* containing helical domains 1, 4, 7 and 10 taken from a *Pseudomonas putida* PcaK in a TphR biosensor background |
| pPcaK_TphK_TM1_-PcaV | PcaK from *Pseudomonas putida* containing helical domain 1 taken from a *Rhodococcus pyridinivorans* TphK in a PcaV biosensor background |
| pPcaK_TphK_TM4_-PcaV | PcaK from *Pseudomonas putida* containing helical domain 4 taken from a *Rhodococcus pyridinivorans* TphK in a PcaV biosensor background |
| pPcaK_TphK_TM7_-PcaV | PcaK from *Pseudomonas putida* containing helical domain 7 taken from a *Rhodococcus pyridinivorans* TphK in a PcaV biosensor background |
| pPcaK_TphK_TM10_-PcaV | PcaK from *Pseudomonas putida* containing helical domain 10 taken from a *Rhodococcus pyridinivorans* TphK in a PcaV biosensor background |
| pPcaK_TphK_TM4_-TphR | PcaK from *Pseudomonas putida* containing helical domain 4 taken from a *Rhodococcus pyridinivorans* TphK in a TphR biosensor background |
| pPcaK_TphK_TM7_-TphR | PcaK from *Pseudomonas putida* containing helical domain 7 taken from a *Rhodococcus pyridinivorans* TphK in a TphR biosensor background |
| pPcaK_TphK_TM10_-TphR | PcaK from *Pseudomonas putida* containing helical domain 10 taken from a *Rhodococcus pyridinivorans* TphK in a TphR biosensor background |
| pTphK_PcaK_TM1_-TphR | TphK from *Rhodococcus pyridinivorans* containing helical domain 1 taken from a *Pseudomonas putida* PcaK in a TphR biosensor background |
| pTphK_PcaK_TM4_-TphR | TphK from *Rhodococcus pyridinivorans* containing helical domain 4 taken from a *Pseudomonas putida* PcaK in a TphR biosensor background |
| pTphK_PcaK_TM7_-TphR | TphK from *Rhodococcus pyridinivorans* containing helical domain 7 taken from a *Pseudomonas putida* PcaK in a TphR biosensor background |
| pTphK_PcaK_TM10_-TphR | TphK from *Rhodococcus pyridinivorans* containing helical domain 10 taken from a *Pseudomonas putida* PcaK in a TphR biosensor background |
| pTphK_PcaK_TM1_-PcaV | TphK from *Rhodococcus pyridinivorans* containing helical domain 1 taken from a *Pseudomonas putida* PcaK in a PcaV biosensor background |
| pTphK_PcaK_TM4_-PcaV | TphK from *Rhodococcus pyridinivorans* containing helical domain 4 taken from a *Pseudomonas putida* PcaK in a PcaV biosensor background |
| pTphK_PcaK_TM7_-PcaV | TphK from *Rhodococcus pyridinivorans* containing helical domain 7 taken from a *Pseudomonas putida* PcaK in a PcaV biosensor background |
| pTphK_PcaK_TM10_-PcaV | TphK from *Rhodococcus pyridinivorans* containing helical domain 10 taken from a *Pseudomonas putida* PcaK in a PcaV biosensor background |
| **Strain Names** | **Details** |
| *P. putida:ΔpcaK* | Deletion strain of chromosomally encoded *pcaK* in *Pseudomonas putida* KT2440 for the screening of PcaK homologs |
| *P. putida:Δfcs* | Deletion strain of chromosomally encoded *fcs* in *Pseudomonas putida* KT2440 for the screening of the effect of coumaric acid on pcaV biosensor activation |

### Table S2 – DNA Oligonucleotide sequences

| **Name** | **Sequence (5’ -> 3’)** |
| --- | --- |
| XS1 | CCTGTGTGAAATTGTTatccgctt |
| XS2 | TTTTTTTCCTCCTtcaacccgg |
| PLR24 | CTCCTGTGTGAAATTGTTat |
| PLR25 | CCTCCTTATTTCAAAGTTAAAC |
| PLR44 | ccacatatccacgggctg |
| PLR50 | CCATCCAGTTCAACCAGAATCGG |
| PLR60 | taattaaagcggatAACAATTTCACACAGGCTACTTGTGGGCCAAGC |
| PLR61 | cagttgcaccgggttgaAGGAGGAAAAAAAATGTCCATTGCTCCGTC |
| PLR90 | GTTTAACTTTGAAATAAGGAGGTAATACAAATGAATCAGGCGCAAAATTCTG |
| PLR91 | attaaagcggatAACAATTTCACACAGGAGTTATGTAGCGTCCGCATGG |
| PLR119 | CAGGGCCGCGCTCATGACAGGCCCTAACGATTGCTTGCTCAGCC |
| PLR120 | GGCTTCAGTTTAGCGAGCGCATACGCGACGAACGTCACGGAATTGATGGCT |
| PLR121 | AAGGTTAAATCCACAAAACATACTCGTCACGAACGTAGCACGCAAACG |
| PLR122 | GAACTGAAATAAAGCCCCGATGAAGGCTGCGGTACCCATATCGTAGTCGA |
| PLR123 | TTGTTAGCTCTGGTCTTGATGGTGTGGTTACCTGATAGCCTCACCCTG |
| PLR124 | TTTGCGTATGCAGTTGGACAGTCCCTGGGCGCACCCCTGTGGGT |
| PLR125 | AATGGCGCCGAAGCGACCGATACCTAACATCCAACCCACACCCGTC |
| PLR102 | CAAAGCGGCGCTAAAGATAGGGCCCAAGACTGCACGATCGATTCCC |
| PLR103 | CAAGCTAAACCCGCAGTAGATGGCCAACACAAGTAAGGACTTCAGGCG |
| PLR104 | CTCGGGACCCTCGCTGCAGCCTGGTCGCAAAATGTCGATCAGCTGTTAG |
| PLR105 | TTGTTGCTCCCGGCCCTCCTCCGGTTCCTCCCGGAGAGCGCCC |
| PLR106 | GACCGTGGTCAGCGTCGTGGCCGTGACGACTTGTTCCATCGAAGCCCCG |
| PLR107 | AATCCCCCCAATCCGACCCACACCGAGAGCCCAGCTTACCCCCGTC |
| PLR108 | TTTGTGGCGCTGACGGGTTTGGCCTTGTCCAACATCACTGTTTTAGCAACTTTAGTG |
| PLR260 | AGGTGTGTACGCGCTTAACAAGGT |
| PLR261 | GAGCGCCTGAAGTCCTTACTTG |
| PLR262 | TTAGTCTTGGCTTTTCTGACTGGA |
| PLR263 | CAGCTGATCGACATTCGTCG |
| PLR264 | TTAGGTATCGGTGCCTTCGGC |
| PLR265 | CATCCAGCTTACCCCCGTC |

### Table S3 Sequences of expressed transporters

| **Name** | **Sequence (5’ -> 3’)** | | **Notes** | |  |
| --- | --- | --- | --- | --- | --- |
| PcaK_P.Put_ | ATGAATCAGGCGCAAAATTCTGTAGGTAAAAGCTTAGACGTGCAGTCATTCATCAATCAACAACCCTTGTCGCGTTACCAGTGGCGCGTCGTGTTGTTATGCTTTTTAATTGTTTTTTTGGACGGATTAGATACCGCTGCAATGGGTTTCATTGCTCCAGCGTTATCTCAAGAATGGGGAATCGATCGTGCATCGTTAGGGCCTGTCATGAGCGCGGCCCTGATCGGCATGGTTTTTGGAGCCTTGGGGTCGGGTCCGCTGGCCGACCGTTTTGGCCGTAAGGGGGTTCTGGTCGGCGCAGTACTGGTCTTCGGGGGCTTCAGTTTAGCGAGCGCATACGCGACGAATGTCGATCAGCTGTTAGTCTTGCGTTTTCTGACTGGACTGGGTCTGGGCGCCGGGATGCCGAATGCGACGACCTTGTTAAGCGAGTACACACCTGAGCGCCTGAAGTCCTTACTTGTGACGAGTATGTTTTGTGGATTTAACCTTGGCATGGCCGGCGGTGGCTTTATTTCCGCGAAGATGATTCCCGCCTACGGATGGCATAGCTTACTGGTCATTGGAGGTGTCCTGCCCTTGTTGTTAGCTCTGGTCTTGATGGTGTGGTTACCGGAGAGCGCCCGCTTTCTTGTAGTCCGTAACCGTGGGACGGATAAAATTCGTAAAACCTTGTCCCCTATTGCCCCGCAGGTTGTTGCAGAAGCAGGATCTTTTTCCGTGCCTGAACAAAAAGCTGTCGCGGCATGTAGTGTCTTCGCAGTTATCTTCAGCGGGACCTATAGGCTTGGCACAATGTTACTTTGGTTGACCTATTTTATGGGGCTTGTCATCGTATACTTACTGACTTCATGGTTGCCAACTCTGATGCGTGACTCCGGGGCTTCGATGGAACAAGCAGCCTTCATCGGGGCTTTATTTCAGTTCGGTGGGGTGTTATCCGCTGTAGGCGTCGGTTGGGCGATGGACCGCTACAATCCTCATAAGGTCATCGGAATCTTTTACTTGTTGGCAGGAGTTTTTGCGTATGCAGTTGGACAGTCCCTGGGCAACATCACTGTTTTAGCAACTTTAGTGCTGATCGCTGGGATGTGTGTTAACGGCGCCCAGTCGGCCATGCCGAGCCTTGCAGCGCGTTTTTATCCTACGCAGGGACGTGCGACGGGGGTAAGCTGGATGTTAGGTATCGGTCGCTTCGGCGCCATTTTGGGGGCTTGGTCCGGAGCCACGCTTTTAGGTCTTGGGTGGAACTTTGAACAAGTGCTTACGGCCCTGCTTGTCCCTGCAGCATTGGCCACGGTAGGCGTGATCGTTAAGGGGCTTGTTAGCCATGCGGACGCTACATAA | | | PcaK from *Pseudomonas putida,* Transmembrane domains exchanged in the creation of chimeras are highlighted accordingly:TM1, TM4, TM7, TM10 |  |
| PcaK_P.Aer_ | | ATGAACTCCCCGTCCTTGCCCGCGGTCGAACGGCTCGACGTGCAAGCATTTATTAATGCCCAGCCCCTGTCCCCCTATCAATGGCGCATCGTGTTGCTCTGCTTCTTGATTGTGTTCCTGGATGGTCTGGATACCGCCGCTATGGGTTTTATCGCACCGGCACTGACCCAGGACTGGGGGATTGATCGGGCGTCGTTGGGGCCGGTCATGTCCGCCGCACTCATCGGGATGGTGTTCGGCGCCTTGGGTTCCGGGCCATTGGCCGATCGCTACGGTCGTAAGTTGGTGTTGGTCGCGGCTGTGTTTTTGTTCGGTCTGTTTTCGTTGGCGTCCGCTTATTCGACGAACGTGGAGCAGCTCCTCGCACTGCGGTTTTTGACGGGTTTGGGGTTGGGTGCCGCAATGCCCAATGCCACCACGCTCCTGTCCGAATACACCCCAGAACGTCTGAAGAGCTTGTTGGTCACGTCGATGTTCTGCGGTTTCAACTTGGGGATGGCTTGCGGTGGTTTCGTCAGCGCGAAACTCATTCCCTTGTTTGGGTGGCATAGCTTGCTGCTGTTGGGTGGTCTCTTGCCTCTGGTGTTGGCTGTGGTCCTGCTGTTTCGCCTGCCGGAGAGCGCTCGTTACCTCGTGGTCCGGAACCGGGGTTCCGAACGTGTCCGTCAGGTGCTCGCTCCGATTGCCCCAGCTCAGGTCGCGCTGGCCCGTTCCTTCCACGTGCCCGAGCAACAAACCGTGCAGGCTCGTAATGTGTTTGCGGTGATTTTCAGCGGCACCTACAGCGCTGGGACCTTGTTGTTGTGGCTGACCTATTTTATGGGTTTGGTGATTGTGTACTTGCTCACGTCGTGGCTCCCTACCCTGATGCGCGACTCGGGGGCTTCCTTGGAGCAAGCGGCTTTTATTGGGGCGCTCTTCCAATTCGGGGGTGTGCTCAGCGCTGTGGCGGTGGGGTGGGCAATGGACCGCTTTAACCCCCATAAAGTGATCGGCCTGTTCTACTTGCTGGCCGGCGTCTTTGCTTGGTGCGTCGGGCAGTCGCTGGGCCAAGTGACCTTGTTGGCAACGTTGGTCCTGCTCGCGGGCATGTGTATCAACGGGGCACAGAGCGCCATGCCGTCGCTCGCCGCCCGCTTCTATCCTACGCAAGGGCGCGCTACGGGTGTCTCCTGGATGCTGGGCATCGGGCGGTTTGGTGCTATTCTCGGTGCCTGGATTGGCGCCACGTTGTTGGGTCTCGGCTGGAATTTTGAACAAGTCTTGACCGCTTTGGTGCTCCCGGCTGCTCTGGCGACCGCTGCCGTGCTGCTGAAGGGTCTGGTCTCGCATGCCGATGCGGGTTAG | | PcaK from *Pseudomonas aeruginosa* | |
| PcaK_A.Ant_ | | ATGTCCAGCAAGAGCCCGCGCGAATCCGGTGCTGCGGTGAACGTGCAGACCCTCCTCAATGAACATCCATTCTCCGGCTTCCAATGGCGTATTTTTGCTCTCTGCTTTTTTATCGTCCTGCTGGACGGCTTTGATACGTCGGCCATTGGGTATATTGCCCCTCACTTGATGACGGAATGGGGGGTGAGCAAGCCAGACTTGGCGCCTGTCCTGAGCGCGGCTCTCTTCGGGCTGGCGGCTGGTGCCCTGAGCGCTGGGCCACTCGCGGATAAACTGGGGCGTAAGTTGGTGTTGGTCGGGAGCGTCGCGCTCTTTGCGGCAGCCTGCCTCGCTTCCGCGTTTTCGCCAGACCTCTGGACGTTGACGGTCCTCCGGTTTATCACGGGTGTGGGCCTGGGCGCAGCAATGCCTAACGCGGTGACCCTGATGTCGGAATATTGTCCGGATGGGCGCCGCAGCACCCTGACCAATGCGATGTTTTGCGGGTTTCCGTTGGGGGCAGCATTTGGGGGGTTCTTGGCCGCGTGGATGATCCCGCATCTCGGTTGGCGGTCCGTGCTGGTGTTGGGCGGTGTGCTGCCCCTGGTCTTGGTGGTGCTGTTGTTGTGGCAGCTGCCGGAATCCGTCCGGTACATGGTGGCCAAGCAATATCCTGTGGAGCGTGTGCGCCGTGTGCTGGTGCGCATTGCAGGGGATAGCGTCCGTTCGGTGCAACGTTTTGTCATGACCGAAAGCGCTCCGGAGAGCGATGCAAAATCCGGGATTGGGGTGGTGCTCTCCGCCCCCTACCGCGTGGGCTCGGTCATGATGTGGCTGGCGTATTTTATGGGCTTGGTGATCTTCTACGCTCTGATCAACTGGATGCCGATTCTGTTTCGGGATGCAGGCCTCGATCCAAAGACCGGTGCATTGGTGGCGGCGCTGTTCCCGTTGGGGGGCGTCGGGGCTGTGTTTTTCGGCTGGCTGATGGATCGGTTTAATGCAACCCGTATTATCGCTGTGGGGTATGCATTGACCGCAGTCAGCATCTGGTGGATCGGGCAAACCGCAGGGAACCTCGGTTGGCTGGTGGTCTCCGTCTTTCTCGCTGGTACGATCATGAATACCGCTCAGTCCTCCATGCCCGCTCTGGCGGCTGCATTTTATCCAACGCGGGGTCGTGCGACGGGTGTCGCTTGGATGCTCGGGATCGGGCGTTTTGGCGGGATTGCCGGCTCCTTCTTGGTGGCGCAGTTGTCCGCCCAACAGCTGGGCTTTAGCGAAATTTTTTCGGTGGTGGCCGTGGCGGGTTTGGTGGCCACCGCGGCTCTCGTCGTGAAAGAGATCTTCTCGCCCAAGCCCCATGGTGAAGCAGTCGGCCGTACCGCAGCCCACTAG | | PcaK from *Acidovorax antarcticus* | |
| PcaK_A.Bay_ | | ATGCCCAAGGAGGCAAACATGGCCTCGCAAGACTATGCAACCCAACGGTCCTCGCTCGATGCGCAGGCACTCATCAATGATGCACCCCTGAGCCGTTACCAGTGGTTGATTGCAATTGTCTGCTTTCTGATCGTGTTCGTCGACGGGATTGATACGGCGGCAATGGGCTTTATCGCCCCAGCATTGGCACAGGACTGGGGTGTGGACCGCTCGCAGCTCGGGCCAGTCATGAGCGCGGCTCTGGGTGGGATGATCATCGGCGCATTGGTCTCCGGCCCAACCGCCGATCGGTTTGGGCGGAAGATCGTCCTCAGCATGTCCATGCTGGTGTTCGGTGGCTTCACGCTGGCTTGTGCCTACTCGACGAATTTGGACAGCCTCGTCATTTTTCGGTTCCTGACCGGGATTGGTCTGGGGGCAGCGATGCCAAATGCAACCACCTTGTTTTCCGAATACTGTCCTGCGCGCATTCGTTCGCTCCTCGTGACGTGCATGTTTTGCGGTTACAATCTCGGGATGGCAATTGGTGGCTTTATTAGCAGCTGGCTGATTCCGGCTTTTGGTTGGCATTCCCTGTTTCTGTTGGGCGGGTGGGCGCCTTTGATCCTGATGCTGCTGGTCATTTTCTTTCTGCCTGAGAGCTATCGTTTTCTGATTGTGAAAGGCAAAAATACGAAAAAGGTGCGCCAGATTTTGTCGCGGATTGCTCCCCAGAAGGTGCAAGGGGTGACCGAGTTTCATGTGCCCGAAGAGAAGGTCGAAGCCGGTACGAAGAAAGGGGTCTTTGGGATGCTCTTTAGCGCGAAATATGTGAAGGGGACGGTCCTCCTCTGGGTGACCTATTTTATGGGCCTGGTGATGATTTATCTGCTGACGTCCTGGCTGCCTACGTTGATGCGTGAAACCGGGGCATCGCTCGAACGGGCGGCCTTTCTGGGGGGGCTCTTCCAGTTTGGTGGTGTGTTGTCGGCACTCTTCATTGGCTGGGCAATGGACCGCTTTAACCCAAATCGGATTATCGCTGGGTTCTATTTGGCTGCTGGGATTTTTGCAGTGATCGTCGGCCAGTCGCTGTCCAACCCGACCTTGCTCGCCTTGTTTATCCTGTGCGCGGGTATCGCCGTGAATGGCGCTCAGTCCTCGATGCCAGTCCTGAGCGCCCGTTTTTACCCTACGCAATGCCGCGCAACCGGTGTCGCCTGGATGTCCGGCATCGGCCGGTTCGGTGCAGTCTTTGGCGCCTGGATCGGGGCAGTCCTGCTGGGTAATAATTGGTCCTTCACGATGATTCTGTCGATGCTGATTATTCCGGCGGCCGCTGCCGCCATTGCTATTTTCGTCAAATCCCTCGTCGCACATACGGATGCGACCTAG | PcaK from *Acinetobacter baylyi* | |  |
| PcaK_A.Aci_ | | ATGGCACAGGTGACGAAACTGGCAAGCTCGCGCTGGTGGCGTTTGATGCCTATCATCTTCATCACCTATTCGTTTGCGTATGTCGATCGGTCCAACTTTGGGTTTGGTGCCGCGGGTGGCATGACCGAGAGCCTCCATATTACGTCCGGCATTACCTCGTTGATCGGTGCTCTCTTCTTCCTCGGTTATTTCTTTTTTCAGATTCCTGGCGCCAACTATGCCGAAAAGAAATCCGCTAAGAAACTGCTCTTTTGGTCGATGGTGTTGTGGGGTATCTTGGCCGCCGCCACCGGTGTGGTCACCTCCGTCCCTCTGCTGTTGGTGGATCGCTTTCTGCTGGGGATCGTCGAAAGCGCTGTGTTTCCGGCCATGTTGATTCTGATTAGCAACTGGTTCGGTAAACGCGAACGTAGCCGTGCCAACACGATCTTGATTTTGGGCAATCCAGCGACGGTGTTGTGGATGAGCATTGCGAGCGGTTACTTGGTGCAATTCTTCGGGTGGCAATGGATGTTTATTATTGAGGGCTTGGTCGCAATCGTCTGGGGCGGTGTCTGGTGGTTGACGGTCAAGGACTCCCCGCAACACGCATCCTGGTTGACGGAGCAAGAGAAGCGCGACATTGCTCATGTCCTCCAATCCGAACAGACGGAACTCCAACCAGTGCCGAACTTGTGGACGGCTGCAAAATCGGGGAATGTGTTGTCGCTCATGGGTGTCTACTTTTTTTGGAGCGTGGGTATTTACGGTTTTATCATGTGGCTCCCCTCCATTCTCAAGCAGATGAGCCACGAGGGGATCGCGGCGACCGGTTGGCTGAGCGCGCTCCCATATCTCTTGGCTATTTTTGCCATGATCGGGATTTCGTACGTCAGCGACAAGACCCTGCGTCGTAAAGCGGCTGTGTGGCCCTGTCTGATGCTCGGGGGGGTCCTCCTCTACGTGTCCTATCAAATCGGGACCCAACATTTCCTGGTGAGCTATATTTTGCTGGTGATCGCGGGTGCTGCCCTGTACGCACCATATGGGCCTTTCTTCGCGGTCATCACGGAGATTTTGCCGCGTAACGTGGCAGGCGGTGCCATGGCTTTGGTGAATTCCATGGGGGCGTTGGGTTCCTTCATCGGCACGTATGTCGTGGGCCTGCTCAACGGCTCCACGGGCTCCCCCCAGGCGAGCTTTTTGCTCATGTCGATCGCTATGCTCCTGTCGGGGGGGCTCATGCTCTTCGTGCGTAAAGGGCGTCGGCGCAATGGCTCCGGGTCGGAAGATGTGGCCCTGCCTCATGTGCAGCGTTAA | PcaK from *Alicyclobacillus acidoterrestris* | |  |
| PcaK_B.Mas_ | | ATGAAAAAGATTAACGCCTTGGAGGTGGTGGCGGCGAGCAAATTTAACCGTTTTCATCTCTTCATTTTCCTGTGGTGTTTTTACGCAATTGGCTTCGACGGCTACGAGATCGTGATGTTTGGTGTCGGGTTGCCCTGGATGATGGACGAGTGGTCGCTCACCAGCATTCAAGTGGGGGCAATGGGTTCGTATAGCTTGATCGGCATGATGCTCGGTGCGCTCATCTTTGGGCCACTCGCTGATAAGCTGGGTCGGAAAAACATTCTGGCGATTTGCATGATTCTCTTCAGCCTGTTCACGCTCGGGGCCGGTTTGGCGCCGAACCCTATCTTCTTTACCGTCATGCGTTTCATCTCCGGTTTGGGTATGGGCGGCTTGATGCCCAACGTGATTTCCCTGATGACCGAATACTCCCCAAAGCGTAATCGGGCAATCATTGTCGCGACCATGTATTGTGGTTACTCGCTGGGGGGCGTGGTGGCCTCCCTGGTCGGGATGTATATCATTCCGAGCACCGATTGGCGGGTCTTGTATTGGATTGGTATCCTGCCTTTGCTCACCCTGCCTTTTTTCATGATGAAATTCCCAGAATCGCTCAGCTATTATTTGAGCCGGGGTCAGATTGACAATATTACCGACGTGTTGAAGAAAGTGGATCCGGACGGTAACTTTTCCAAAACGGATGACTACCAAATCGAAGAAATGAAAGAAAGCAGCAAGGGTTTCCCGGTGAAGAAACTGTTCACCAACAAGCGTACCGTCAGCACGTTGGCAATCTGGTTGGCGGTGTTTTCCTGCTTGCTCATGGTCTACGGTCTGAATACGTGGCTGCCCAAAATGATGCAGCAAAGCGGCTATGGGCTCACCAGCAGCCTCTCCTTCAATATGACCCTGTGCATTGGTCAGATTTTCGGTTCGGTGATTGGCGGTTACTACGCCGAGAAGATCGGTCACCGGCGCGTCTTGGTGATCTTGTTTGGGATCGGCATTTTTTGCTTCGCAGGTTTGTCCTTGACGTCGAACGTGATTCTGCTGTACCTCTTGATTCTGATCGGGGGCGCCTGCACGGTCGGCACGATGAATCTGGCAAACCCTTATATCTCGGAATATTATCCGCGTGAGGTGCGTGCAACCGGTATGGGGTGGGCGCTCGGTATTGGGCGCATCGGTGCTATCCTCGCACCAACCTTGTTTGCGATGGTCTTGGCCACCGGGATCGACTCCCAGACGGCGTTTGTCTTCTTCGCTATTCCTTCCCTGATTGCCGCTCTGGCTCTGTTGCTCATTCAAGAGAAACACGCGTCGTTCGATACCGTCGAGGACGTGAGCCTCGAGGAGGATTCGGTCCTCCACTCGATTCAGAAGGAAGTGATTAAGTAG | PcaK from *Bacillus masiliglacei* | |  |
| PcaK_P.Val_ | | ATGTCCTTCGCTGGCGTCCTCGGTGCTCAGGGTGTCCGGCTCTCGTTCGGCGCCTTCGTCGAACCTTGGCAACAGGCGTTTGGCGTGGGTCGCGATTCGATCTCCTTGGTGTCCGCGGTGTCGTTTATCGTGTATGGCGTGACGGTGCCTATTTTCGGTAAACTGACGGATCGTTTTGGCGTCACGCGTGTCTTCGCTTTCTCGGTGCTGTTCACCGGGCTGTGTACCATCGCTGTGGGGTTTACGTCCGACCTCTGGGAACTCGCCCTCGTGTATGGCGTGGCGGCTAGCATTGGCTTTGGGGGGGCATCGGGGGTGGTGGCAACGGTGGCAGTGACCCGGTGGTTTGCCGCCAAACGCGGGTTGGCATACGGTATTCTGGAAGCTGGCTTTGGTGCTGGTCAGATGATCATCGCACCCGCTTCGCTGCTGCTGATCCACTCGATCGGCTGGGAGCGCACGTCCGTGTACATGGGGCTGATGTTGATTGTGCTGTGCCCCGTGTTGCTCGCATTTTATCGTAGCAAGCCCTCGGAGAAGGGTATGTTGCCCTTGGGGGGCGTCCAAGCTGAGGAGCCCGCGCCGTCGCGGTTGGAACCTGCAAAGAAAGCGTCCACGTTGCATATTTTGAAGTTGCGCTCGTTTTGGCTCCTGTTCATTCCGTTTTTCATTTGTGGTTACACCACCACGGGGTTGATGGACACGCACCTCATCGCATACTGCCGTGGGGCCGGTTATAGCACCGCACTCACGGGTACGGCGGTCAGCGTCCTCGCTGCGTTTAACATTGTGGGTTGCCTGCTCTCGGGCAAAATTGCGGATCGCTGGAATCCGGCCATGATCCTGACGGTCCTCTACCTGATCCGTGCAGCAACGCTGCCCATTCTGTTTTTGACGTTCGATCCGGTCTGGCTGCTCGTCTTTAGCATCCTGTTCGGGCTCGTCGACTTCGCCACCGTGGCGCCAACGACGTTGCTCACGGCCGATCTGTTTCAGACCAACTCGTTGGGGTTGGTCCTGGGTCTGCTGACCTTGGGCCACCAGTTTGGCTCCGCTCTGGGTGCTTATTTTCCAGGGTGGCTGTATACGCAGACCGGCAATTATACCTTGCCTTTCTTGACCGCAGCCATCTTGTTGGTGTTTGCGTCGCTCCTGAACCTGATGTTCCCACAAGCCGTCACCAAGAAAAGCGACCTCCCCTGCTGA | PcaK from *Paenibacillus validus* | |  |
| PcaK_B.Sp_ | | ATGCCTGCCACCTCGCGTGTGGACGTCCAAACGTTCCTCGACGAACACCCTTTTAGCGGTTTCCAGTGGCTGATTTTTGGCCTGTGCTTTGTGATTGTCCTCCTCGACGGCTTTGACACCGCCGCGATTGGTTTTATCGCACCTTCGTTGCTCAAGGAATGGGGCATTGAAAAATCCGCTCTGGGCCCGGTGTTGTCCGCAGCACTGTTTGGCCTCGCCGCTGGCGCATTGTTTTCGGGCCCTCTGGCAGATCGTCTCGGCCGTAAAGTGGTGCTGGTGGCGAGCGTGCTGGTCTTCTCGTTGGCTTGTCTCGCCAGCGCCTTTGCTACCAACCTGACGGAGCTCACCGTGTTGCGTTGTGTCACCGGGATTGGCCTCGGTGCAGCTATGCCCAATGCGGTCACGCTCTTGTCCGAGTACTGCCCCCAGCGTCTGCGTGCAACGATGACCAATCTCATGTTCTGTGGTTTCCCTCTCGGCGCGGCGTTCGGCGGCTTTTTGGCAGCATGGATGATTCCGCAGTGGGGTTGGCGTTCCGTCTTGACGCTGGGGGGCTTGACCCCGCTGCTGCTCACGGTCGCCCTGGTGGTGATCTTGCCCGAATCCGTGCATTATCTGCTCAGCCGCGGCGCTTCCGTGGAGCGGGTCCGCAAGGTCATGCGCAAGATTTCGGCCGCTGCACTCCAGGTGTCGCAGTTCGTGATGTTGGAACCCGAGCACGCTGAGGTGACGCGTCGCGTGAATGCGAGCGGGTTGGGGATGGTGTTGAGCCCAGTCCTCCGTGCTGGCTCCCTCATGCTCTGGATCGCGTACTTTATGGGTCTCGTGATCTTTTATGCTTTGATTAATTGGATGCCTATCCTGTTCAAGGACGCTGGGTTGAATCCTCAAACCGCTACGTTGATTTCGGCTCTGTTTCCGCTGGGCGGGGTGGGCGCTGTCTTCTTTGGTTGGCTGATGGACCGTTTTAATGCTAACCTGATCATTGCTGCGGGGTATGCTTTGACGGCCGTCTCGATTTTTTTCATCGGCCAGGCCGCCGGGCAGCTGGGCCTGCTGATGGCTGCTGTCTTTCTGGGCGGGGTGATGATGAATACCGCCCAATCGTCGATGCCTGCCTTGGCGGCAGCGTTCTACCCTACGCAGGGGCGGGCAACCGGGGTGAGCTGGATGTTGGGTATCGGGCGTTTCGGGGGTATCGCTGGTTCGTTTCTCATCGCCGAAATGACCGCACGTCAGCTGACCTTTAGCCAAATTTTCGCGGTGATGGCTGTCCCTGGTGCTATTGCCGCAGTCGCTCTGGTCGTCAAACAGTTCGTGGGCCCGGGTCGGTCCAGCGACAAGGGGATCGGCGCAGCGGTGACGGTCCATTAA | PcaK from *Bradyrhizobium* Sp. STM 3843 | |  |
| PcaK_N.Ani_ | | ATGTCCGCAACGCGCACGATTAACGTGCAACATTTCCTCGACGAGAATAAATTCAGCCCCTACCAGTGGATGATCTTCATTATCTGTTTTCTCGTCGCTTTCTTCGACGGGATGGACACCGCGGCAATCGGTTATATTGCCCCGAGCTTGCTGGATGATTGGGGCATCGAGAAGTCCCAGCTCGCCCCAGTGCTGAGCGCAGCACTCTTCGGCTTGGCGATTGGCGCGATGACGTCGGGCCCTTTGGCGGACAAATTTGGGCGCAAACTCGTCTTGACGGCTTCGGTGCTGCTGTTCTCGGGTTTTTGCGCTGCGTCCGCATGGGCAGAAAACATCGCTCAAATGTCGGTCTTGCGTTTCATCACCGGTCTGGGTCTCGGTGCGGCGATGCCCACGGCAGTCACGTTGCTGACCGAATACTGCCCTACGCACCGTCGCGCAACCATTGTCAACACGATGTACACGGGGTTTCCGATTGGGGCCGCGGCTGGTGGCTTCTTTGCTTCCTGGTTGATCCCAGCGTATGGCTGGAAGACGGCCATCATGTGGTGCGGGCTCTTGCCCTTGGCTTTGGGGGTCCTGATGATTTTTATCATGCCGCGTTCGGTGAGCTATCTCGCCGTGACCCGCAAGCCAGCAGGTGAAATTCGGCGTATTTTGCATCGCATCAACCCGCAGGCAAACTTGGACAACGCTGAGTTCGTCATCGACGGTAAAAAAGCAGTGGCGGTGTCGTCCAACCCGATGGCGATGGTGCTGGGTAGCCACTTCCGCCTCGGCAGCACGATGCTGTGGATCTCGTACTTTATGGGCCTGATTATCTTTTATGCCATGATTAACTGGATGCCTGTGATTTTTCGTGAAGCCGGTATGCCCGCAGATTTGGGGCCAAAGATCAGCGGTCTCTTCGCGTTGGGGGGGCTGGGGGCAATCGCTAACGGTTACCTCATGGATCGCTTCAATGGGAACAAACTCATTGCACTGTTGTCCCTCCTCACCGCGGTCAGCGTGGCGTTTATTGGCTGGTCCATCGGCCAAGGGCTCGGCATGCTCGTGGCTGTCGTGTTGATTGCAGGGGTCTTCCAGAATACGGCTCAGTCGTCCTTGCCGGCACTCGCTGCGAAGTTCTACCCTGCAGAGTGTCGGACGACGGGCGTCTCCTGGATGTTGGGCATCGGTCGGTTTGGGGCTATCGCAGGGACCTTTTTGACCGGCCTGCTGTTGCAATGGAAACTCGACTTCGGGTCGATTTTTATTATCTTGGCCATTCCGTCCGTGATCATGGCAGTGTGCCTCTTGGTCAAGCAAAAGGTCTATGGCAACTCGTAA | PcaK from *Neiserria animalis* | |  |
| TphK_R.Pyr_ | | ATGTCCATTGCTCCGTCCCGCGTGACCCTGCCAGATTTCATTGACTCCCGGCCAGTGAGCCGGTATCAATATATTGTGATTGGTTTGTGCGGGCTCGTGATGTTTATTGACGGGTTCGATACGCAATCGATCTCGTATATGGCACCTCACATCGCTGAGGAATGGGGGCTGAGCAAGCAAGTCTTGGGCCCTATCTTTAGCGCCGCTTTGGCAGGCCTGATGGTCGGTTACCTGGCGCTCAGCCCCTTGAGCGAGCGGTTCGGGCACCGGCGTATGATCATCGCTAGCACCGTCCTCTTTGCACTCGGGACCCTCGCTGCAGCCTGGTCGCAAAACGTCACGGAATTGATGGCTTTGCGTTTCATCACCGGGATGGGCCTCGGGGCGGCGGCTCCTTCGGCTATCGCGTTGACCGGTGAGTACTCGCCCAAACGTTTGCGTGCTACGTTCGTGTTGGCCATCTACTGCGGGTTTAGCTTGGGCTTTGTCGCTGCGGGGTTGGTCTCGGGCTGGCTCATTCCGATTTTTGGGTGGCGTTCGGTCCTCGTCGTGGGTGCGGTCGCCCCACTCTTGTTGCTCCCGGCCCTCCTCCGGTTCCTCCCTGATAGCCTCACCCTGATGATTAACAAGGGCGCAGGTCCAGAACGTGTCCGTAGCATTCTCCGTCGCATTGATCCAGGCTTGGCTGCCGGCAGCGATTTTACGTATGAAGCCGAGACCCAGCCTGATGAGCAACGGACCGCCTTGCGGAACTTGTTCACCCGGGACCGTGTCCTGGGTACCTTGCTGCTGTGGCTCGTGTTTGTGATTAATTTGGGTGAATTTTACGCACTCCAGTCCTGGCTCCCGAGCATTATGACGAGCCTCGACTACGATATGGGTACCGTCGTCACGGCCACGACGCTGACCACGGTCGGGGGCATCGCTGCAGCTTTTGTGACGGGCCCGTGCATGGACCGTCTGGGGGCCTATTTGACGTTGGGGACGGTCTACATCGTCGGTTTTGCCTTTGTGGCGCTGACGGGTTTGGCCTTGTCCGCACCCCTGTGGGTCCTCCTGACCGCAAACTTCTTCGCCGGGGTGTGCATTTCGGGCGGTCAAAAATCGCTCATCGCTTTGTCGGCCGTGTTCTATCCAACGTCGATGCGGTCGACGGGTGTGGGTTGGGCTCTCGGTGTGGGTCGGATTGGGGGGATTGTCGGTCCTATTGTGGTCGGTGCCGCTCTGGGCATGGGCTGGTCCGCGTCCGCGGTGTTCTATGCAATGTCGATTCCTATGCTCGTGGCGGGTGTCGCGGTGCTCCTGCTCGGTCGTTGGGTGCGTAGCGACAAACACCCAGATCGTGCTGTGGCGGAATCGCAGAGCTTGGCCCACAAGTAG | TphK from *Rhodococcus pyridinivorans* Transmembrane domains exchanged in the creation of chimeras are highlighted accordingly:TM1, TM4, TM7, TM10 | |  |
| TphK_B.Pac_ | | ATGATTGCCAGCGCGCAGCCAACGAAGAACAAAAAGCTCATTGGTCCGAAACACCTGGCGTCGCGTGGCGAGGAGATGTCGTCGACGGAGGTCGCATTTGATGTCCCGACCTTTATCAATTCCCGTCGCACGGGGATTACCCAATACGTCGTGGTGGCCTTGTGTGGGTTGGTGATGTTCCTGGACGGTTTCGACACCCAAGCCATTTCGTATATGGCACCATCGATCGCGAAGGAGTTTGGTCTCACGAAACAACTGTTGGGCCCTATCTTCTCGAGCGCTTTGGCAGGCCTCATGGTCGGGTACCTGCTGGTGTCGCCATTGAGCGATCGTTTCGGGCACCGGAAGTTGGTCGTGATCTCCACGTTCGCGTTCGCGCTCCCTACGTTTGCCACGATCTTCGCTACCAATGTCGCGGAGCTCATTGTCCTGCGTTTCGTGACGGGCATCGCGCTGGGTTCCGTCATCCCGTCGGCCATTGCGCTCACGACCGAATTTTCCCCCGCACGTCTCCGTGCGACCTTCGTCCTCGCTATTTATTGTGGCTTTTCGTTGGGGTTCGTCGCAGCAGGCGCCGTCGCAGCTTGGAGCATTCCCGAATATGGGTGGCGTTCGATGCTGTGGATCGGGGCCGCCGCACCCACGGTGGTCGCCATTCTCACGCTGATGTTTTTGCCAGAATCCATGGACTTTCTGATCCGCACCAATGCCAAGCCTGATGCAGTGTGGCGGGTGGTCCGTCGTGTCGATCGCGCTCTCCCAGTCCAGGGCCCGGCCCAGTTTGTGACGGACGTGGCCGAACGTCGCTCCGCTGTCGGCTCCCTGTTTAGCAGCGGTCGTGGCGTGGGCACGCTGGTCATCTGGGCCGTCTTTATGCTGAATCTCGCAGAGTTTTATGCGCTGCAAAGCTGGCTCCCCTCCATGTTGACCAACGTGGGGCATTCCTTGAACACCGTCGCCCTCGCTACCACGATGACGACGGTGGGCGGTATTGTGGCGGCTTTCGCGATCGGTCCGGCTATGGACCGTATTGGCCCATACGGTTCCCTGGCGACCGTGTATCTCGCGGGTGTCTTGTTTGTCGTCCTCTTTGGTTACTCCATTGACAAACAATCGTGGATGATGGTGGCGGCGGCGTTCTGTGCGGGCTTTTGCATCAGCGGGGGCCAAAAATCGGTGATCGCCTTGGCGGCAATCTACTATCCGACGTCGATTCGGAGCACCGGTGTGGGGTGGGCGCTGGGGATCGGTCGCTTGGGTGGGATTGGGGGTCCACTGTTGATTGGTGCACTCTTGGCCTATCACTTGTCGGCTCAGCACATCCTCTACGTCGCCAGCGTGCCTATGCTCATTGCCGGCCTCTTGATCTTGATGCTCGGTCACTGGTGTGGGCGTGACCGGATGAAAGACCAGGGTtag | TphK from *Bradyrhizobium pacchyrhizi* | |  |
| TphK_R.Jos_ | | ATGTCGTTGGCACCCAGCCGGGTCACCCTGCCTGACTTTATTGACTCCCGGCCCGTCTCGCGGTACCAGTACATCGTGATCGCTCTGTGCGGGGTCGTGATGTTTATTGACGGCTTCGATACGCAATCCATTTCGTATATGGCGCCGCACATCGCTGAAGAATGGGGGCTGAGCAAACAGGTCCTCGGTCCCATCTTTTCCGCTGCATTGGCTGGTCTGATGGTCGGCTACTTGGCTCTGTCCCCTCTCTCCGAACGCTTCGGGCACCGCCGGATGATTCTGACGTCCACCGTGATCTTTGCCTTGGGGACGTTGGCAGCAGCATGGTCGCAAAATGTGACGGAGCTGATGGCGTTGCGCTTTATTACGGGTATGGGGCTCGGCGCGGCTGCTCCTAGCGCGATTGCGCTGACCGGTGAGTTTTCGCCGAAACGTCTGCGGGCCACGTTTGTCCTCGTCATCTATTGCGGGTTCTCCTTGGGGTTCGTGGCAGCGGGTTTGGTCTCCGGCTGGCTGATTCCGATTCTGGGGTGGCGGTCCGTCTTGGTCGTCGGCGCCGTCGCCCCCTTGTTGCTCCTGCCTGCGCTGTTGCGTTACCTCCCAGATTCGTTGACGTCCATGATTAACCGGGGTGCAGAGCCCAATCGCATTCAGGCGATCTTTCGCAAAATGGACCCGGCCCTCGCAGTGGGTCCCGACATCACCTACGAGGCCGAGAAACGCACCGATGGTCAGCGCACCGCTCTGCGCTCGCTGTTTACGCGCGACCGCGTGCTGGGTACGCTCCTGCTCTGGCTGGTGTTCGTGATTAACTTGGGCGAATTCTATGCACTGCAGTCGTGGCTGCCATCGATTATGACCAGCCTCGATTATGATATGGGGACGGTGGTCACGGCCACGACGCTGACGACGGTCGGGGGGATTGCTGCAGCTTTCGTCACGGGGCCCTGCATGGATCGGTTGGGCGCGTATGTCACCTTGGGTACCGTCTATGTCGTCGGCTTTGCATTTGTGGCACTGACGGGTGTGGCGTTTACGGCTCCCCTCTGGGTGCTGCTCACCGCGAACTTTTTCGCTGGTGTGTGTATTAGCGGGGGGCAGAAATCCCTGATTGCCCTCTCCGCCGTGTTTTACCCTACCCCCATGCGTTCCACGGGTGTGGGTTGGGCACTCGGTGTGGGCCGTCTGGGGGGCATCGTCGGTCCGATTGCAGTCGGGGCGGCTTTGGGCATGGGTTGGTCCGCCTCGGCCGTGTTTTATGCTATGTCCGTGCCCATGCTCGTCGCGGGGGCGGCGGTCTTTTTGTTGGGTCGGTGGGTCCGGAGCGACAATCACCCCGACCGTAAGAGCGCCGAATCCCATTCCCTGGCGCGTAAGTAA | TphK from  *Rhodococcus Jostii* | |  |
| TphK_P.Um_ | | ATGCTCCCCGAATTGAAACCCTTGCAATTGCCGCAGTTTGTGGATGAACGTCCTGTCTCCCCGTTTCAATACGTGGTCATTATTCTGTGTGGCCTGGTGATGTTCCTGGACGGGTTTGACACCCAAGCGATTTCGTATATGGCACCTTATATCGCATCGGAATGGAACCTGCCTCGGCAAATGCTCGGCCCTATCTTTTCCTCGTCGCTCGCGGGGCTCATGATTGGCTATCTCGTGTTGACCCCGCTCTCGGAGCGTTTTGGGCATAAGAAAGCCATTATCGTGTCGACGATCGTCTTTTCCCTGTGCACCTTGGCGTCCGTGTGGGCAGACAATGTCACCGAGCTGATGATTTTGCGGTTCATTACGGGTGTCGGCCTCGGGGCCGCTGCACCGAGCGCGATTGCGATGACGGGGGAGTATTCGCCTAAGCGTTTCCGCGCAACCTTTGTCTTGGCCATTTACTGTGGCTTTAGCTTGGGTTTCATCGCCGCTGGTTTGGCGGCTAATTGGTTCATCCCCCACTATGGCTGGCGGTCGATGTTCTGGATCGGTGCTCTCGCGCCGCTCATGCTCGTCCCCGTGCTCATCCGGTTCCTGCCTGAATCCATGGTCTTCATGATTCATCGTAAAGTGGCCGCTGAGCGCATTCTCAAAATCTTCCGGAAGATCGACCGTGCAGTCGCTCGCCAGGAGAAACCAACGTTCATTGTCGATGCCTTGGAGCAAGGTCAGGGGCAACGGGGGGCCCTGGCAAGCCTGTTTACCCCAGATCGCGTGCTCGGGACGGTGCTGTTGTGGTTGGTGTTCGCTATTAATTTGGGTGAGTTCTATGCCCTCCAATCCTGGCTCCCGTCCATCATGACCGGTTTGAATTATCCAATGTCGACGGTCGTGATGGCTACCACCTTGACCACGGTCGGTGGTATCGCCGCTGCATTCATTACGGGCCCTAGCATGGACCGCTTGGGGGCTTATAAAACCCTGGGCATTTTGTACTTGGTCGGCTTTGTGTGCGTGGCTTTGACCGGTTTGGCATTCAAGAGCCCATTGTGGATGCTCTTGACGGCCAATTTTTTGGCTGGTTGCTGCATTTCGGGTGGCCAAAAGAGCCTCATCGCTTTGGCAGCTGTCTTCTACCCAGCCCCGATTCGTAGCACGGGTGTGGGCTGGGCTTTGGGCATCGGTCGCATTGGGGGGATCACCGGGCCAATCGTCGTGGGTGCGGCACTCGGTATGGGCTGGTCCCCGTCCTCCGTCTTTTATGCCATGGCGGTGCCGATGTTGATTGCAGGGCTCCTCGTGCTGTTCTTGGGCCGTCGGTACGGCAGCAGCACGCTCTAA | TphK from *Pseudomonas umsongensis* | |  |
| TphK_R.Op_ | | ATGTCCCTGGCGCCTAGCCGTGTCAAGTTGCCAGACTTTATCGACTCGCGGCCGGTCTCGCGTTATCAGTATATCGTGATCGCTCTCTGTGGTGTCGTGATGTTTATCGATGGTTTTGACACGCAATCGATCTCGTATATGGCCCCCCACATTGCCGAAGAGTGGGGTCTCTCGAAGCAAGTCCTCGGGCCCATCTTTTCCGCCGCACTGGCTGGCCTGATGGTCGGTTACCTCGCCCTGAGCCCCTTGTCGGAACGCTTCGGCCATCGGCGCATGATCCTGACGAGCACGGTGATTTTTGCTCTGGGTACCTTGGCAGCTGCCTGGTCCCAGAATGTGACCGAATTGATGGCGTTGCGCTTTATTACGGGGATGGGTCTGGGCGCTGCGGCTCCGAGCGCAATCGCTCTCACCGGCGAGTTCTCCCCAAAACGTTTGCGCGCTACCTTCGTCTTGGTCATTTATTGCGGTTTTAGCCTGGGTTTTGTGGCCGCTGGCCTCGTGTCCGGGTGGCTGATCCCGATTCTCGGGTGGCGCTCGGTGCTGGTCGTCGGTGCGGTGGCCCCGCTGTTGCTGCTCCCGGCTCTCCTCCGTTATTTGCCAGATAGCCTCACGTCCATGATTAACCGTGGTGCAGAACCTAATCGCATTCAGGCAATTTTTCGTAAGATGGACCCCGCCTTGGCCGTCGGCCCCGATATTACGTATGAGGCCGAAAAACGTACGGATGGTCAACGGACGGCCTTGCGGAGCCTGTTCACCCGTGACCGCGTCTTGGGTACGTTGTTGTTGTGGCTGGTCTTTGTCATTAACTTGGGTGAATTCTACGCTCTCCAATCGTGGCTGCCTTCGATCATGACCTCGCTGGATTATGACATGGGTACCGTCGTGACCGCCACGACGCTCACCACGGTCGGGGGGATCGCCGCGGCGTTCGTCACCGGGCCATGTATGGACCGTCTCGGTGCTTACGTGACGCTCGGCACGGTGTACGTCGTCGGTTTCGCTTTTGTGGCATTGACCGGCGTGGCATTTACCGCACCCTTGTGGGTGCTGCTCACGGCTAACTTTTTCGCGGGCGTGTGTATCTCCGGTGGGCAGAAGTCCCTGATTGCGTTGTCCGCCGTCTTCTACCCCACGAGCATGCGTAGCACCGGGGTCGGCTGGGCCCTCGGGGTCGGTCGTCTCGGGGGTATCGTCGGCCCCATTGCGGTGGGGGCAGCTTTGGGCATGGGTTGGTCCGCTTCCGCTGTGTTCTACGCTATGTCCGTCCCGATGTTGGTCGCAGGTGCTGCTGTGTTTCTCCTCGGTCGCTGGGTCCGCTCCGATAATCACCCCGACCGCAAATCGGCAGAGAGCCATTCCCTCGCACGCAAGTAA | TphK from *Rhodococcus jostii* | |  |
| TphK_S.Nap_ | | ATGACGGCGCAGGCCTCGCCATCCATCGCGACCTTGATCGATGATCGGCCGATGCGGCCCTCGCAGTGGATGACCGTGTTGATGTGTGGGTTGGTCATGTTTTTGGACGGTTTCGATACCCAGGCCATTTCCTATATGGCTCCGCACATCGCAGAAGAGTGGGACCTGCCCCGGGCTATGCTCGGGCCCATCTTTTCGGCGGCTCTGGTCGGTCTCATGGTGGGCTATTTGTTTTTTTCCCCATTGTCCGATCGTTTTGGTCATAAGCGCATGGTCGTGTGGTCGACCGCTGCGTTCGCGGCCAGCACGTTGTTGTGTTTGATTGCCGGTAATGTCACGACGCTGGTGCTGCTCCGGTTTCTGACCGGTGTGGGGTTGGGGGCGGCTACGCCTAGCGCGGTCGCATTGACGTCGGAATATAGCCCCAAACGGTGGCGCGCTAGCTTTGTGCTGGCTATTTACTGTGGGTTTTCCTTGGGCTTTGTGGCTGCGGGGTTGGCCGCGGGCTGGCTCATTCCTGCCCATGGGTGGCGTTCGCTCTTTTGGATTGGGGCTATTTTGCCGTTGGCTCTCGTCCCTATTCTCTTCTTGTTCCTCCCTGAGGCAATTACGACCTTGACGCGTCCTGGCGCCGACCGCGATGTCGCCTTGGCCCAGTTTCGCCGCTTGGACCCGGCCTTTCGCCCGGAGGACCTGCCACATGCTCCTGTCGGGGAGACCGGGGCCCGCCCCCGGGCACCCCTGCGCATTATTCTGGCTTCCAATCTCCGCTTTGGTACCCTCCTCCTGTGGCTCGTCTTTGTCATTAATTTGGGTACGTTCTACGCTATGCAGTCGTGGATGCCCACGATCTTGACGGACTTGGGCTATCCTACCGGCACCGTCGTGGCTACGACCACGATCTTTACCGTCGGTGGTATTGTCGCTGCTCTGATTGTCGGCCCGTTGATGGATCGGGTGTCGCCTTACCTGGCTCTGGCCGGTTTGTACGCCGCTGGGTTTGTGTGCGTCGGCGCTATCGGTTTGCTCCTGGGGAGCGGCGCCGCCGCCCTGCTCGCCACCTCGTTCTTGGCGGGGATCTGCGTCAGCGGGGGGCAGAAAAGCGTCATCGCGTTGGCCGCAGTCTTCTACGACGCCCAAGTCCGGTCGACGGGCGTGGGGTGGGCGCTGGGGATTGGTCGTGTCGGTGGCATCGGTGGTCCGCTGATCGTCGGGACCGCATTGGGTGCGGGCCTGAGCCCGCAAGGCGTCTTTTATATGTTGGCGGTGCCAATGCTCGTGATGGTCGCGGCTATGCTCATGTTGTCCGCAAACGCTCGTCGTGTGCGCGCTGATGCGTCGCGTGCTGCACAAGAGGCGCCACACGGGtag | TphK from *Sphingobium naphthae* | |  |
| TphK_S.Hor_ | | ATGCCAGGGTTTGTGGATGATCGTCGTCCCAACCGTTTTCAGCTGGACACCGTGTTGCTCTGCGGCCTCGTGATGTTCTTGGATGGTTTCGATACGCAGGCGATTAACTATATGGCACCCGCCATTACCGAGGACTGGGGTTTGAGCAAAGCGGACCTGGGCCCCATCTTCTCGGCGGCCCTGGCCGGCTTGATGATCGGCTACTTGGTCTTGTCGCCACTGGCTGATCGCTACGGCCATAAACGCTTGGTGGTCGGTGGTGCGACCTTGTTCGCGCTGACCACCTTTGCATCGGCTTGGGCACGCGATCCCGGTCAGTTGCTCCTGTTGCGCTTTATCACGGGTATGGGTTTGGGGGCTGCAACGCCGAGCACGATTGCCCTGACGGCCGAGTATTCGCCAAAGCGTGTGCGCGCTTCGTTTGTCCTGGCAATCTATTGCGGTTTTAGCTTGGGGTTTGTCGTCGCAAATTATGTCGCAGATGCACTGATCCCCGTCCACGGTTGGCGCTCGGTGTTTTTGGTGGGCGGGGTCGCCCCTTTGTTGCTGGTCGTCGTGCTCTTGCGGGCATTGCCTGAAAGCATGTCGTATCTCCTGCGTCGTGGGGACACGGGTCGCGCCTACGCTCTCTGTCGCAAAATTGACCCAACCTTGCCAACCGGGTCGGCTCCTGAGATTGAAACGGAAGATGCGGCCCAAGGTAAACGTGTGACGCTGCGTGAGCTCGTCGTCGGGGGCCGCCTCGGCAGCACCGTGCTGCTGTGGACGGTGTTCGTGATTAATTTGGGCATGTTTTATGGGCTCCAGTCGTGGATGCCCACGATTCTCTCCGAGCGGGGCCATTCCGCAGCTACCCTGGCTACCGCTTCCTCGATGACCACCATCGGGGGTATTGCCATCGCGTTCGTCATCGGGCCCGCCATGGACCGGCTGGGCACGTTCCGCTCCCTCGGTACGTTGTATCTGGTGGGTTGCGCATTTCTCGTCCTGATGAGCGTCGCACTCGGCGCCCCGGTGTGGCTCTTGATGACGGCAACCTTCCTCATCGGCTGTGTGGTGTCGGGTGGCCAGAAGAGCGTCATTGCCCTGGCTGCAGTCTTCTATCCCGCTGAGGTCCGTTCGACGGGCGTCGGCTGGGCCTTGGGTATTGGTCGTGCGGGCGGCATCGCAGGCCCGCTCATCGTGGGGGGCGTCCTGTCCGCTGGCTGGTCGGCTGAGACGTTGTTCCTGGCGCTGGTGATCCCCTCGGCGATTTGCGGCCTCATCGTCCTCCATCTGGGGCGCCGTGCAGCTAGCtag | TphK from *Saccharopolyspora hordei* | |  |
| TphK_P.Man_ | | ATGCTCCCGGAGTCCAAGCCGCTGCAGCTCCCACAGTTTGTCGATGAGCGTCGCGTGTCCCCTTTTCAATATGGGGTCATCATCCTGTGTGGCTTGGTCATGTTCCTGGATGGCTTCGATACCCAAGCAATTAGCTATATGGCGCCTTATATCGCGCAGGAATGGTCCCTCTCGAAGCAGATGTTGGGCCCAATTTTCTCCAGCTCCTTGGCTGGTCTGATGGTCGGGTACCTCGTCCTGACGCCTTTGTCGGAGCGTTTTGGGCATAAGAAGGCCATTATTGTGTCGACGATCGTGTTTTCCCTCTGCACCCTCGCTTCGGTGTGGGCAACGAACGTCACGGAATTGATGGTCTTGCGGTTCATTACGGGTATCGGGTTGGGCACGGCCGCCCCTTCGGCAATCGCAATGACCGGTGAGTACTCCCCGAAGCGGCTGCGCGCTACCTTCGTCCTGGCAATCTATTGCGGTTTCTCCCTGGGCTTCATCGCAGCAGGGTTGGCCGCAGGCTGGTTTATCCCGCATTATGGTTGGCGGTCGATGTTTTGGGTCGGTGCTCTGGCCCCGCTGATCTTGGTGCCCGTCCTGTTTCGTTTTCTCCCAGAGTCGATGGTGTTTATGATCAAAAATAAGGTCCCCCCACAACAAATCTTGAACGTCTTTCGTAAGATCGATGCAAGCGTCTCGCAGCAGAGCAAACCTGTCTTCGTGGTCGAAGCACTCGAACAGGGCCAACACGGCGCTCTCGCGAGCCTGTTCACCCGTGACCGCATCATGGGTACGGTGCTGTTGTGGTTGGTGTTCGCAATCAACCTGGGTGAATTTTATGCCCTCCAGTCGTGGCTGCCGAGCATCATGACGGGGTTGAATTACCCGATGAGCACGGTCGTCATGGCGACCACCTTGACCACGGTCGGGGGTATTGCGGCAGCTTTCATTACGGGGCCAAGCATGGACCGCTTGGGTGCCTATAAAACCGTGGGTATTCTCTACTTGGTCGGCTTCGTCTTTGTGGCCCTGACCGGCATGGCATTTAACTCGCCGTTGTGGGTCTTGTTGAGCGCTAATTTTCTGGCTGGGTGCTGCATTTCGGGTGGTCAGAAAAGCCTCATTGCACTCGCTGCAGTGTTCTACCCGGCGCCAATGCGGTCCACCGGTGTCGGTTGGGCTCTGGGTATTGGCCGGATCGGCGGGATCGCGGGGCCTATTGTCGTGGGCGCAGCTTTGGGTATTGGCTGGTCGCCGAGCGCGGTGTTTTACGGGATGGCAATCCCAATGCTGATTGCCGGTTTGATCGTGCTCTTTCTCGGTCGCCGCTATGGGAACTCCGATAAGGTGTAA | TphK from *Pseudomonas mandelli* | |  |
| PcaK_TphK_TM1,4,7,10_ | | ATGTCCATTGCTCCGTCCCGCGTGACCCTGCCAGATTTCATTGACTCCCGGCCAGTGAGCCGGTATCAATATATTGTGATTGGTTTGTGCGGGCTCGTGATGTTTATTGACGGGTTCGATACGCAATCGATCTCGTATATGGCACCTCACATCGCTGAGGAATGGGGGCTGAGCAAGCAATCGTTAGGGCCTGTCATGAGCGCGGCCCTGATCGGCATGGTTTTTGGAGCCTTGGGGTCGGGTCCGCTGGCCGACCGTTTTGGCCGTAAGGGGGTTCTGGTCGGCGCAGTACTGGTCTTCGGGGGCTTCAGTTTAGCGAGCGCATACGCGACGAACGTCACGGAATTGATGGCTTTGCGTTTCATCACCGGGATGGGCCTCGGGGCGGCGGCTCCTTCGGCTATCGCGTTGACCGGTGAGTACTCGCCCAAACGTTTGCGTGCTACGTTCGTGACGAGTATGTTTTGTGGATTTAACCTTGGCATGGCCGGCGGTGGCTTTATTTCCGCGAAGATGATTCCCGCCTACGGATGGCATAGCTTACTGGTCATTGGAGGTGTCCTGCCCTTGTTGTTAGCTCTGGTCTTGATGGTGTGGTTACCTGATAGCCTCACCCTGATGATTAACAAGGGCGCAGGTCCAGAACGTGTCCGTAGCATTCTCCGTCGCATTGATCCAGGCTTGGCTGCCGGCAGCGATTTTACGTATGAAGCCGAGACCCAGCCTGATGAGCAACGGACCGCCTTGCGGAACTTGTTCACCCGGGACCGTGTCCTGGGTACCTTGCTGCTGTGGCTCGTGTTTGTGATTAATTTGGGTGAATTTTACGCACTCCAGTCCTGGCTCCCGAGCATTATGACGAGCCTCGACTACGATATGGGTACCGCAGCCTTCATCGGGGCTTTATTTCAGTTCGGTGGGGTGTTATCCGCTGTAGGCGTCGGTTGGGCGATGGACCGCTACAATCCTCATAAGGTCATCGGAATCTTTTACTTGTTGGCAGGAGTTTTTGCGTATGCAGTTGGACAGTCCCTGGGCGCACCCCTGTGGGTCCTCCTGACCGCAAACTTCTTCGCCGGGGTGTGCATTTCGGGCGGTCAAAAATCGCTCATCGCTTTGTCGGCCGTGTTCTATCCAACGTCGATGCGGTCGACGGGTGTGGGTTGGATGTTAGGTATCGGTCGCTTCGGCGCCATTTTGGGGGCTTGGTCCGGAGCCACGCTTTTAGGTCTTGGGTGGAACTTTGAACAAGTGCTTACGGCCCTGCTTGTCCCTGCAGCATTGGCCACGGTAGGCGTGATCGTTAAGGGGCTTGTTAGCCATGCGGACGCTACATAA | PcaK from *Pseudomonas putida* containing helical domains from a *Rhodococcus pyridinivorans* TphK indicated accordingly: TM1, TM4, TM7, TM10 | |  |
| TphK_PcaK_TM1,4,7,10_ | | ATGAATCAGGCGCAAAATTCTGTAGGTAAAAGCTTAGACGTGCAGTCATTCATCAATCAACAACCCTTGTCGCGTTACCAGTGGCGCGTCGTGTTGTTATGCTTTTTAATTGTTTTTTTGGACGGATTAGATACCGCTGCAATGGGTTTCATTGCTCCAGCGTTATCTCAAGAATGGGGAATCGATCGTGCAGTCTTGGGCCCTATCTTTAGCGCCGCTTTGGCAGGCCTGATGGTCGGTTACCTGGCGCTCAGCCCCTTGAGCGAGCGGTTCGGGCACCGGCGTATGATCATCGCTAGCACCGTCCTCTTTGCACTCGGGACCCTCGCTGCAGCCTGGTCGCAAAATGTCGATCAGCTGTTAGTCTTGCGTTTTCTGACTGGACTGGGTCTGGGCGCCGGGATGCCGAATGCGACGACCTTGTTAAGCGAGTACACACCTGAGCGCCTGAAGTCCTTACTTGTGTTGGCCATCTACTGCGGGTTTAGCTTGGGCTTTGTCGCTGCGGGGTTGGTCTCGGGCTGGCTCATTCCGATTTTTGGGTGGCGTTCGGTCCTCGTCGTGGGTGCGGTCGCCCCACTCTTGTTGCTCCCGGCCCTCCTCCGGTTCCTCCCGGAGAGCGCCCGCTTTCTTGTAGTCCGTAACCGTGGGACGGATAAAATTCGTAAAACCTTGTCCCCTATTGCCCCGCAGGTTGTTGCAGAAGCAGGATCTTTTTCCGTGCCTGAACAAAAAGCTGTCGCGGCATGTAGTGTCTTCGCAGTTATCTTCAGCGGGACCTATAGGCTTGGCACAATGTTACTTTGGTTGACCTATTTTATGGGGCTTGTCATCGTATACTTACTGACTTCATGGTTGCCAACTCTGATGCGTGACTCCGGGGCTTCGATGGAACAAGTCGTCACGGCCACGACGCTGACCACGGTCGGGGGCATCGCTGCAGCTTTTGTGACGGGCCCGTGCATGGACCGTCTGGGGGCCTATTTGACGTTGGGGACGGTCTACATCGTCGGTTTTGCCTTTGTGGCGCTGACGGGTTTGGCCTTGTCCAACATCACTGTTTTAGCAACTTTAGTGCTGATCGCTGGGATGTGTGTTAACGGCGCCCAGTCGGCCATGCCGAGCCTTGCAGCGCGTTTTTATCCTACGCAGGGACGTGCGACGGGGGTAAGCTGGGCTCTCGGTGTGGGTCGGATTGGGGGGATTGTCGGTCCTATTGTGGTCGGTGCCGCTCTGGGCATGGGCTGGTCCGCGTCCGCGGTGTTCTATGCAATGTCGATTCCTATGCTCGTGGCGGGTGTCGCGGTGCTCCTGCTCGGTCGTTGGGTGCGTAGCGACAAACACCCAGATCGTGCTGTGGCGGAATCGCAGAGCTTGGCCCACAAGTAG | TphK from *Rhodococcus pyridinivorans* containing helical domains from a *Pseudomonas putida* PcaK indicated accordingly: TM1, TM4, TM7, TM10 | |  |
| PcaK_TphK_TM1_ | | ATGTCCATTGCTCCGTCCCGCGTGACCCTGCCAGATTTCATTGACTCCCGGCCAGTGAGCCGGTATCAATATATTGTGATTGGTTTGTGCGGGCTCGTGATGTTTATTGACGGGTTCGATACGCAATCGATCTCGTATATGGCACCTCACATCGCTGAGGAATGGGGGCTGAGCAAGCAATCGTTAGGGCCTGTCATGAGCGCGGCCCTGATCGGCATGGTTTTTGGAGCCTTGGGGTCGGGTCCGCTGGCCGACCGTTTTGGCCGTAAGGGGGTTCTGGTCGGCGCAGTACTGGTCTTCGGGGGCTTCAGTTTAGCGAGCGCATACGCGACGAATGTCGATCAGCTGTTAGTCTTGCGTTTTCTGACTGGACTGGGTCTGGGCGCCGGGATGCCGAATGCGACGACCTTGTTAAGCGAGTACACACCTGAGCGCCTGAAGTCCTTACTTGTGACGAGTATGTTTTGTGGATTTAACCTTGGCATGGCCGGCGGTGGCTTTATTTCCGCGAAGATGATTCCCGCCTACGGATGGCATAGCTTACTGGTCATTGGAGGTGTCCTGCCCTTGTTGTTAGCTCTGGTCTTGATGGTGTGGTTACCGGAGAGCGCCCGCTTTCTTGTAGTCCGTAACCGTGGGACGGATAAAATTCGTAAAACCTTGTCCCCTATTGCCCCGCAGGTTGTTGCAGAAGCAGGATCTTTTTCCGTGCCTGAACAAAAAGCTGTCGCGGCATGTAGTGTCTTCGCAGTTATCTTCAGCGGGACCTATAGGCTTGGCACAATGTTACTTTGGTTGACCTATTTTATGGGGCTTGTCATCGTATACTTACTGACTTCATGGTTGCCAACTCTGATGCGTGACTCCGGGGCTTCGATGGAACAAGCAGCCTTCATCGGGGCTTTATTTCAGTTCGGTGGGGTGTTATCCGCTGTAGGCGTCGGTTGGGCGATGGACCGCTACAATCCTCATAAGGTCATCGGAATCTTTTACTTGTTGGCAGGAGTTTTTGCGTATGCAGTTGGACAGTCCCTGGGCAACATCACTGTTTTAGCAACTTTAGTGCTGATCGCTGGGATGTGTGTTAACGGCGCCCAGTCGGCCATGCCGAGCCTTGCAGCGCGTTTTTATCCTACGCAGGGACGTGCGACGGGGGTAAGCTGGATGTTAGGTATCGGTCGCTTCGGCGCCATTTTGGGGGCTTGGTCCGGAGCCACGCTTTTAGGTCTTGGGTGGAACTTTGAACAAGTGCTTACGGCCCTGCTTGTCCCTGCAGCATTGGCCACGGTAGGCGTGATCGTTAAGGGGCTTGTTAGCCATGCGGACGCTACATAA | PcaK from *Pseudomonas putida* containing a helical domain from a *Rhodococcus pyridinivorans* TphK indicated accordingly: TM1 | |  |
| PcaK_TphK_TM4_ | | ATGAATCAGGCGCAAAATTCTGTAGGTAAAAGCTTAGACGTGCAGTCATTCATCAATCAACAACCCTTGTCGCGTTACCAGTGGCGCGTCGTGTTGTTATGCTTTTTAATTGTTTTTTTGGACGGATTAGATACCGCTGCAATGGGTTTCATTGCTCCAGCGTTATCTCAAGAATGGGGAATCGATCGTGCATCGTTAGGGCCTGTCATGAGCGCGGCCCTGATCGGCATGGTTTTTGGAGCCTTGGGGTCGGGTCCGCTGGCCGACCGTTTTGGCCGTAAGGGGGTTCTGGTCGGCGCAGTACTGGTCTTCGGGGGCTTCAGTTTAGCGAGCGCATACGCGACGAACGTCACGGAATTGATGGCTTTGCGTTTCATCACCGGGATGGGCCTCGGGGCGGCGGCTCCTTCGGCTATCGCGTTGACCGGTGAGTACTCGCCCAAACGTTTGCGTGCTACGTTCGTGACGAGTATGTTTTGTGGATTTAACCTTGGCATGGCCGGCGGTGGCTTTATTTCCGCGAAGATGATTCCCGCCTACGGATGGCATAGCTTACTGGTCATTGGAGGTGTCCTGCCCTTGTTGTTAGCTCTGGTCTTGATGGTGTGGTTACCTGATAGCCTCACCCTGATGATTAACAAGGGCGCAGGTCCAGAACGTGTCCGTAGCATTCTCCGTCGCATTGATCCAGGCTTGGCTGCCGGCAGCGATTTTACGTATGAAGCCGAGACCCAGCCTGATGAGCAACGGACCGCCTTGCGGAACTTGTTCACCCGGGACCGTGTCCTGGGTACCTTGCTGCTGTGGCTCGTGTTTGTGATTAATTTGGGTGAATTTTACGCACTCCAGTCCTGGCTCCCGAGCATTATGACGAGCCTCGACTACGATATGGGTACCGCAGCCTTCATCGGGGCTTTATTTCAGTTCGGTGGGGTGTTATCCGCTGTAGGCGTCGGTTGGGCGATGGACCGCTACAATCCTCATAAGGTCATCGGAATCTTTTACTTGTTGGCAGGAGTTTTTGCGTATGCAGTTGGACAGTCCCTGGGCGCACCCCTGTGGGTCCTCCTGACCGCAAACTTCTTCGCCGGGGTGTGCATTTCGGGCGGTCAAAAATCGCTCATCGCTTTGTCGGCCGTGTTCTATCCAACGTCGATGCGGTCGACGGGTGTGGGTTGGATGTTAGGTATCGGTCGCTTCGGCGCCATTTTGGGGGCTTGGTCCGGAGCCACGCTTTTAGGTCTTGGGTGGAACTTTGAACAAGTGCTTACGGCCCTGCTTGTCCCTGCAGCATTGGCCACGGTAGGCGTGATCGTTAAGGGGCTTGTTAGCCATGCGGACGCTACATAA | PcaK from *Pseudomonas putida* containing a helical domain from a *Rhodococcus pyridinivorans* TphK indicated accordingly: TM4 | |  |
| PcaK_TphK_TM7_ | | ATGAATCAGGCGCAAAATTCTGTAGGTAAAAGCTTAGACGTGCAGTCATTCATCAATCAACAACCCTTGTCGCGTTACCAGTGGCGCGTCGTGTTGTTATGCTTTTTAATTGTTTTTTTGGACGGATTAGATACCGCTGCAATGGGTTTCATTGCTCCAGCGTTATCTCAAGAATGGGGAATCGATCGTGCATCGTTAGGGCCTGTCATGAGCGCGGCCCTGATCGGCATGGTTTTTGGAGCCTTGGGGTCGGGTCCGCTGGCCGACCGTTTTGGCCGTAAGGGGGTTCTGGTCGGCGCAGTACTGGTCTTCGGGGGCTTCAGTTTAGCGAGCGCATACGCGACGAATGTCGATCAGCTGTTAGTCTTGCGTTTTCTGACTGGACTGGGTCTGGGCGCCGGGATGCCGAATGCGACGACCTTGTTAAGCGAGTACACACCTGAGCGCCTGAAGTCCTTACTTGTGACGAGTATGTTTTGTGGATTTAACCTTGGCATGGCCGGCGGTGGCTTTATTTCCGCGAAGATGATTCCCGCCTACGGATGGCATAGCTTACTGGTCATTGGAGGTGTCCTGCCCTTGTTGTTAGCTCTGGTCTTGATGGTGTGGTTACCTGATAGCCTCACCCTGATGATTAACAAGGGCGCAGGTCCAGAACGTGTCCGTAGCATTCTCCGTCGCATTGATCCAGGCTTGGCTGCCGGCAGCGATTTTACGTATGAAGCCGAGACCCAGCCTGATGAGCAACGGACCGCCTTGCGGAACTTGTTCACCCGGGACCGTGTCCTGGGTACCTTGCTGCTGTGGCTCGTGTTTGTGATTAATTTGGGTGAATTTTACGCACTCCAGTCCTGGCTCCCGAGCATTATGACGAGCCTCGACTACGATATGGGTACCGCAGCCTTCATCGGGGCTTTATTTCAGTTCGGTGGGGTGTTATCCGCTGTAGGCGTCGGTTGGGCGATGGACCGCTACAATCCTCATAAGGTCATCGGAATCTTTTACTTGTTGGCAGGAGTTTTTGCGTATGCAGTTGGACAGTCCCTGGGCAACATCACTGTTTTAGCAACTTTAGTGCTGATCGCTGGGATGTGTGTTAACGGCGCCCAGTCGGCCATGCCGAGCCTTGCAGCGCGTTTTTATCCTACGCAGGGACGTGCGACGGGGGTAAGCTGGATGTTAGGTATCGGTCGCTTCGGCGCCATTTTGGGGGCTTGGTCCGGAGCCACGCTTTTAGGTCTTGGGTGGAACTTTGAACAAGTGCTTACGGCCCTGCTTGTCCCTGCAGCATTGGCCACGGTAGGCGTGATCGTTAAGGGGCTTGTTAGCCATGCGGACGCTACATAA | PcaK from *Pseudomonas putida* containing a helical domain from a *Rhodococcus pyridinivorans* TphK indicated accordingly: TM7 | |  |
| PcaK_TphK_TM10_ | | ATGAATCAGGCGCAAAATTCTGTAGGTAAAAGCTTAGACGTGCAGTCATTCATCAATCAACAACCCTTGTCGCGTTACCAGTGGCGCGTCGTGTTGTTATGCTTTTTAATTGTTTTTTTGGACGGATTAGATACCGCTGCAATGGGTTTCATTGCTCCAGCGTTATCTCAAGAATGGGGAATCGATCGTGCATCGTTAGGGCCTGTCATGAGCGCGGCCCTGATCGGCATGGTTTTTGGAGCCTTGGGGTCGGGTCCGCTGGCCGACCGTTTTGGCCGTAAGGGGGTTCTGGTCGGCGCAGTACTGGTCTTCGGGGGCTTCAGTTTAGCGAGCGCATACGCGACGAATGTCGATCAGCTGTTAGTCTTGCGTTTTCTGACTGGACTGGGTCTGGGCGCCGGGATGCCGAATGCGACGACCTTGTTAAGCGAGTACACACCTGAGCGCCTGAAGTCCTTACTTGTGACGAGTATGTTTTGTGGATTTAACCTTGGCATGGCCGGCGGTGGCTTTATTTCCGCGAAGATGATTCCCGCCTACGGATGGCATAGCTTACTGGTCATTGGAGGTGTCCTGCCCTTGTTGTTAGCTCTGGTCTTGATGGTGTGGTTACCGGAGAGCGCCCGCTTTCTTGTAGTCCGTAACCGTGGGACGGATAAAATTCGTAAAACCTTGTCCCCTATTGCCCCGCAGGTTGTTGCAGAAGCAGGATCTTTTTCCGTGCCTGAACAAAAAGCTGTCGCGGCATGTAGTGTCTTCGCAGTTATCTTCAGCGGGACCTATAGGCTTGGCACAATGTTACTTTGGTTGACCTATTTTATGGGGCTTGTCATCGTATACTTACTGACTTCATGGTTGCCAACTCTGATGCGTGACTCCGGGGCTTCGATGGAACAAGCAGCCTTCATCGGGGCTTTATTTCAGTTCGGTGGGGTGTTATCCGCTGTAGGCGTCGGTTGGGCGATGGACCGCTACAATCCTCATAAGGTCATCGGAATCTTTTACTTGTTGGCAGGAGTTTTTGCGTATGCAGTTGGACAGTCCCTGGGCGCACCCCTGTGGGTCCTCCTGACCGCAAACTTCTTCGCCGGGGTGTGCATTTCGGGCGGTCAAAAATCGCTCATCGCTTTGTCGGCCGTGTTCTATCCAACGTCGATGCGGTCGACGGGTGTGGGTTGGATGTTAGGTATCGGTCGCTTCGGCGCCATTTTGGGGGCTTGGTCCGGAGCCACGCTTTTAGGTCTTGGGTGGAACTTTGAACAAGTGCTTACGGCCCTGCTTGTCCCTGCAGCATTGGCCACGGTAGGCGTGATCGTTAAGGGGCTTGTTAGCCATGCGGACGCTACATAA | PcaK from *Pseudomonas putida* containing a helical domain from a *Rhodococcus pyridinivorans* TphK indicated accordingly: TM10 | |  |
| TphK_Pcak_TM1_ | | ATGAATCAGGCGCAAAATTCTGTAGGTAAAAGCTTAGACGTGCAGTCATTCATCAATCAACAACCCTTGTCGCGTTACCAGTGGCGCGTCGTGTTGTTATGCTTTTTAATTGTTTTTTTGGACGGATTAGATACCGCTGCAATGGGTTTCATTGCTCCAGCGTTATCTCAAGAATGGGGAATCGATCGTGCAGTCTTGGGCCCTATCTTTAGCGCCGCTTTGGCAGGCCTGATGGTCGGTTACCTGGCGCTCAGCCCCTTGAGCGAGCGGTTCGGGCACCGGCGTATGATCATCGCTAGCACCGTCCTCTTTGCACTCGGGACCCTCGCTGCAGCCTGGTCGCAAAACGTCACGGAATTGATGGCTTTGCGTTTCATCACCGGGATGGGCCTCGGGGCGGCGGCTCCTTCGGCTATCGCGTTGACCGGTGAGTACTCGCCCAAACGTTTGCGTGCTACGTTCGTGTTGGCCATCTACTGCGGGTTTAGCTTGGGCTTTGTCGCTGCGGGGTTGGTCTCGGGCTGGCTCATTCCGATTTTTGGGTGGCGTTCGGTCCTCGTCGTGGGTGCGGTCGCCCCACTCTTGTTGCTCCCGGCCCTCCTCCGGTTCCTCCCTGATAGCCTCACCCTGATGATTAACAAGGGCGCAGGTCCAGAACGTGTCCGTAGCATTCTCCGTCGCATTGATCCAGGCTTGGCTGCCGGCAGCGATTTTACGTATGAAGCCGAGACCCAGCCTGATGAGCAACGGACCGCCTTGCGGAACTTGTTCACCCGGGACCGTGTCCTGGGTACCTTGCTGCTGTGGCTCGTGTTTGTGATTAATTTGGGTGAATTTTACGCACTCCAGTCCTGGCTCCCGAGCATTATGACGAGCCTCGACTACGATATGGGTACCGTCGTCACGGCCACGACGCTGACCACGGTCGGGGGCATCGCTGCAGCTTTTGTGACGGGCCCGTGCATGGACCGTCTGGGGGCCTATTTGACGTTGGGGACGGTCTACATCGTCGGTTTTGCCTTTGTGGCGCTGACGGGTTTGGCCTTGTCCGCACCCCTGTGGGTCCTCCTGACCGCAAACTTCTTCGCCGGGGTGTGCATTTCGGGCGGTCAAAAATCGCTCATCGCTTTGTCGGCCGTGTTCTATCCAACGTCGATGCGGTCGACGGGTGTGGGTTGGGCTCTCGGTGTGGGTCGGATTGGGGGGATTGTCGGTCCTATTGTGGTCGGTGCCGCTCTGGGCATGGGCTGGTCCGCGTCCGCGGTGTTCTATGCAATGTCGATTCCTATGCTCGTGGCGGGTGTCGCGGTGCTCCTGCTCGGTCGTTGGGTGCGTAGCGACAAACACCCAGATCGTGCTGTGGCGGAATCGCAGAGCTTGGCCCACAAGTAG | TphK from *Rhodococcus pyridinivorans* containing a helical domain from *Pseudomonas putida* PcaK indicated accordingly: TM1 | |  |
| TphK_Pcak_TM4_ | | ATGTCCATTGCTCCGTCCCGCGTGACCCTGCCAGATTTCATTGACTCCCGGCCAGTGAGCCGGTATCAATATATTGTGATTGGTTTGTGCGGGCTCGTGATGTTTATTGACGGGTTCGATACGCAATCGATCTCGTATATGGCACCTCACATCGCTGAGGAATGGGGGCTGAGCAAGCAAGTCTTGGGCCCTATCTTTAGCGCCGCTTTGGCAGGCCTGATGGTCGGTTACCTGGCGCTCAGCCCCTTGAGCGAGCGGTTCGGGCACCGGCGTATGATCATCGCTAGCACCGTCCTCTTTGCACTCGGGACCCTCGCTGCAGCCTGGTCGCAAAATGTCGATCAGCTGTTAGTCTTGCGTTTTCTGACTGGACTGGGTCTGGGCGCCGGGATGCCGAATGCGACGACCTTGTTAAGCGAGTACACACCTGAGCGCCTGAAGTCCTTACTTGTGTTGGCCATCTACTGCGGGTTTAGCTTGGGCTTTGTCGCTGCGGGGTTGGTCTCGGGCTGGCTCATTCCGATTTTTGGGTGGCGTTCGGTCCTCGTCGTGGGTGCGGTCGCCCCACTCTTGTTGCTCCCGGCCCTCCTCCGGTTCCTCCCTGATAGCCTCACCCTGATGATTAACAAGGGCGCAGGTCCAGAACGTGTCCGTAGCATTCTCCGTCGCATTGATCCAGGCTTGGCTGCCGGCAGCGATTTTACGTATGAAGCCGAGACCCAGCCTGATGAGCAACGGACCGCCTTGCGGAACTTGTTCACCCGGGACCGTGTCCTGGGTACCTTGCTGCTGTGGCTCGTGTTTGTGATTAATTTGGGTGAATTTTACGCACTCCAGTCCTGGCTCCCGAGCATTATGACGAGCCTCGACTACGATATGGGTACCGTCGTCACGGCCACGACGCTGACCACGGTCGGGGGCATCGCTGCAGCTTTTGTGACGGGCCCGTGCATGGACCGTCTGGGGGCCTATTTGACGTTGGGGACGGTCTACATCGTCGGTTTTGCCTTTGTGGCGCTGACGGGTTTGGCCTTGTCCGCACCCCTGTGGGTCCTCCTGACCGCAAACTTCTTCGCCGGGGTGTGCATTTCGGGCGGTCAAAAATCGCTCATCGCTTTGTCGGCCGTGTTCTATCCAACGTCGATGCGGTCGACGGGTGTGGGTTGGGCTCTCGGTGTGGGTCGGATTGGGGGGATTGTCGGTCCTATTGTGGTCGGTGCCGCTCTGGGCATGGGCTGGTCCGCGTCCGCGGTGTTCTATGCAATGTCGATTCCTATGCTCGTGGCGGGTGTCGCGGTGCTCCTGCTCGGTCGTTGGGTGCGTAGCGACAAACACCCAGATCGTGCTGTGGCGGAATCGCAGAGCTTGGCCCACAAGTAG | TphK from *Rhodococcus pyridinivorans* containing a helical domain from *Pseudomonas putida* PcaK indicated accordingly: TM4 | |  |
| TphK_PcaK_TM7_ | | ATGTCCATTGCTCCGTCCCGCGTGACCCTGCCAGATTTCATTGACTCCCGGCCAGTGAGCCGGTATCAATATATTGTGATTGGTTTGTGCGGGCTCGTGATGTTTATTGACGGGTTCGATACGCAATCGATCTCGTATATGGCACCTCACATCGCTGAGGAATGGGGGCTGAGCAAGCAAGTCTTGGGCCCTATCTTTAGCGCCGCTTTGGCAGGCCTGATGGTCGGTTACCTGGCGCTCAGCCCCTTGAGCGAGCGGTTCGGGCACCGGCGTATGATCATCGCTAGCACCGTCCTCTTTGCACTCGGGACCCTCGCTGCAGCCTGGTCGCAAAACGTCACGGAATTGATGGCTTTGCGTTTCATCACCGGGATGGGCCTCGGGGCGGCGGCTCCTTCGGCTATCGCGTTGACCGGTGAGTACTCGCCCAAACGTTTGCGTGCTACGTTCGTGTTGGCCATCTACTGCGGGTTTAGCTTGGGCTTTGTCGCTGCGGGGTTGGTCTCGGGCTGGCTCATTCCGATTTTTGGGTGGCGTTCGGTCCTCGTCGTGGGTGCGGTCGCCCCACTCTTGTTGCTCCCGGCCCTCCTCCGGTTCCTCCCGGAGAGCGCCCGCTTTCTTGTAGTCCGTAACCGTGGGACGGATAAAATTCGTAAAACCTTGTCCCCTATTGCCCCGCAGGTTGTTGCAGAAGCAGGATCTTTTTCCGTGCCTGAACAAAAAGCTGTCGCGGCATGTAGTGTCTTCGCAGTTATCTTCAGCGGGACCTATAGGCTTGGCACAATGTTACTTTGGTTGACCTATTTTATGGGGCTTGTCATCGTATACTTACTGACTTCATGGTTGCCAACTCTGATGCGTGACTCCGGGGCTTCGATGGAACAAGTCGTCACGGCCACGACGCTGACCACGGTCGGGGGCATCGCTGCAGCTTTTGTGACGGGCCCGTGCATGGACCGTCTGGGGGCCTATTTGACGTTGGGGACGGTCTACATCGTCGGTTTTGCCTTTGTGGCGCTGACGGGTTTGGCCTTGTCCGCACCCCTGTGGGTCCTCCTGACCGCAAACTTCTTCGCCGGGGTGTGCATTTCGGGCGGTCAAAAATCGCTCATCGCTTTGTCGGCCGTGTTCTATCCAACGTCGATGCGGTCGACGGGTGTGGGTTGGGCTCTCGGTGTGGGTCGGATTGGGGGGATTGTCGGTCCTATTGTGGTCGGTGCCGCTCTGGGCATGGGCTGGTCCGCGTCCGCGGTGTTCTATGCAATGTCGATTCCTATGCTCGTGGCGGGTGTCGCGGTGCTCCTGCTCGGTCGTTGGGTGCGTAGCGACAAACACCCAGATCGTGCTGTGGCGGAATCGCAGAGCTTGGCCCACAAGTAG | TphK from *Rhodococcus pyridinivorans* containing a helical domain from *Pseudomonas putida* PcaK indicated accordingly: TM7 | |  |
| TphK_PcaK_TM10_ | | ATGTCCATTGCTCCGTCCCGCGTGACCCTGCCAGATTTCATTGACTCCCGGCCAGTGAGCCGGTATCAATATATTGTGATTGGTTTGTGCGGGCTCGTGATGTTTATTGACGGGTTCGATACGCAATCGATCTCGTATATGGCACCTCACATCGCTGAGGAATGGGGGCTGAGCAAGCAAGTCTTGGGCCCTATCTTTAGCGCCGCTTTGGCAGGCCTGATGGTCGGTTACCTGGCGCTCAGCCCCTTGAGCGAGCGGTTCGGGCACCGGCGTATGATCATCGCTAGCACCGTCCTCTTTGCACTCGGGACCCTCGCTGCAGCCTGGTCGCAAAACGTCACGGAATTGATGGCTTTGCGTTTCATCACCGGGATGGGCCTCGGGGCGGCGGCTCCTTCGGCTATCGCGTTGACCGGTGAGTACTCGCCCAAACGTTTGCGTGCTACGTTCGTGTTGGCCATCTACTGCGGGTTTAGCTTGGGCTTTGTCGCTGCGGGGTTGGTCTCGGGCTGGCTCATTCCGATTTTTGGGTGGCGTTCGGTCCTCGTCGTGGGTGCGGTCGCCCCACTCTTGTTGCTCCCGGCCCTCCTCCGGTTCCTCCCTGATAGCCTCACCCTGATGATTAACAAGGGCGCAGGTCCAGAACGTGTCCGTAGCATTCTCCGTCGCATTGATCCAGGCTTGGCTGCCGGCAGCGATTTTACGTATGAAGCCGAGACCCAGCCTGATGAGCAACGGACCGCCTTGCGGAACTTGTTCACCCGGGACCGTGTCCTGGGTACCTTGCTGCTGTGGCTCGTGTTTGTGATTAATTTGGGTGAATTTTACGCACTCCAGTCCTGGCTCCCGAGCATTATGACGAGCCTCGACTACGATATGGGTACCGTCGTCACGGCCACGACGCTGACCACGGTCGGGGGCATCGCTGCAGCTTTTGTGACGGGCCCGTGCATGGACCGTCTGGGGGCCTATTTGACGTTGGGGACGGTCTACATCGTCGGTTTTGCCTTTGTGGCGCTGACGGGTTTGGCCTTGTCCAACATCACTGTTTTAGCAACTTTAGTGCTGATCGCTGGGATGTGTGTTAACGGCGCCCAGTCGGCCATGCCGAGCCTTGCAGCGCGTTTTTATCCTACGCAGGGACGTGCGACGGGGGTAAGCTGGGCTCTCGGTGTGGGTCGGATTGGGGGGATTGTCGGTCCTATTGTGGTCGGTGCCGCTCTGGGCATGGGCTGGTCCGCGTCCGCGGTGTTCTATGCAATGTCGATTCCTATGCTCGTGGCGGGTGTCGCGGTGCTCCTGCTCGGTCGTTGGGTGCGTAGCGACAAACACCCAGATCGTGCTGTGGCGGAATCGCAGAGCTTGGCCCACAAGTAG | TphK from *Rhodococcus pyridinivorans* containing a helical domain from *Pseudomonas putida* PcaK indicated accordingly: TM10 | |  |

### Table S4 – TPA analogue library used in transporter screening

| **CAS No.** | **Substrate** | | **Structure** |
| --- | --- | --- | --- |
| **65-85-0** | T2 | Benzoic acid | 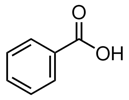 |
| **100-21-0** | T3 | Terephthalic acid (TPA) | 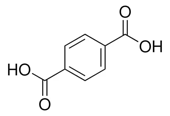 |
| **100-26-5** | T4 | 2,5-Pyridinedicarboxylic acid | 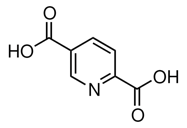 |
| **100-26-5** | T5 | 2,5-Pyrazinedicarboxylic acid | 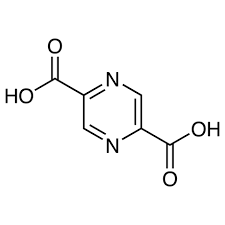 |
| **3238-40-2** | T6 | 2,5-Furandicarboxylic acid | 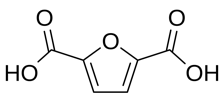 |
| **121-91-5** | T7 | Isophthalic acid | 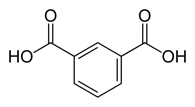 |
| **490-11-9** | T8 | 3,4-Pyridinedicarboxylic acid | 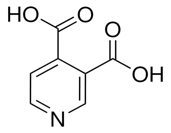 |
| **89-00-9** | T9 | 2,3-Pyridinedicarboxylic acid | 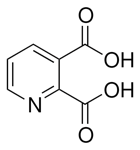 |
| **59648-14-5** | T10 | Pyridizine-4,5-dicarboxylic acid | 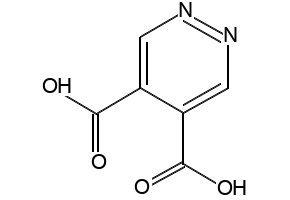 |
| **88-99-3** | T11 | Phthalic acid | 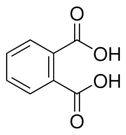 |
| **3387-26-6** | T12 | 3,4-Furandicarboxylic acid | 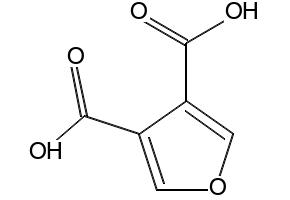 |
| **636-94-2** | T13 | 2-Hydroxyterephthalic acid (2-Hydroxy TPA) | 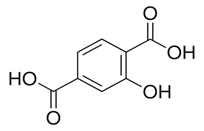 |
| **610-92-4** | T14 | 2,5-Dihydroxyterephthalic acid | 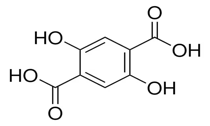 |
| **586-35-6** | T15 | 2-Bromoterephthalic acid | 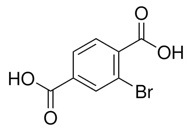 |
| **1829-22-7** | T16 | 2-Iodoterephthalic acid | 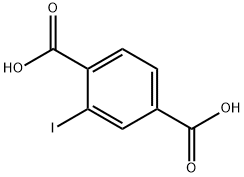 |
| **5411-70-1** | T17 | Tetrabromoterephthalic acid | 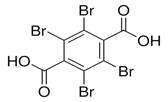 |
| **10312-55-7** | T18 | 2-Aminoterephthalic acid | 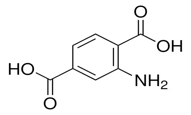 |
| **610-29-7** | T19 | 2-Nitroterephthalic acid | 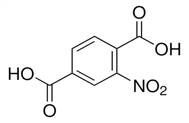 |
| **787-70-2** | T20 | Biphenyl-4,4’-dicarboxylic acid | 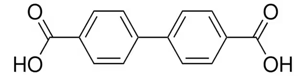 |
| **1141-38-4** | T21 | Naphthalene-2,6-dicarboxylic acid | 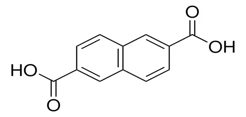 |
| **605-70-9** | T22 | Naphthalene-1,4-dicarboxylic acid | 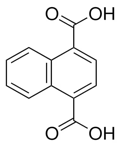 |
| **99-50-3** | T23 | Protocatechuic acid (PCA) | 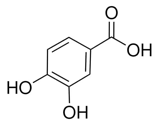 |
| **1119-72-8** | T24 | *Cis,cis-*muconic acid | 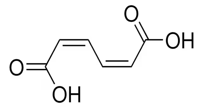 |
| **99-06-9** | T25 | 3-Hydroxybenzoic acid (3HBA) | 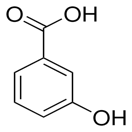 |
| **121-34-6** | T26 | Vanillic acid | 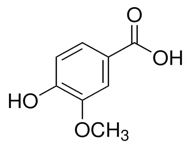 |
| **149-91-7** | T27 | Gallic acid | 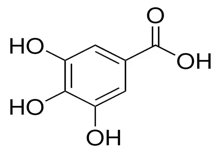 |
| **99-96-7** | T28 | 4-Hydroxybenzoic acid (4HBA) | 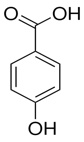 |

### Table S5 – PCA analogue library used in transporter screening

| **CAS No.** | **Substrate** | | **Structure** |
| --- | --- | --- | --- |
| **99-50-3** | P2 | Protocatechuic acid (PCA) | 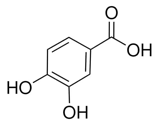 |
| **99-10-5** | P3 | 3,5-Dihydroxybenzoic acid | 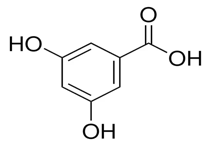 |
| **69-72-7** | P4 | Salicylic acid | 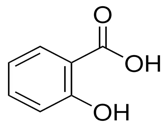 |
| **89-86-1** | P5 | 2,4-Dihydroxybenzoic acid | 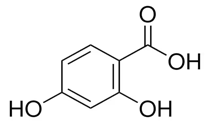 |
| **490-79-9** | P6 | 2,5-Dihydroxybenzoic acid | 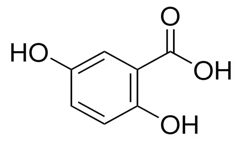 |
| **645-08-9** | P7 | Isovanillic acid | 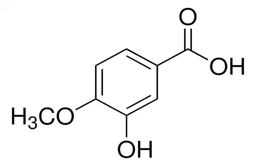 |
| **530-57-4** | P8 | Syringic acid | 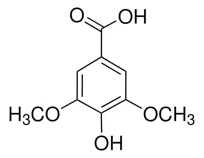 |
| **100-09-4** | P9 | 4-Methoxybenzoic acid | 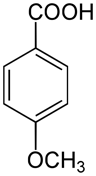 |
| **3934-84-7** | P10 | 3,4-Dihydroxy-5-methoxybenzoic acid (3-O-methylgallic acid) | 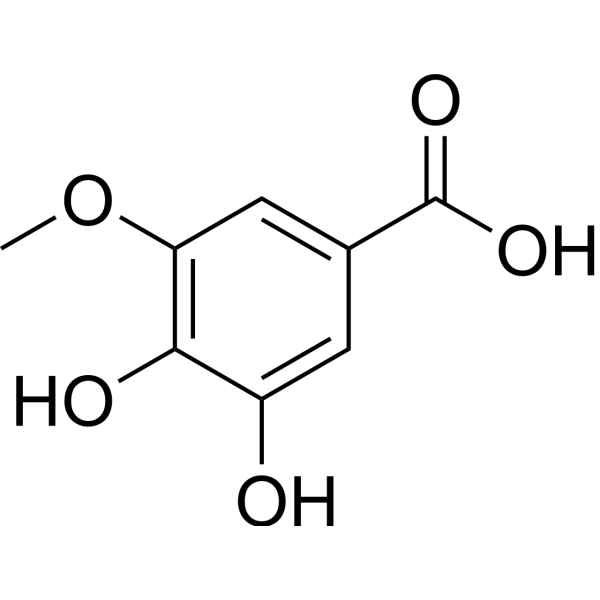 |
| **573-11-5** | P11 | 2,3,4-Trimethoxybenzoic acid | 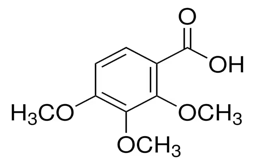 |
| **877-22-5** | P12 | 2,3-Methoxybenzoic acid | 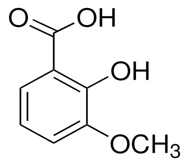 |
| **8-1-1916** | P13 | 5-Hydroxyveratric acid | 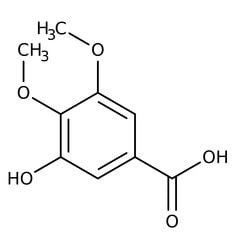 |
| **535-80-8** | P14 | 3-Chlorobenzoic acid | 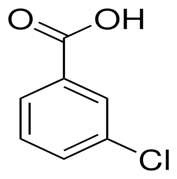 |
| **2150-43-8** | P15 | Methyl-3,4-dihydroxybenzoic acid | 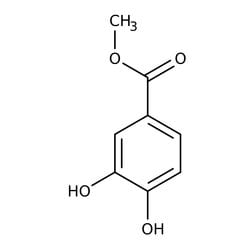 |
| **54337-90-5** | P16 | 3,4-Dihydroxybenzamide | 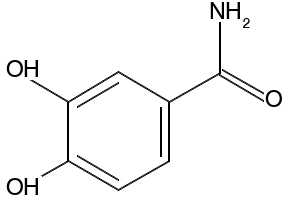 |
| **123-08-0** | P17 | 4-hydroxybenzaldehyde | 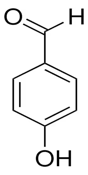 |
| **3424-93-9** | P18 | 4-methoxybenzamide | 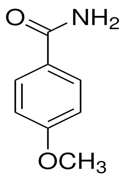 |
| **100-02-7** | P19 | p-nitrophenol | 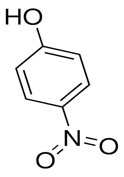 |
| **88-99-3** | P20 | Phthalic acid | 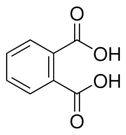 |
| **3238-40-2** | P21 | 2,5-Furandicarboxylic acid | 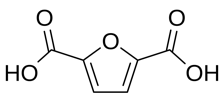 |
| **106-50-3** | P22 | *p-*phenylenediamine | 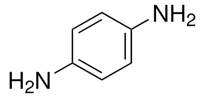 |
| **121-33-5** | P23 | Vanillin | 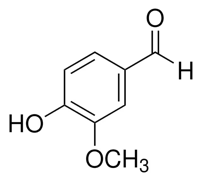 |
| **99-06-9** | P24 | 3-Hydroxybenzoic acid (3HBA) | 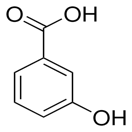 |
| **99-96-7** | P25 | 4-Hydroxybenzoic acid (4HBA) | 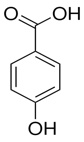 |
| **121-34-6** | P26 | Vanillic acid | 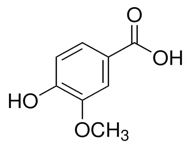 |
| **501-98-4** | P27 | *p-*Coumaric acid | 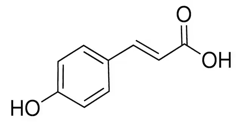 |
| **149-91-7** | P28 | Gallic acid | 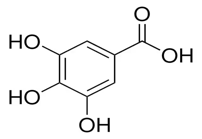 |
| **537-98-4** | P29 | *Trans*-Ferulic acid | 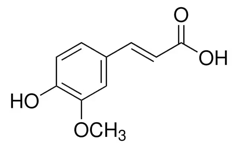 |


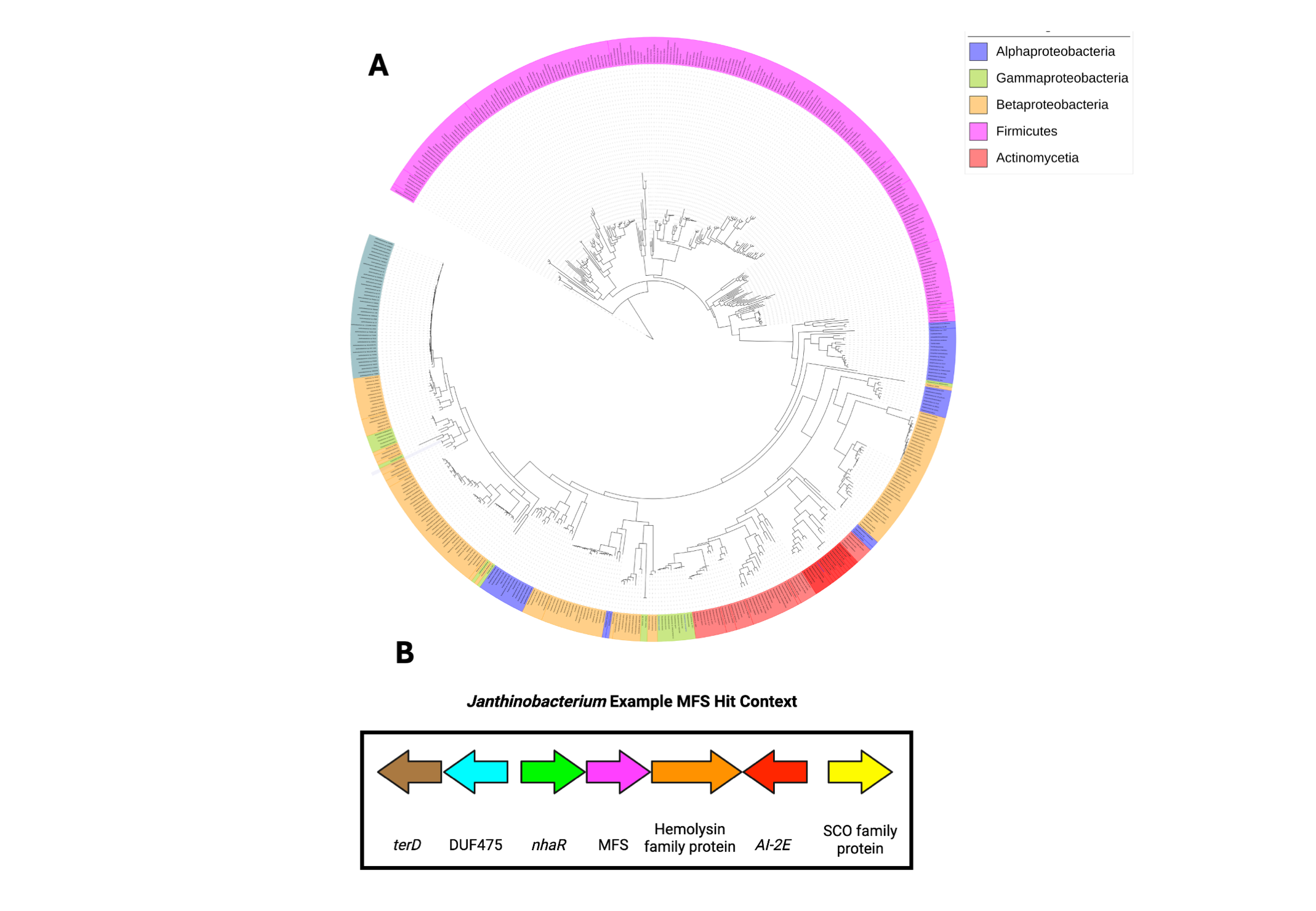


**Supp Fig. 1 – Phylogenetic Tree of Rhodococcus jostii RHA1 BlastP results and example MFS hit context.** (**A**) Plotted tree of blastP results using the tphK gene from the R. jostii RHA1 TPA catabolic operon as a query sequence totalling 530 leaves. Coloured ranges demonstrate phylum of bacteria with the tree rooted with respect to R. jostii RHA1 sequence. Sequences belonging to the genus Janthinobacterium are highlighted in teal. Sequences featured in tree possess at least 40% identity to query sequence. (**B**) Typical genetic context of the MFS hits belonging to the Janthinobacterium genus demonstrating no colocalization to any TPA catabolic genes. Orientations and annotations are provided within the subplot.

**Supp Fig. 2 – Comparative growth and PCA consumption of WT and ΔpcaK P. putida strains on PCA as a sole carbon source and PCA cytotoxicity. (A)** Comparative OD_600_ measurements of wildtype P. putida and ΔpcaK:P. putida when grown on M9 minimal media supplemented with 10mM of PCA. **(B)** Consumption rate of PCA over 24 hours comparing wildtype and mutant P. putida. **(C)** Bar plot comparing the first 4 hours of PCA consumption between the two strains. (**D**) Comparative bar plot of final OD_600_ reading for the PcaK_-ve_ and PcaK_P.Put_ biosensor constructs when exposed to 0mM or 5mM of PCA. Unpaired t-testing analysis was performed to assess impact.


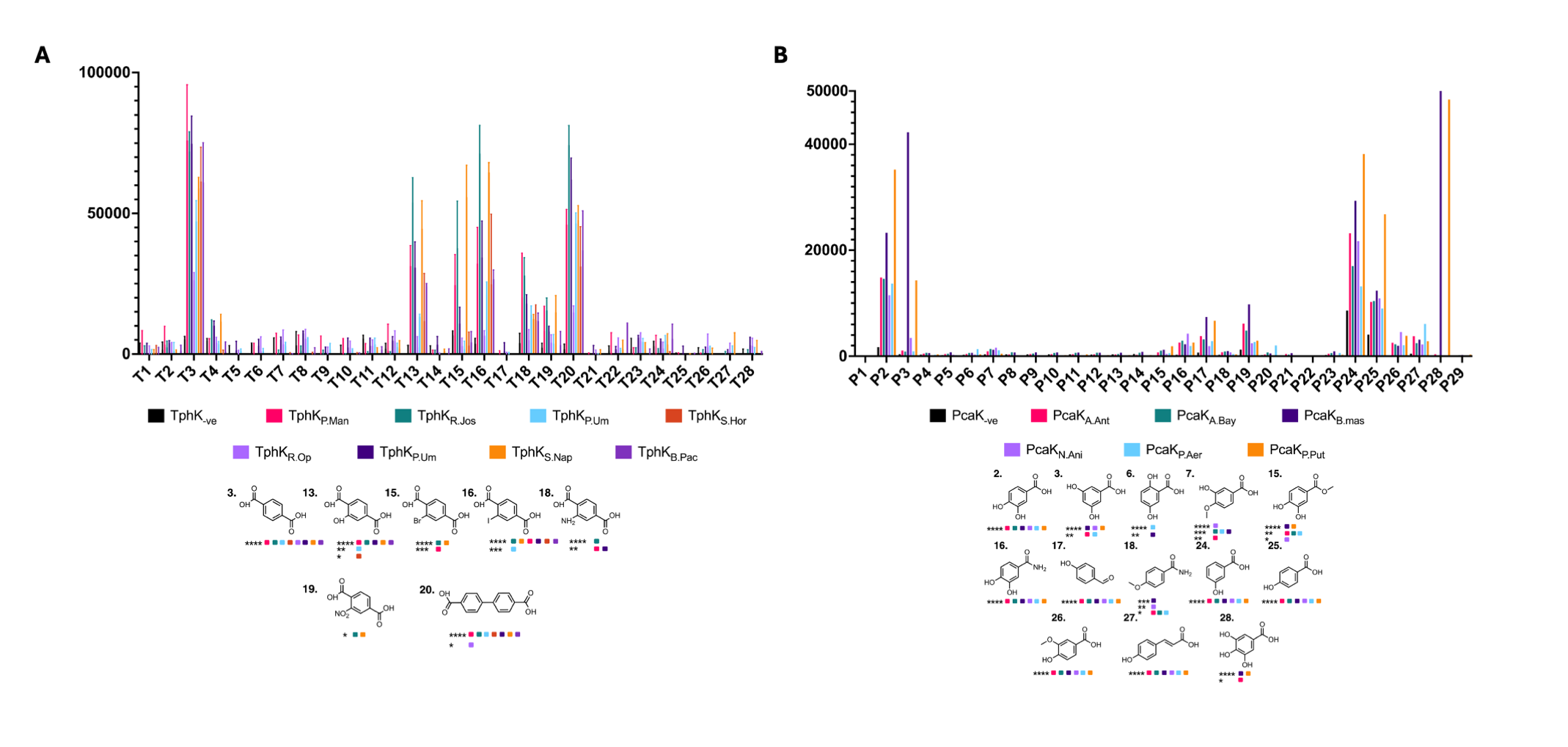


**Supp Fig. 3 – Raw RFU/OD data of functional TphK and PcaK biosensor construct screening against analogue libraries. (A)** Screening of functionally verified TphK transporters against a library of TPA analogues when normalised for basal activity in the uninduced state (T1). **(B)** Screening of functionally verified PcaK transporters against a library of PCA analogues when normalised for basal activity in the uninduced state (P1). Active compound structures are annotated next to the graphs with the degree of significance shown next to the legend colour.


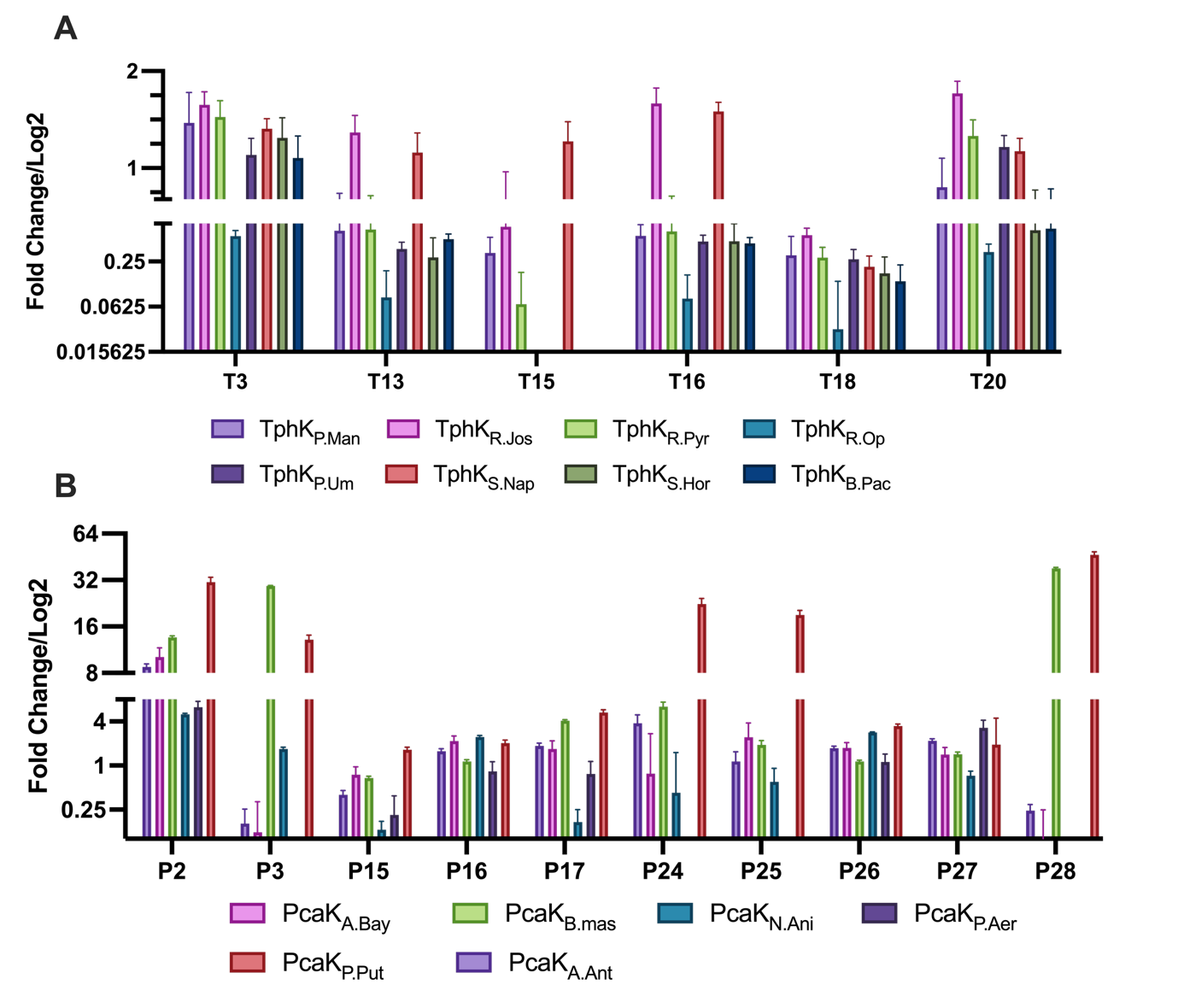


**Supp Fig. 4 – No transporter normalised effector screening data of TphK and PcaK biosensor constructs. (A)** Induced fold change for effectors that generated significant activity to at least one of the TphK biosensor constructs for the TPA analogue. **(B)** Induced fold change for effectors that generated significant activity to at least one of the PcaK biosensor constructs for the PCA analogue library. Data was normalised to the no transporter control via subtraction of this response yielding only the net effect on biosensor activation as graphed.

**Supp Fig. 5 – Comparative analytics of 4,4-biphenyldicarboxylic acid consumption over 24 hours in *P. putida*.** Analysis of 4,4-biphenyldicarboxylic acid consumption over 24 hours in wildtype *P. putida* compared to *P. putida* transformed with the R.Pyr MFS bearing plasmid. HPLC analytics of culture supernatant were performed to monitor any loss of substrate indicating catabolism.

**Supp Fig. 6 – Induction profile of active PcaKs against 5mM of Benzoic acid.** (**A**) Comparative plot of PcaK_P.Put_ biosensor activation in the presence of 5mM of PCA compared to benzoic acid as well as when no substrate was added (Uninduced). (**B**) Comparative activation of active PcaK constructs in uninduced versus 5mM of benzoic acid. Fluorescence values were normalised according to OD and calculated as an average of 3 biological replicates


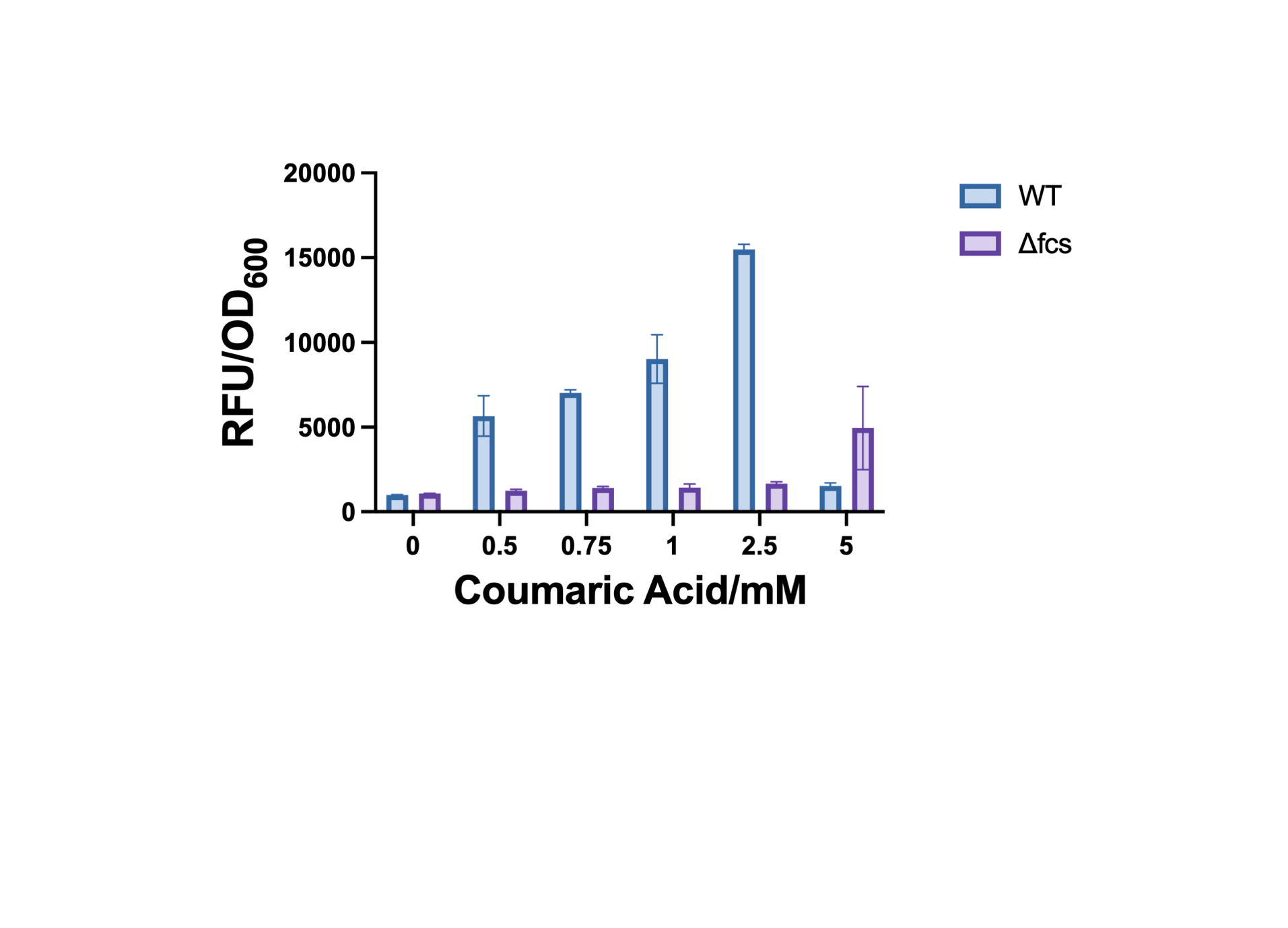


**Supp Fig. 7 – Comparison of activation of pcaV biosensor construct in Wildtype vs Δfcs P. putida.** Comparative activation of pcaV biosensor construct in wildtype putida vs Δfcs P. putida. Bars represent biological replicates of 3 for each concentration of coumaric acid tested.


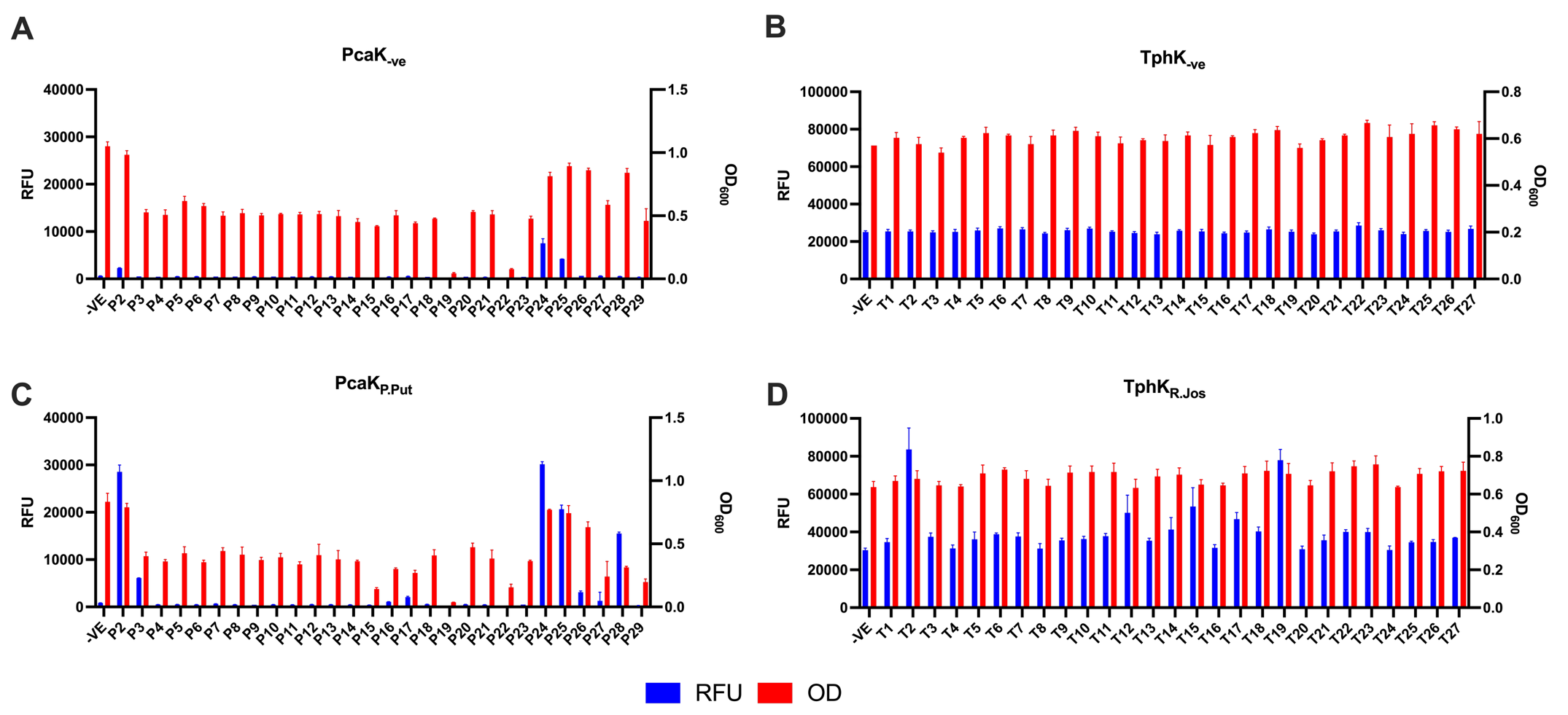


**Supp Fig. 8 – Comparison of raw RFU vs OD_600_ data for PcaK_-ve_ , PcaK_P.Put_ TphK_-ve_ and TphK_R.Jos_.** (**A**) Final recorded RFU and OD_600_ readings for the PcaK_-ve_ biosensor construct when exposed to no effector (-VE) or the PCA analogue library. (**B**) Final recorded RFU and OD_600_ readings for the TphK_-ve_ biosensor construct when exposed to no effector (-VE) or the TPA analogue library. (**C**) Complementary final recorded RFU and OD_600_ readings for the PcaK_P.Put_ biosensor construct when exposed to no effector (-VE) or the PCA analogue library. (**D**) Complementary final recorded RFU and OD_600_ readings for the TphK_R.Jos_ biosensor construct when exposed to no effector (-VE) or the TPA analogue library. Bars are plotted from 3 biological replicates used to calculated standard deviation.


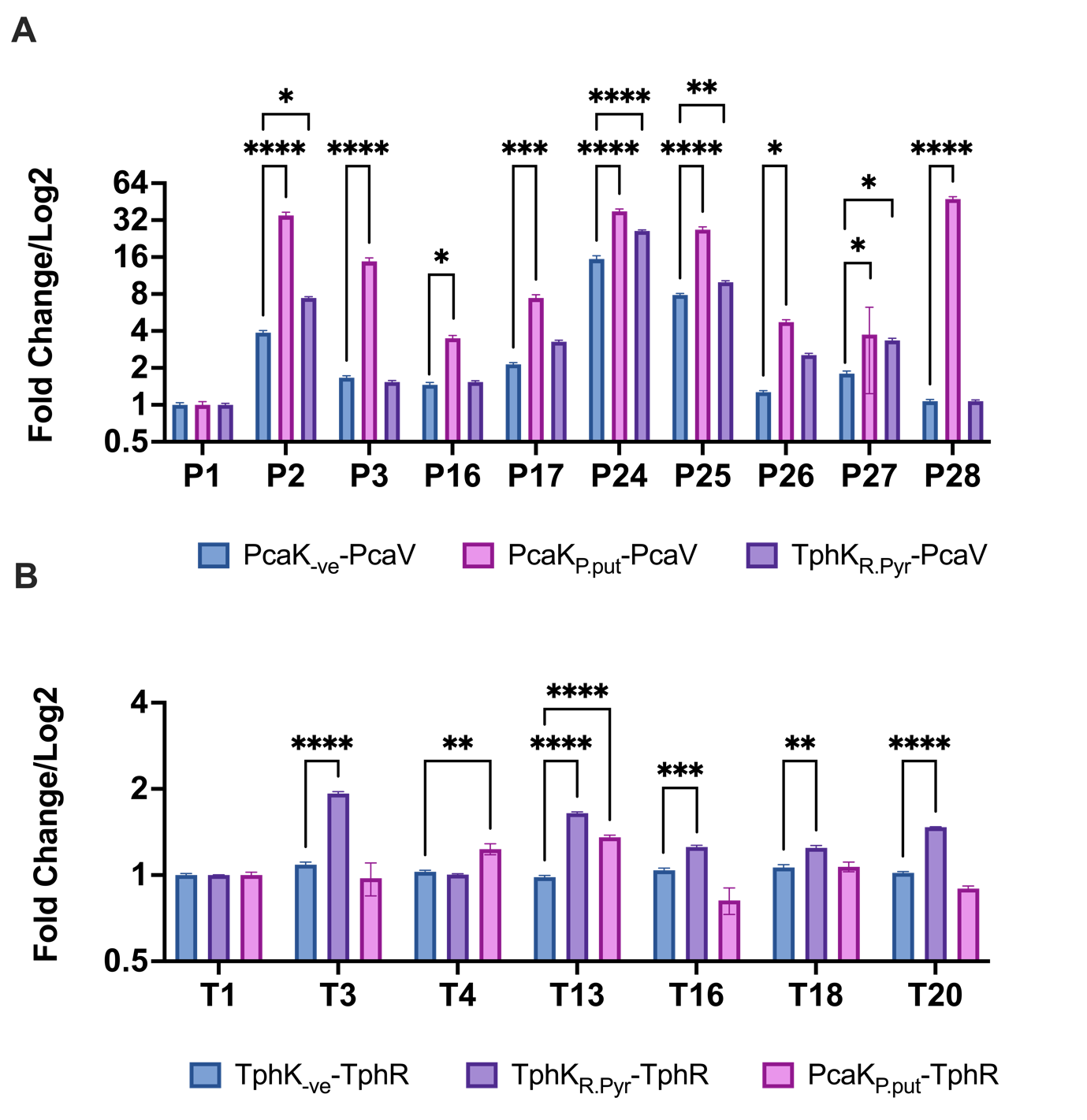


**Supp Fig. 9 – Screening of TphK_R.Pyr_-PcaV and PcaK_P.Put_-TphR against structural analogues of PCA and TPA.** (**A**) PCA analogues that elicited statistically significant fold changes of TphK_R.Pyr_-PcaV plotted on a log2 scale. (**B**) TPA analogues that elicited statistically significant fold changes of PcaK_P.put_-TphR. Plotted data denotes means of sample size n = 3 biological replicates using 2-way anova with multiple comparisons used to calculated statistical significance.


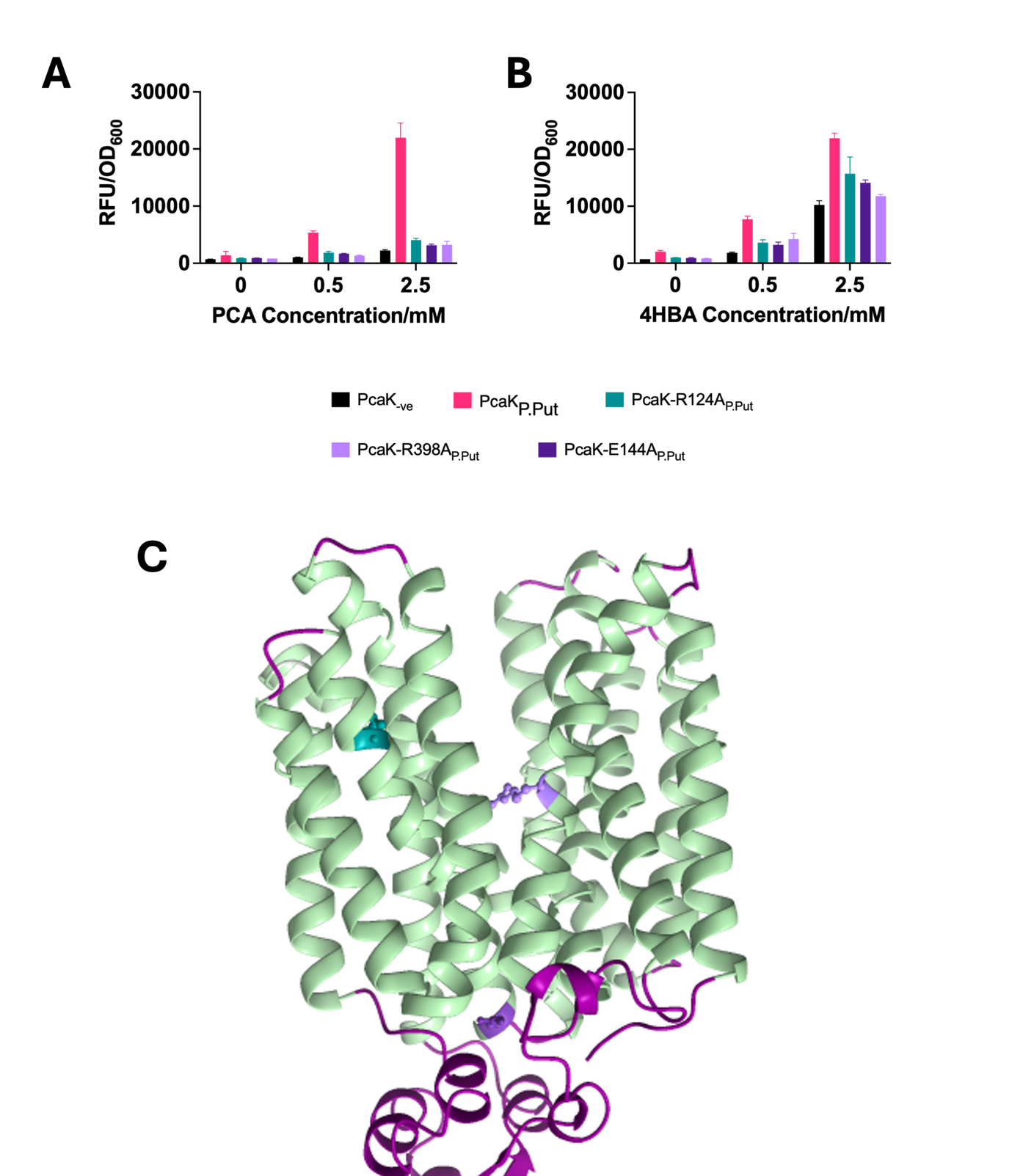


**Figure 10 – PcaK point mutant screening** (**A**) Screening of point mutants of PcaK_P.put_-PcaV against 3 concentrations of PCA using means of n = 3 biological replicates and statistical testing with 2-way Anova. (**B**) Screening of PcaK_P.Put_-PcaV against 3 concentrations of 4HBA using means of n = 3 biological replicates and statistical testing via 2-way Anova. (**C**) Alpha fold structure of PcaK_P.put_ (Q51955) showing positions of mutated residues R124 (Teal), E114 (Purple) and R398 (lilac). Alpha helical secondary structures corresponding to the helical domains of the MFS are shown in light green.


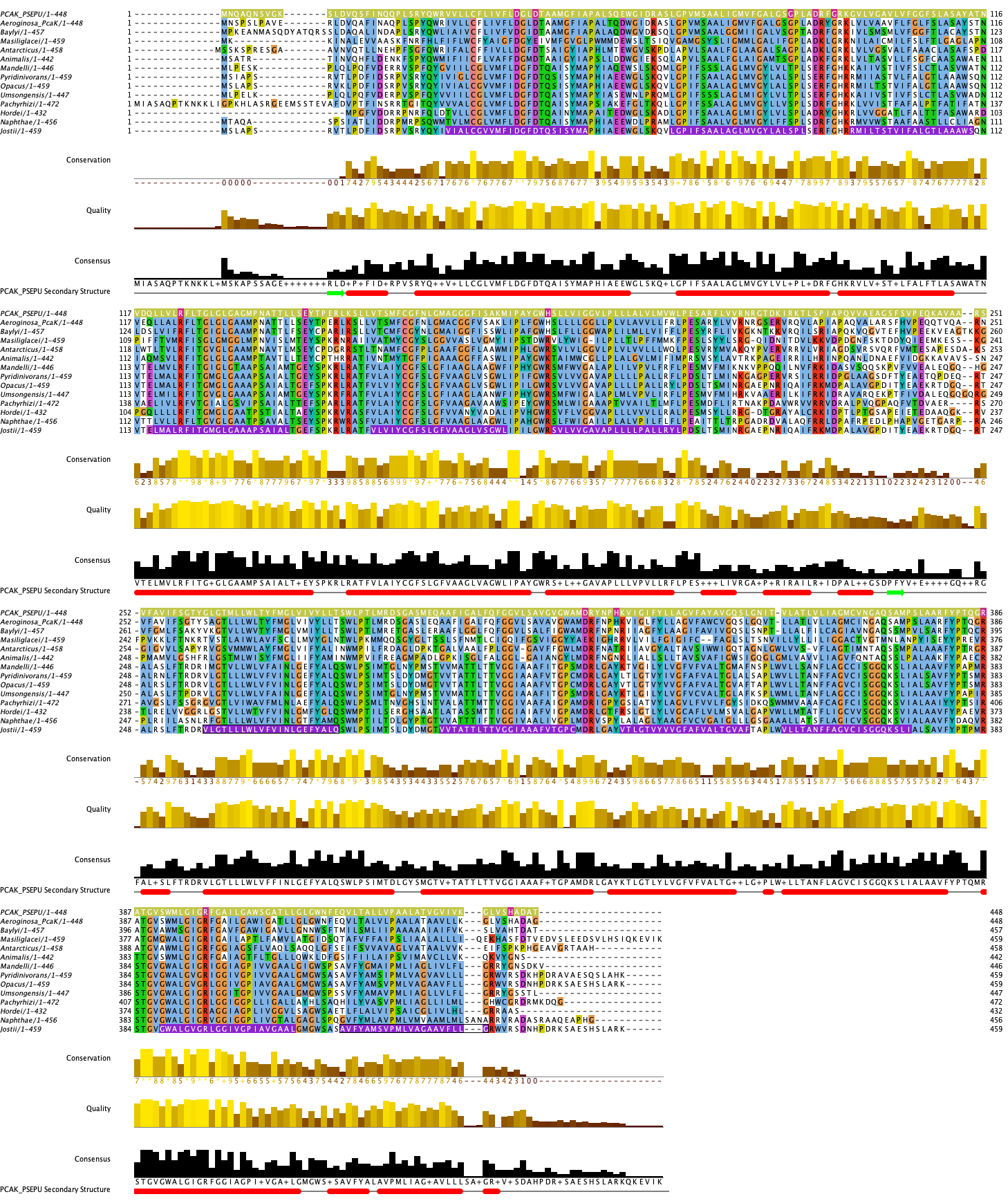
**Supp Fig. 11 – Multiple Sequence alignment and alphafold reference to assign helical domains for generation of chimeras.** Multiple sequence alignment derived following input through CC-TOP program to assign helical domains for chimeric exchange (highlighted purple) between PcaK_P.Put_ and TphK_R.Pyr_. To further aid domain assignment an alphafold model of PcaK from P. putida was added to the alignment in post analysis (Yellow highlighting) with secondary structure prediction overlayed (Red)


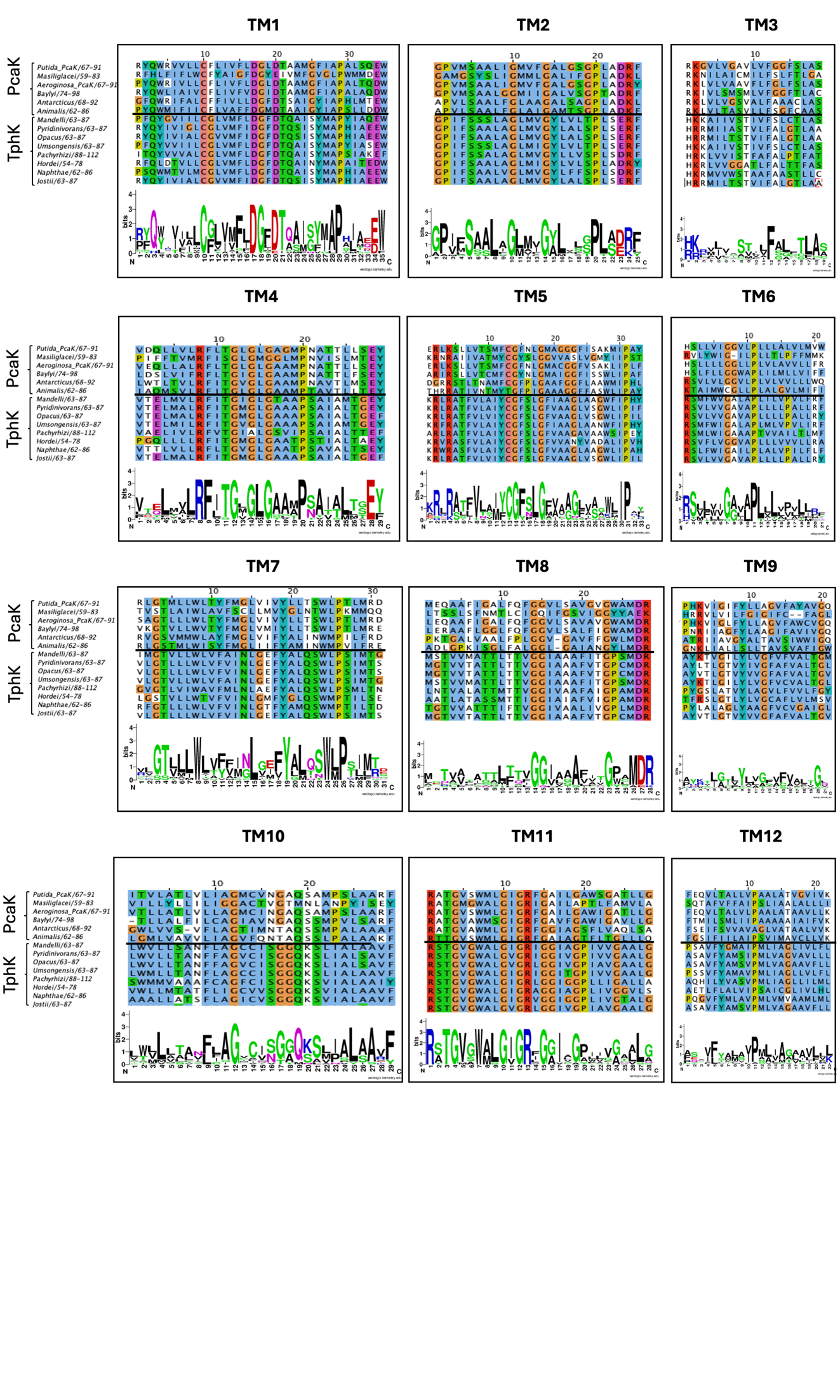


**Supp Fig. 12 – Validated PcaK and TphK TM region alignment and consensus logo analysis.** Plot of multiple sequence alignment data showing the extracted alignments per transmembrane domain. PcaK and TphK sequences are annotated with their strains of origin with a black line denoting the split between PcaK sequences and TphK sequences. Clustal colouring in Jalview was used to generate and colour the alignment.

**Supp Fig. 13 – MFS chimeric mutant assay normalised against the no transporter biosensor construct.** Complimentary plot of **Figure 4** presenting induction fold change data normalised against the effect of the no transporter biosensor construct for **(A)** the TphK biosensor constructs and **(B)** the PcaK biosensor constructs.


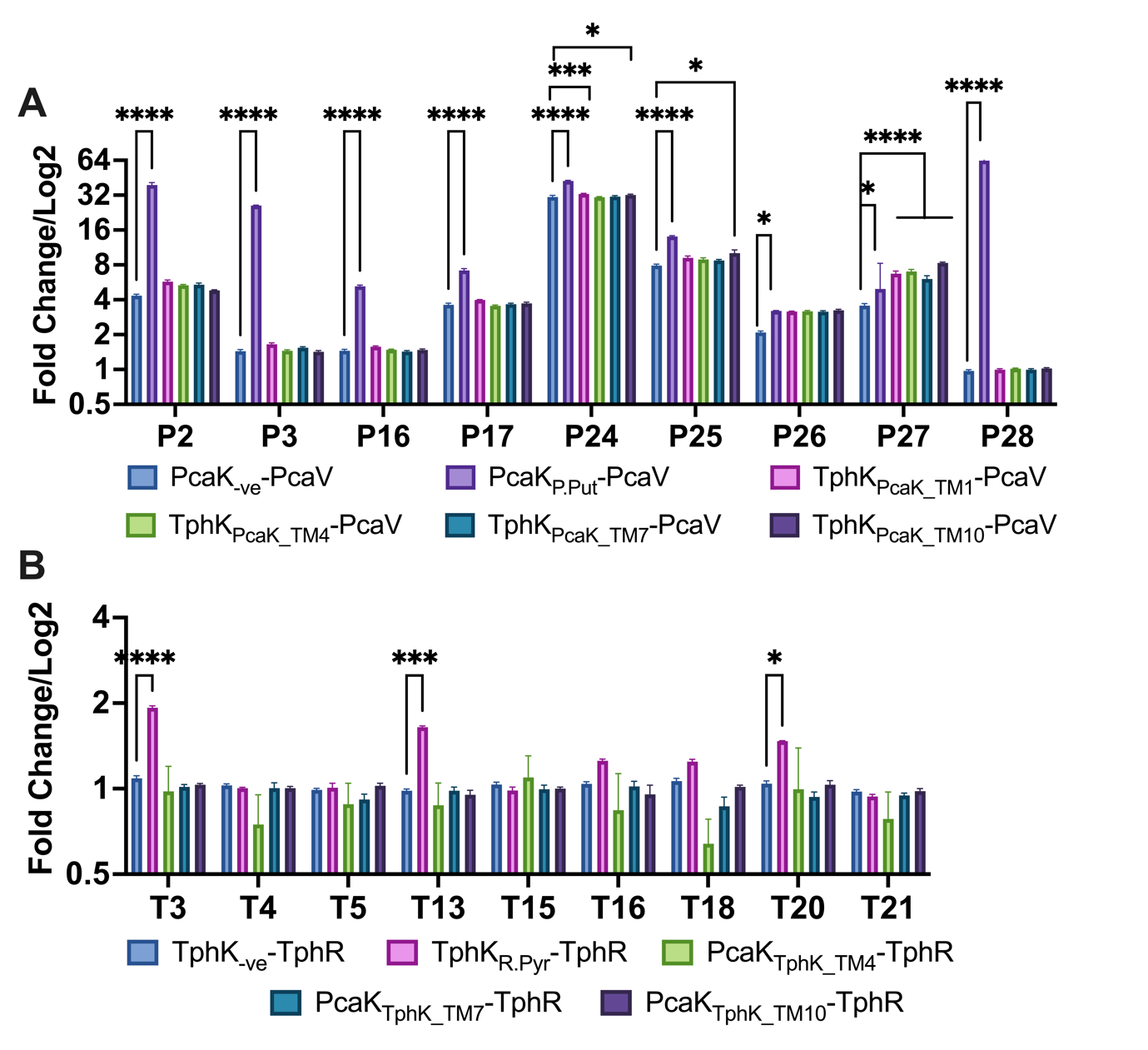


**Supp Fig. 14 – MFS chimeric mutant assay in opposing biosensor backgrounds.** (**A**) Comparative plot of TphK mutant activity towards active substrates from the PCA analogue library relative to the PcaK_-ve_ control. (**B**) Comparative plot of PcaK mutant activity towards active substrates from the TPA analogue library relative to the TphK_-ve_ control. 2-way anova was used to calculate statistically significant responses for both sets of data.
